# TRAF7-targeted HOXA5 acts as a tumor suppressor in prostate cancer progression and stemness via transcriptionally activating SPRY2 and regulating MEK/ERK signaling

**DOI:** 10.1038/s41420-023-01675-9

**Published:** 2023-10-16

**Authors:** Jianfeng Ye, Wangmin Liu, Xueyang Yu, Lina Wu, Zhengjie Chen, Yufei Yu, Jianfeng Wang, Song Bai, Mo Zhang

**Affiliations:** 1 Department of Urology, Shengjing Hospital of China Medical University, Shenyang, Liaoning China; 2 Department of Laboratory Medicine, Shengjing Hospital of China Medical University, Shenyang, Liaoning China; 3 Department of Urology, the First Hospital of China Medical University, Shenyang, Liaoning China

**Keywords:** Prostate cancer, Cancer genomics

## Abstract

Homeobox A5 (HOXA5), a homeodomain transcription factor, is considered a tumor suppressor in cancer progression; however, its function in prostate cancer (PCa) remains unclear. This study focused on the relevance of HOXA5 in PCa progression. We identified the downregulation of HOXA5 in PCa tissues based on the TCGA database and further verified in 30-paired PCa and adjacent normal tissues. Functional studies revealed that HOXA5 upregulation impaired the stem-like characteristics and malignant behaviors of PCa cells in vitro and in vivo. Mechanistically, HOXA5 was found to be regulated by tumor necrosis factor receptor-associated factor 7 (TRAF7), a putative E3-ubiquitin ligase. We observed that TRAF7 was overexpressed in PCa and subsequently enhanced the degradation of HOXA5 protein via its ubiquitin ligase activity, contributing to the acquisition of an aggressive PCa phenotype. For its downstream mechanism, we demonstrated that sprouty RTK signaling antagonist 2 (SPRY2) served as a downstream target of HOXA5. HOXA5 could directly bind to the SPRY2 promoter, thereby regulating the SPRY2-mediated MEK/ERK signaling pathway. Silencing SPRY2 largely compromised the tumor-suppressive effect of HOXA5 in PCa progression and cancer stemness. Our findings highlight the previously-underappreciated signaling axis of TRAF7–HOXA5–SPRY2, which provides a novel prognostic and therapeutic target for PCa treatment.

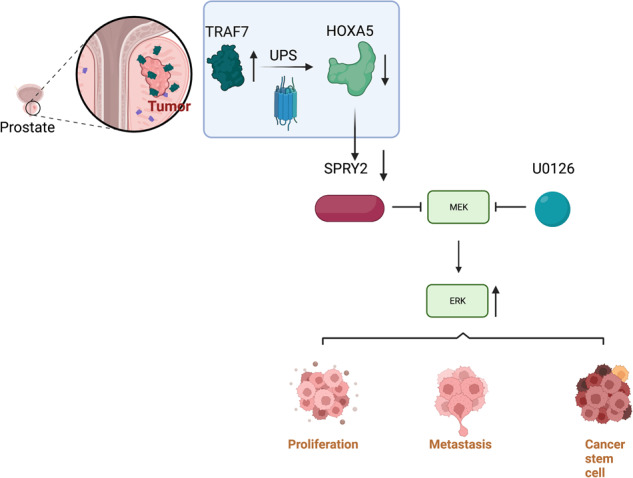

## Introduction

Prostate cancer (PCa) is one of the most prevalent malignancies affecting men worldwide and manifests tremendous biological heterogeneity [1, 2]. Despite the rapid development of therapeutic strategies, most men succumb to tumor recurrence and distant metastases [3]. Metastasis represents the primary catalyst of cancer-related mortality and the main barrier to treating late-stage PCa [4]. Emerging evidence has indicated that cancer stem cell-like cells (CSCs) contribute to PCa progression [5, 6]. In addition, the generation of stem cell properties was associated with cancer cell growth and metastasis [7]. Therefore, elucidating the molecular mechanisms underlying PCa progression and identifying novel therapeutic targets are urgently required to improve survival rates.

Homeobox A5 (HOXA5), a highly-conserved transcription factor, belongs to the HOX superfamily, which may be classified into clusters A–D and comprises 39 HOX genes [8]. The HOX family plays critical roles in organ development by regulating numerous processes, including apoptosis, receptor signaling, differentiation, and angiogenesis [9]. Aberrant HOX gene expression contributes to oncogenesis in various human malignancies [10, 11]. Nonetheless, the biological function of HOXA5 in cancer still remains controversial and varies among different organ types. HOXA5 is downregulated in colorectal cancer and prevents tumor progression and metastasis [12]. Conversely, HOXA5 exerts oncogenic function and is highly expressed in glioblastoma [13, 14]. The functional role and molecular mechanisms of HOXA5 in PCa remain unexplored. Besides, the upstream regulators and specific target genes of HOXA5 in PCa also have yet to be identified.

In order to identify proteins interacting with *homo* HOXA5, we first downloaded the list from the Hitpredict database (http://www.hitpredict.org/), then annotated these proteins into different Gene Ontology (GO) items at the biological process level. Impotantly, the proteins bound with HOXA5 were found to be enriched into ERK cascades, including tumor necrosis factor receptor-associated factor 7 (TRAF7). TRAF proteins function as E3 ubiquitin ligases and are involved in multiple biological processes [15, 16]. Accumulating evidence suggests that TRAF7 regulates tumor progression, inflammatory response, and apoptosis [17–19]. TRAF7 also reportedly drives oncogenic transformation in hepatocellular carcinoma and breast cancer through the ubiquitin-mediated degradation of target proteins, especially for transcription factors [20–22]. Notably, TRAF-associated proteins have previously been identified as interactors of HOX family [22]. It is unclear, however, if TRAF7 serves as an upstream regulator of HOXA5 and participates in the regulatory mechanisms underlying PCa progression.

As a latent transcription factor, HOXA5 functions by transcriptionally regulating potential downstream targets [14]. Interestingly, a microarray conducted by Huelsken J [12]. suggested sprouty RTK signaling antagonist 2 (SPRY2) as a potential target of HOXA5 in colorectal cancer. SPRY2, a mammalian SPRY orthologue, is commonly inactivated in PCa and acts as a tumor suppressor [23, 24]. SPRY2 deficiency cooperates with the loss of PTEN or PP2A tumor suppressor activity to drive PCa initiation and androgen deprivation therapy resistance [24, 25]. Therefore, it would be worth analyzing whether SPRY2 acts as a downstream target of HOXA5 in PCa.

In this study, we aim to elucidate the function and mechanism of HOXA5 in PCa progression. HOXA5, which is identified as a novel target of TRAF7, has been demonstrated to bind directly to the SPRY2 promoter and subsequently regulate the MEK/ERK signaling pathway, thus to affect PCa proliferation, metastasis, and cancer stemness. To the best of our knowledge, our study explores the biological role of HOXA5 in PCa for the first time, which provides a novel perspective for targeted PCa therapy.

## Results

### HOXA5 is significantly downregulated in PCa cells and tissues

**Fig. 1:**
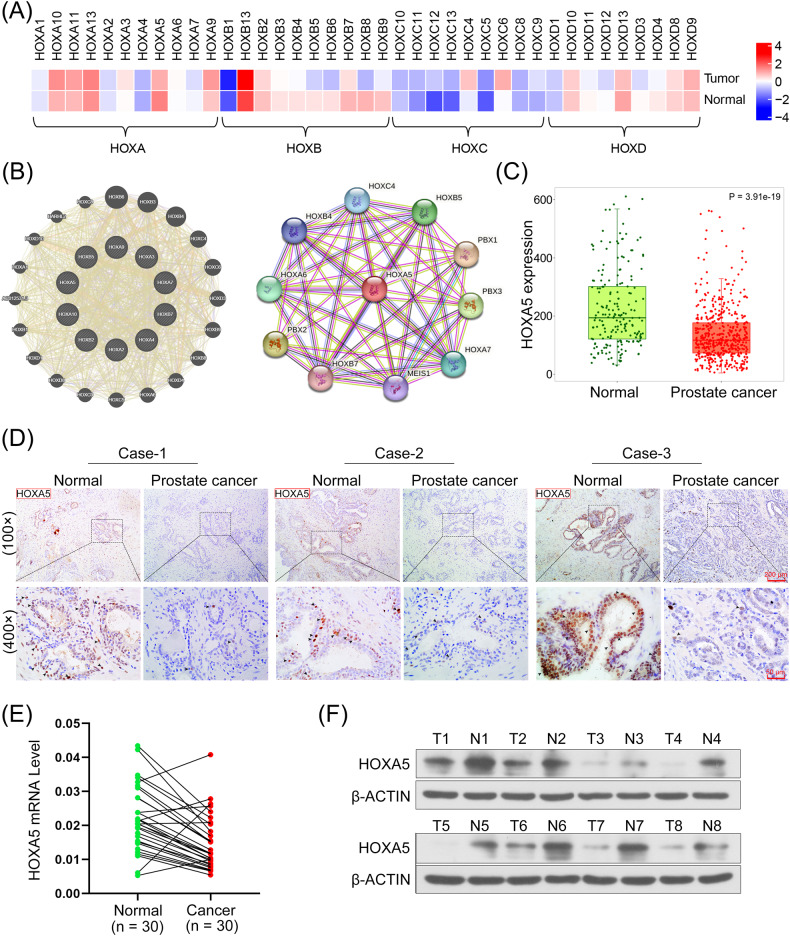
Homeobox A5 (HOXA5) levels are decreased in human prostate cancer (PCa) tissues. **A** The clustering heatmap for HOX gene expression profile between PCa tissues and the corresponding normal samples. **B** The gene-gene interaction network of HOX genes based on generated GeneMANIA and STRING. **C** Data about HOXA5 expression in PCa tissues and normal prostate samples were obtained from the The Cancer Genome Atlas (TCGA) dataset based on the TNMplot database. **D** Representative images of IHC staining of HOXA5 on paired normal and prostate cancer tissue samples. Images were taken at 100× and 400× magnifications. **E** HOXA5 mRNA level in paired PCa tissues and adjacent normal tissues. **F** Representative images of HOXA5 expression detected by Western Blot assay. *P < 0.05, **P < 0.01. Results were presented as mean ± SD.

HOX gene expression in the PCa and normal tissues were clustered using a heatmap analysis based on the GEPIA database (Fig. 1A). The probable gene interactions of the HOX family were plotted using the GeneMANIA and STRING databases, and the results indicated that the HOXs interacted closely with each other (Fig. 1B). HOXA5 expression in PCa tissues were analyzed with TNM-plot based on TCGA datasets. Our results indicated that HOXA5 was significantly downregulated in PCa tissues compared with that in normal samples (*P* < 0.0001; Fig. 1C). Concordantly, the IHC staining assay revealed that HOXA5 expression was lower in PCa tissue sections than in adjacent normal tissues (Fig. 1D). HOXA5 downregulation was further validated using qRT-PCR analysis of 30 paired PCa and adjacent normal tissues (Fig. 1E) and western blotting analysis (Fig. 1F). The expression of HOXA5 in PCa cell lines (DU145, 22RV1, PC-3, and VCaP) was also detected. Likewise, decreased HOXA5 levels were observed in PCa cell lines compared with those in the normal human prostate epithelial cells (RWPE-1) (Supplementary Fig. 1A). PC-3 and VCaP cells were selected for subsequent HOXA5 overexpression and downregulation.

To determine the relevance of HOXA5 in PCa progression, HOXA5 gain-and-loss-of-function assays were performed. PCa cells were infected with two lentiviral shRNAs specific for HOXA5 to downregulate HOXA5 expression and construct stable ectopic HOXA5-overexpressing cell lines for functional assays. The transfection efficiency was evaluated using western blotting (Supplementary Fig. 1B).

### HOXA5 reduces PCa cell viability and proliferation in vitro

**Fig. 2:**
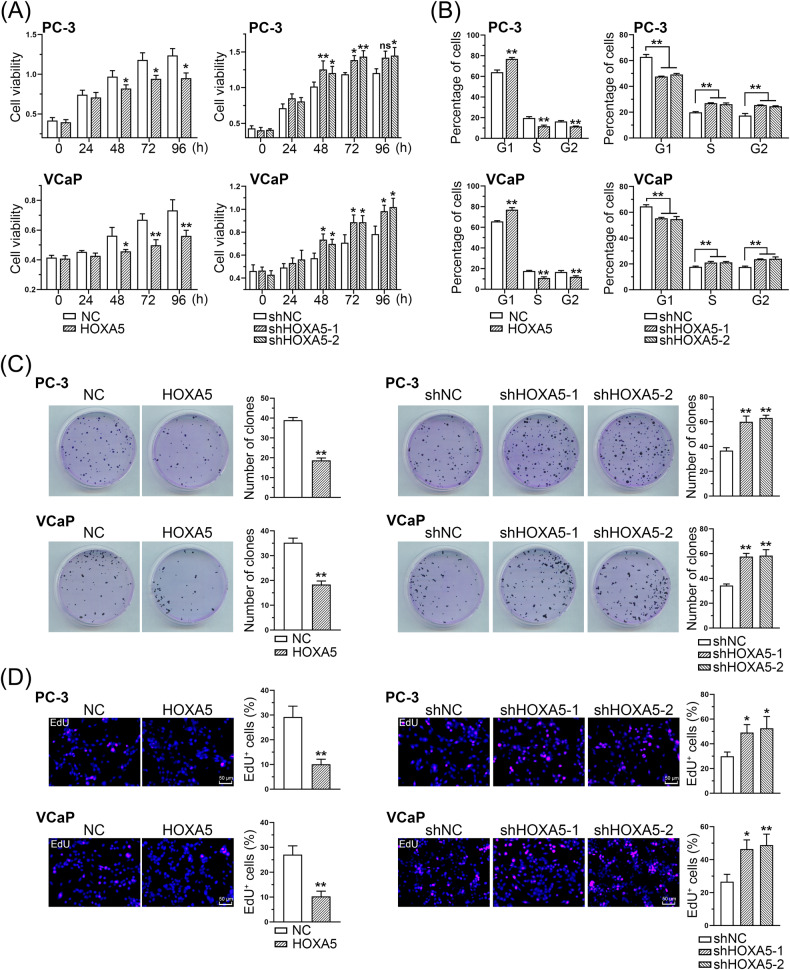
HOXA5 expression suppresses PCa cell growth in vitro. **A** PCa cell proliferation detected by CCK-8 assays of PCa cell lines with HOXA5 interfered at indicated time points. **B** Flow cytometry data for cell cycle distribution of indicated PCa cell lines. **C** Representative images of colony formation and corresponding quantitative results. **D** The proliferation was determined by EdU staining and corresponding quantification assay. *P < 0.05, **P < 0.01. Results were presented as mean ± SD.

Functional assays were performed to assess the effects of HOXA5 on PCa cell proliferation. The CCK-8 assay revealed that cell proliferation was significantly suppressed following HOXA5 overexpression, while HOXA5 knockdown increased the cells viability (Fig. 2A). Flow cytometry-based cell cycle analysis revealed that HOXA5 could regulate the cell cycle, showing an increase in the percentage of cells arrested in G1 phase with a concomitant decrease in S/G2 phase cells in HOXA5-overexpressing cells. In contrast, silencing HOXA5 resulted in the suppression of PCa cells in the G1 phase and the upregulation of cells in the S phase (Fig. 2B). Similarly, the colony formation assay revealed that ectopic HOXA5 expression reduced the number of PCa cell colonies, whereas inhibiting HOXA5 led to the opposite effect on the cells (Fig. 2C). EdU staining and quantification assays were performed to confirm the role of HOXA5 in PCa cell proliferation. A substantial reduction in the number of EdU-incorporated PCa cells was associated with HOXA5 overexpression, whereas the suppression of HOXA5 led to increased EdU-positive cells (Fig. 2D). Therefore, our data indicate that HOXA5 plays a significant role in both PCa tumorigenicity and progression.

### HOXA5 suppresses the migration, invasion, and epithelial-mesenchymal transition (EMT) of PCa cells in vitro

**Fig. 3:**
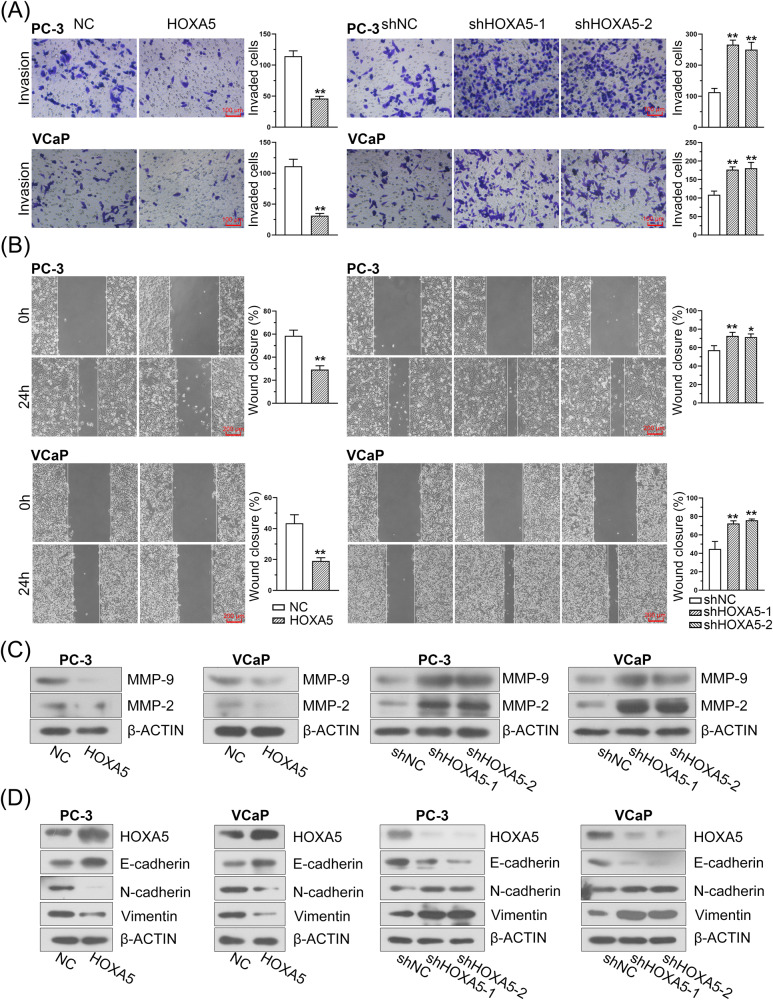
HOXA5 overexpression inhibits PCa cell migration, invasion, and EMT phenotype. **A**, **B** The effect of HOXA5 on cell migration and invasion evaluated cells by transwell and wound healing assays, and corresponding quantification for migrated and invaded cells. **C** The expression of MMP-2 and MMP-9 expression in PCa cells was detected by Western blot assay. **D** The expression of EMT-related markers (E-cadherin, N-cadherin, and vimentin) in PCa cells was detected by Western blot assay. *P < 0.05, **P < 0.01. Results were presented as mean ± SD.

To determine whether HOXA5 regulates the invasion and migration of PCa cells, transwell and would healing assays were performed. Our results showed that there were a steep fall in the capacity of PCa cells to migrate and invade in stable ectopic HOXA5-expressing cells (Fig. 3A, B). Concordantly, the invasion and migration of the cells were obviously elevated following HOXA5 inhibition. Enhanced invasiveness is closely linked to the preferential expression of matrix metalloproteinases (MMPs). As indicated in Fig. 3C, a robust reduction in the expression of MMP-2 and MMP-9 was observed in the HOXA5-overexpressing PCa cell lines, whereas a remarkable upregulation of these markers was found in the HOXA5-silenced cells. As EMT plays an indispensable role in PCa migration, invasion, and metastasis [26, 27], the expression of EMT-related markers was further investigated using western blotting. The abundance of the epithelial marker E-cadherin increased, whereas the levels of the mesenchymal-related markers N-cadherin and Vimentin decreased following HOXA5 overexpression. On the contrary, silencing HOXA5 led to increased N-cadherin and Vimentin expression and decreased E-cadherin expression (Fig. 3D), supporting the notion that HOXA5 knockdown contributes to the EMT phenotype in PCa.

### HOXA5 inhibits tumor self-renewal and is associated with stem cell-like properties in PCa

**Fig. 4:**
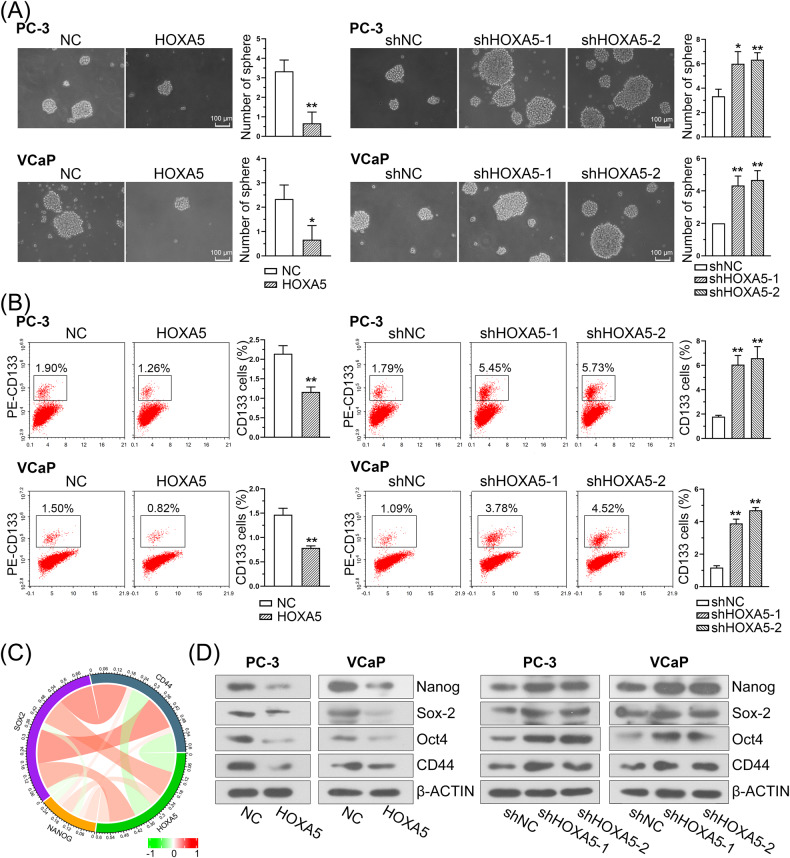
HOXA5 expression is correlated with stem-like properties in PCa. **A** Representative images of sphere formation and corresponding quantitative results. **B** Flow cytometry assay with CD133 in PCa cell lines and quantitative results. **C** Circos plot displaying the interconnectivity between HOXA5 and stemness-related markers. **D** The protein expression of cancer stem cell markers including *Nanog*, *Sox-2*, *Oct4*, and *CD44* in PCa cells. *P < 0.05, **P < 0.01. Results were presented as mean ± SD.

Since HOXA5 has been linked to the regulation of stem cells in normal tissue development [10], the effects of HOXA5 on the self-renewal ability of PCa cells were investigated. We observed HOXA5 overexpression significantly impaired tumor sphere formation of PCa cells, while HOXA5 knockdown facilitated more tumor spheres (Fig. 4A). In addition, flow cytometry assay revealed that the aberrant overexpression of HOXA5 led to decreased CD133+ subpopulations, whereas silencing HOXA5 led to a significant increase in the percentage of the CD133+ stem cell subpopulation (Fig. 4B).

To further investigate the correlation between HOXA5 and stemness-related genes, silico analysis was conducted using TCGA datasets. The results revealed a significant correlation between HOXA5 and CSC markers, including *Nanog*, *Sox-2*, and *CD44* (Fig. 4C). The expression of the CSC markers was then evaluated in PC-3 and VCaP cells. As expected, the HOXA5-overexpressing cells exhibited a reduced expression of these markers, and the HOXA5-silenced cells demonstrated upregulation in the expression of above markers (Fig. 4D).

### HOXA5 inhibits tumor growth and distant metastases in vivo

**Fig. 5:**
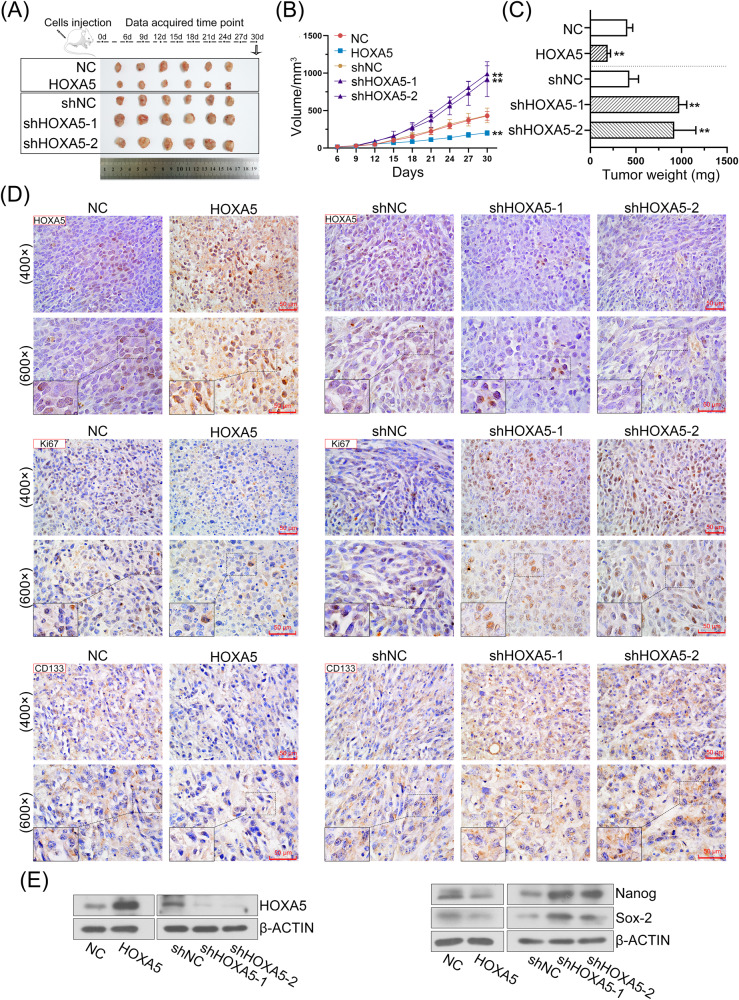
HOXA5 inhibits PCa growth in vivo. Subcutaneous tumor growth in mice inoculated with HOXA5-overexpressed or silenced PC-3 cells. **A** Representative pictures of xenografted tumors derived from PC-3 cells. **B** Tumor volume was measured every 3 days. **C** The average weight of tumor mass in each group was shown. **D** IHC analysis of HOXA5, Ki-67 and CD133 in sections of xenografts. Images were taken at 400× and 600× magnifications, respectively. **E** The expression of HOXA5 and CSC markers (*Nanog* and *Sox-2*) in tumor tissues was detected by Western blot assay. *P < 0.05, **P < 0.01. Results were presented as mean ± SD of six animals per group.

A subcutaneous xenograft model was established to investigate the role of HOXA5 in tumorigenesis in vivo. HOXA5 upregulation strongly attenuated the growth rate of xenograft tumors in mice. As a result, the weights of the tumors were lower in the group injected with HOXA5-overexpressed cells, whereas HOXA5 downregulation significantly promoted tumor growth (Fig. 5A–C). Meanwhile, HOXA5 expression in the tumor tissues was verified using IHC staining (Fig. 5D; upper panel) and western blotting (Fig. 5E; left panel). IHC staining for Ki67 and CD133 in paraffin-embedded tumor tissues was also performed. There was a significant reduction in Ki67-positive cells in the tumors xenografted with HOXA5-overexpressed PC-3 cells, but HOXA5 knockdown was accompanied by upregulated Ki67 (Fig. 5D; middle panel). These data indicated that HOXA5 upregulation inhibited tumor growth in vivo. To further determine whether HOXA5 modified stemness phenotype to control PCa progression, we determined stemness-related gene expression expression in PCa tissues. IHC staining showed that the expression of CD133 was significantly decreased in the tumors xenografted with HOXA5 overexpressed PCa cells as compared to control, and increased following HOXA5 knockdown (Fig. 5D; lower panel). Consistent with the in vitro results, HOXA5 overexpression also decreased the protein levels of CSC markers, including *Nanog* and *Sox-2* (Fig. 5E; right panel).

**Fig. 6:**
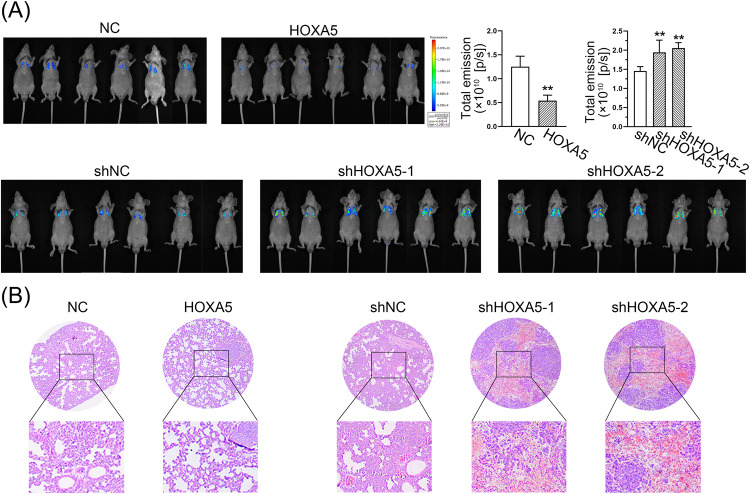
HOXA5 impairs PCa distant metastasis in vivo. **A** PC-3 cells with HOXA5 overexpression or silencing were intracardially injected into mice and the metastatic colonization was taken four weeks after injection. Bioluminescence intensity was shown as total emission (photons/second) and quantified. **B** H&E staining of the lungs from mice to detect lung metastases. *P < 0.05, **P < 0.01. Results were presented as mean ± SD of six animals per group.

Furthermore, the impact of HOXA5 on the metastatic potential of PCa was further investigated by intracardially injecting HOXA5-knockdown or -overexpressed PCa cells into mice. Bioluminescent images were captured at the end of week 4 post tumor cells inoculation. As shown in Fig. 6A, HOXA5-overexpressed animals displayed substantially less metastatic signal, read as radiance (photons/second), and loss of HOXA5 expression aggravated the pulmonary metastases. Consistently, H&E-stained slides indicated that tumors formed from HOXA5-expressing cells exhibited fewer metastatic nodes (Fig. 6B). Above findings suggested that activation of HOXA5 is sufficient to inhibit the PCa growth and metastasis in vivo.

### HOXA5 negatively regulates the activation of the MEK/ERK pathway in PCa cells

**Fig. 7:**
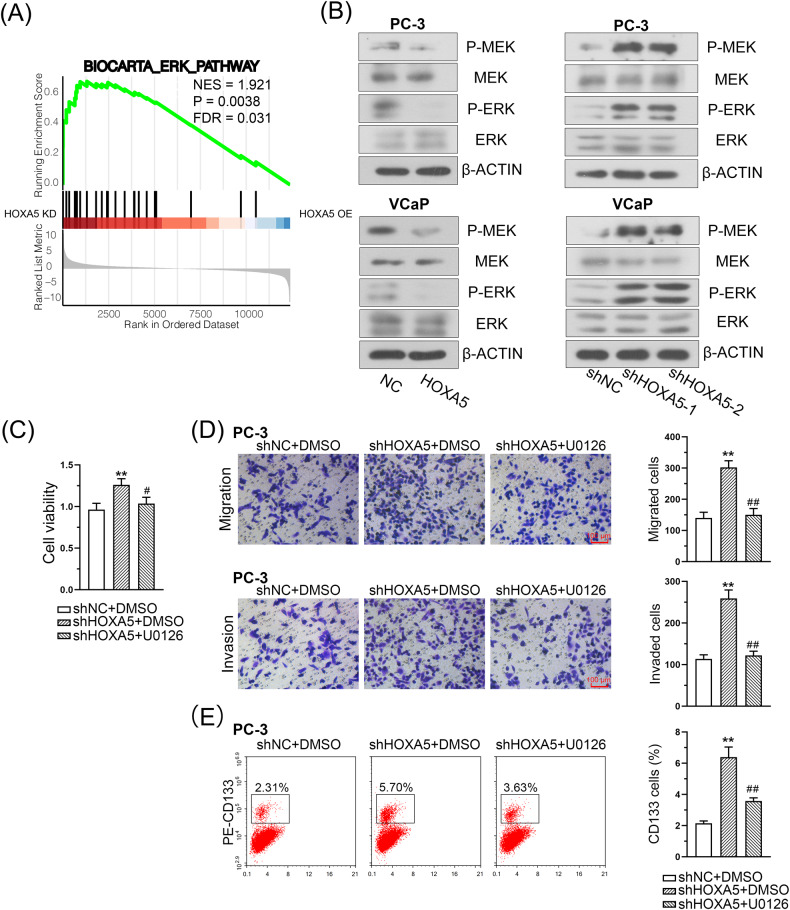
HOXA5 suppresses the MEK/ERK signaling pathway in PCa cells. **A** GSEA analysis identified a significant enrichment of MEK/ERK signaling in HOXA5 silenced cells. **B** The expression of phospho-MEK, phospho-ERK, and total MEK and ERK in PCa cells. The transfected PC-3 cells were treated with 10 µM U0126 (a specific MEK/ERK inhibitor). **C** The viability of PC-3 cells was determined by a CCK-8 assay. **D** Representative images of migration and invasion and corresponding quantitative results. **E** Flow cytometry assay with CD133 in PC-3 cells and quantitative results. *P < 0.05, **P < 0.01. Results were presented as mean ± SD of three biological replicates.

Given that HOXA5 is closely associated with PCa cell growth and metastasis, we investigated the mechanisms by which HOXA5 regulates PCa progression. The GSEA indicated the significant enrichment of MEK/ERK signaling was associated with knockdown HOXA5 (Fig. 7A). Next, our results confirmed that the MEK/ERK pathway was activated in PCa cells following HOXA5 knockdown, as evidenced by the increased expression of phosphorylated MEK (P-MEK) and P-ERK. Additionally, overexpressed HOXA5 was associated with lower levels of P-ERK and P-MEK (Fig. 7B). The oncogenic role of the MEK/ERK pathway has been well documented in various cancers [28, 29]. To validate the effects of HOXA5 on MEK/ERK signaling, PC-3 cells were treated with U0126, a specific MEK/ERK inhibitor. This stimulation reversed the PC-3 cell proliferation that resulted from HOXA5 knockdown. The U0126 treatment also partially suppressed cell invasion, migration, and the percentage of CD133+ stem cell subpopulations induced by HOXA5 inhibition (Fig. 7C–E). Collectively, these results support that HOXA5 negatively regulates PCa cell proliferation and metastasis through the MEK/ERK pathway.

### TRAF7 promotes PCa cell proliferation and invasion through the ubiquitin-mediated degradation of HOXA5

**Fig. 8:**
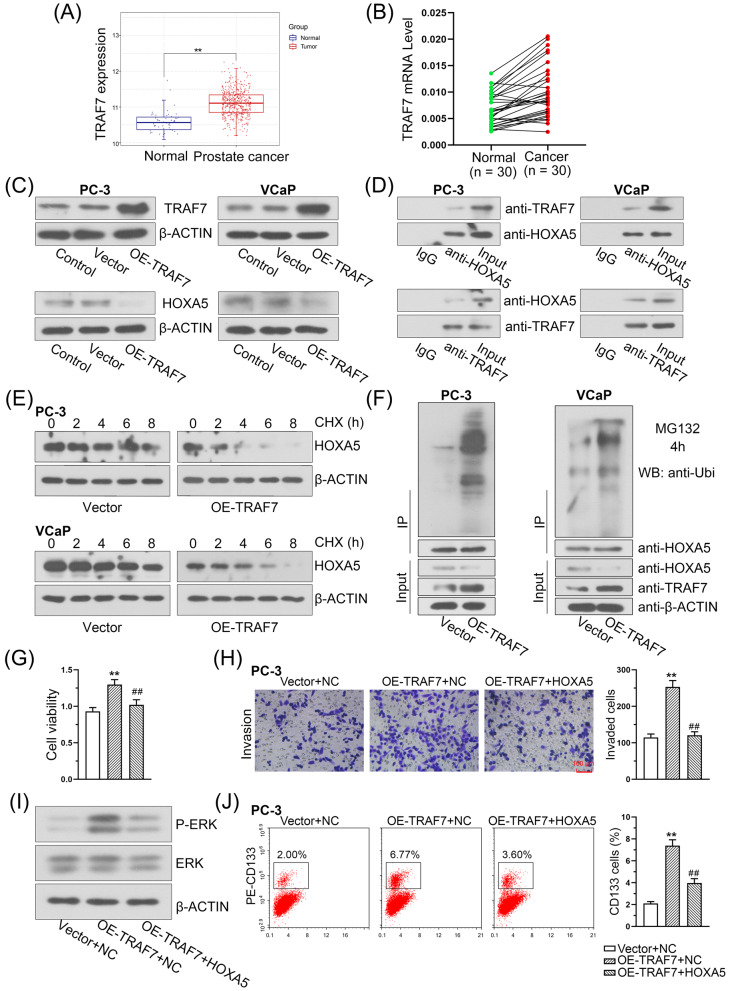
TRAF7 negatively regulates the expression of HOXA5 through ubiquitin-mediated degradation. **A** TRAF7 expression in PCa tissues and normal tissues were obtained from the Gene Set Cancer analysis database. **B** TRAF7 mRNA level in PCa tissues and paired adjacent normal tissues. **C** Western blot analysis of TRAF7 and HOXA5 expression in PCa cells with TRAF7 overexpression. **D** Endogenous co-immunoprecipitation was performed to determine the interaction between TRAF7 and HOXA5 in PCa cells. TRAF7 was immunoprecipitated with a HOXA5 antibody and vice versa. **E** HOXA5 protein levels were determined after incubation with cycloheximide (CHX) for the indicated time periods in PC-3 and VCaP cells following TRAF7 overexpression. **F** PC-3 and VCaP cells were incubated with MG132 for 4 h. Cell lysates were immunoprecipitated with HOXA5 antibody and then immunoblotted with ubiquitin antibody. The increased cell proliferation (**G**), cell invasion (**H**), phospho-ERK expression (**I**), and percentage of CD133+ stem cell subpopulation (**J**) caused by TRAF7 overexpression were significantly inhibited by the ectopic HOXA5 expression. *P < 0.05, **P < 0.01. ^#^*P* < 0.05, ^##^*P* < 0.01. Results were presented as mean ± SD.

Here, we found that TRAF7 expression was upregulated in PCa samples compared with that in normal control samples based on TCGA dataset (Fig. 8A). In addition, the overexpression of TRAF7 in PCa tissues was also verified in 30-paired PCa and adjacent normal tissues (Fig. 8B). Western blotting was further performed to explore the relationship between TRAF7 and HOXA5 in PCa cell lines. The results showed that TRAF7 overexpression significantly reduced HOXA5 levels in PC-3 and VCaP cells (Fig. 8C). TRAF7 was reported to exert multiple effects during carcinogenesis by ubiquitinating its targets [19, 30]. Along these lines, we hypothesized that HOXA5 might be a potential target of TRAF7. The direct interaction between TRAF7 and HOXA5 was investigated using a Co-IP assay, which confirmed that TRAF7 readily co-immunoprecipitated with HOXA5 and vice versa (Fig. 8D). To further investigate whether TRAF7 modulates HOXA5 expression through ubiquitin-mediated degradation, we assessed the stability of the HOXA5 protein in PC-3 and VCaP cells after blocking its synthesis with cycloheximide (CHX). As a result, TRAF7 overexpression substantially accelerated the degradation of HOXA5 (Fig. 8E). Notably, the immunoprecipitation assays indicated that, in the presence of MG132 (a proteasome inhibitor), TRAF7 overexpression increased the ubiquitination of HOXA5 (Fig. 8F). Collectively, these findings suggest that the regulation of HOXA5 by TRAF7 is proteasome-dependent and TRAF7 contributes to the destabilization of HOXA5. The results of functional assay demonstrated that TRAF7 upregulation not only promoted PCa cell proliferation and invasion (Fig. 8G, H), but also positively correlated with P-ERK (Fig. 8I), as well as CSC markers (Fig. 8J). Moreover, these effects can be reversed by restoring HOXA5 expression (Fig. 8G–J).

### HOXA5 exerts its function in PCa by transcriptionally activating SPRY2

**Fig. 9:**
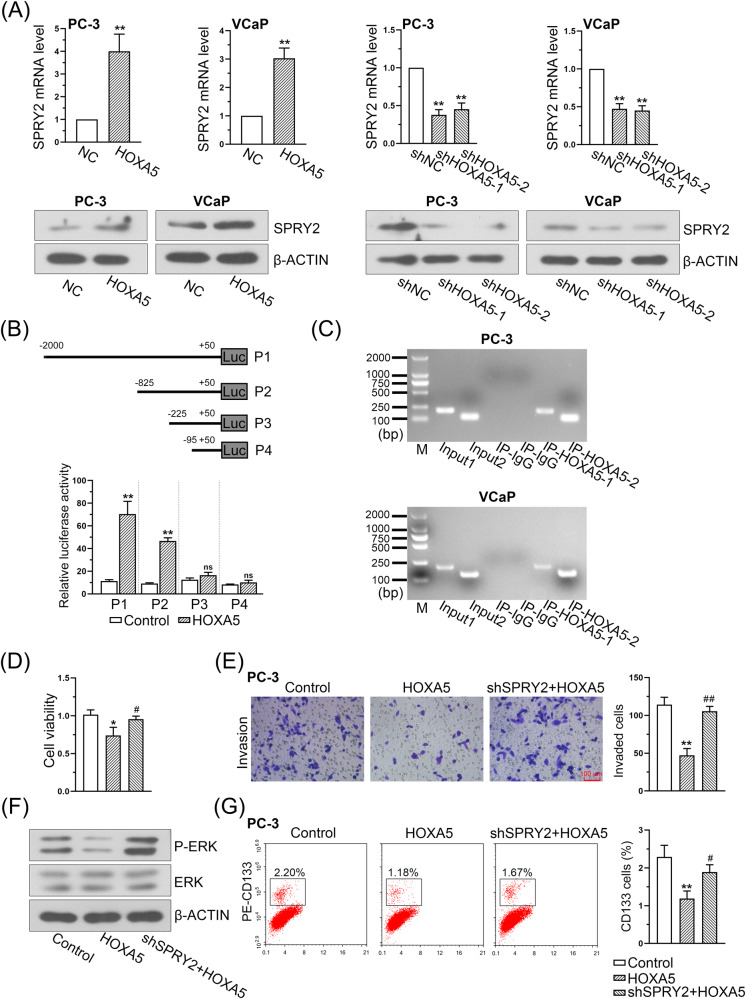
HOXA5 promotes SPRY2 transcription by binding to the promoter of SPRY2. **A** SPRY2 mRNA and protein levels in HOXA5-overexpressed and HOXA5-silenced PCa cells. **B** SPRY2 promoter reporters were co-transfected with HOXA5 overexpressed plasmid into 293T cells. The SPRY2 promoter activity was determined by a dual luciferase assay kit. **C** ChIP assay showed the binding of HOXA5 to the promoter of SPRY2 in PCa cells. The decreased cell proliferation (**D**), cell invasion (**E**), phospho-ERK expression (**F**), and percentage of CD133+ stem cell subpopulation (**G**) caused by HOXA5 overexpression were partially rescued by the transfection with SPRY2-shRNA. ^*^P < 0.05, ^**^P < 0.01. ^#^*P* < 0.05, ^##^*P* < 0.01. Results were presented as mean ± SD.

Real-time PCR and western blotting revealed that the upregulation of HOXA5 led to increased SPRY2 expression, whereas HOXA5 knockdown significantly inhibited the levels of SPRY2 in both PC-3 and VCaP cells (Fig. 9A). Next, the conserved binding site of HOXA5 was further determined from the JASPAR database, and its putative binding sites in the SPRY2 promoter were predicted. Various lengths of SPRY2-flanking regions were cloned and transiently transfected into HOXA5-overexpressing PCa cells to determine promoter activity using a dual-luciferase assay. The luciferase activity of SPRY2 was significantly increased (Fig. 9B). Furthermore, the ChIP assay confirmed that chromatin fragments corresponding to putative HOXA5 binding sites were specifically present in anti-HOXA5 immunoprecipitates from PC-3 and VCaP cells. The anti-HOXA5 antibody pulled down higher amounts of the SPRY2 promoter segments than that of the control (Fig. 9C). These data confirm that SPRY2 is the direct downstream target of HOXA5 and can be regulated by HOXA5 in PCa.

We further examined whether silencing SPRY2 could compromise the inhibitory effect of HOXA5 in PCa. A SPRY2-knockdown plasmid vector was transfected into HOXA5-overexpressing PC-3 cells. The intervention efficiency was examined using western blotting (Supplementary Fig. 1C). As expected, SPRY2 knockdown significantly recovered the decreased proliferation (Fig. 9D) and invasion of the cells (Fig. 9E), and increased P-ERK expression (Fig. 9F) and CD133+ stem cell subpopulations (Fig. 9G) in PCa cells resulting from HOXA5 overexpression.

## Discussion

Increasing evidence has shown the involvement of HOXA5 in cancer cell proliferation, metastasis, and other processes [10, 31]. Both the upregulation and downregulation of HOXA5 have been reported in a variety of malignant tumors, suggesting its role as a tumor suppressor and promoter [32, 33]. In the present study, we first demonstrated that HOXA5 is downregulated at the mRNA and protein levels in PCa based on public databases and our patient cohort, indicating that HOXA5 might be involved in PCa progression. Further functional assays revealed that HOXA5 knockdown promoted PCa cell growth, aggressiveness, and EMT phenotype. Meanwhile, our findings from subcutaneous and intracardiac xenograft tumor models indicated that HOXA5 upregulation was sufficient to reduce tumor sizes and distant metastases, thus supporting the tumor-suppressive role of HOXA5 in PCa progression.

PCa stemness contributes to lineage plasticity, poor responses to androgen deprivation therapy, and the acquisition of aggressive phenotypes [34, 35]. Since HOXA5 has been considered a stem cell-related gene [36], we further investigated whether HOXA5 was involved in driving PCa stemness. Intriguingly, our results confirmed that HOXA5 functioned as a key regulator of PCa stemness traits. HOXA5 downregulation could not only promote the self-renewal capacity of PCa cells, but increased the expression of CSC pluripotency markers. Consistently, in vivo assays further indicated that the expression of stemness markers was significantly increased following HOXA5 knockdown, suggesting that HOXA5 inhibition could attenuate the cancer stemness of PCa cells. Notably, a recent study using a breast cancer model demonstrated that the loss of HOXA5 induced cellular transformation from an epithelial to a basal phenotype [37]. Given the importance of cellular plasticity and stemness in the development of aggressive neuroendocrine PCa [6], it will be interesting to investigate whether HOXA5 also regulates neuroendocrine PCa transformation in future study.

To elucidate the underlying mechanisms of HOXA5 in PCa progression, we conducted a GSEA and identified significant enrichment in MEK/ERK signaling in HOXA5-silenced cells. Besides, proteins interacting with *homo* HOXA5 were identified from the Hitpredict database, and the following GO annotation indicated that these proteins were involved in ERK cascades (Supplementary Table 3). As evidenced by western blotting, HOXA5 knockdown enhanced MEK/ERK signaling activation, whereas HOXA5 upregulation resulted in the opposite effect. Moreover, using a MEK-specific inhibitor, U0126, could suppress the aggressive phenotypes and CSC properties of PCa cells conferred by HOXA5 knockdown. Recently, increasing emphasis has been placed on developing MEK/ERK inhibitors for targeted therapy [28, 38]; however, patients respond only partially to single-drug treatments, and MEK/ERK inhibitors would be best if given in combination with other drugs, instead of as a single agent. Our results suggest that it may be therapeutically relevant to consider methods for enhancing HOXA5 expression in PCa, in combination with MEK/ERK inhibitors.

Understanding the regulation of HOXA5 in PCa is as crucial as identifying its upstream regulators and specific downstream targets. By analyzing proteins interacting with HOXA5, we identified TRAF7 as a candidate, which is also annotated in the ERK cascades. The emerging role of TRAF7 in the occurrence and progression of human cancers has been well-documented [17, 19, 39]. It has been reported that TRAF7 enhanced ubiquitin-degradation of p53 and KLF47 in cancer, both of which play a cancer-suppressing role in prostate cancer [40, 41]. In this study, our findings confirmed that HOXA5 is a direct target of the ubiquitin ligase TRAF7 and that TRAF7 facilitates the protein degradation of HOXA5 through its ubiquitin ligase activity, which leads to the enhanced aggressive and metastatic phenotypes of PCa. These data together strengthen the notion that HOXA5 is targeted by TRAF7 and participates in the regulation of PCa. We are aware of that, aside from TRAF7, HOXA5 may also be regulated by other interactors enriched in the ERK pathway. Another limitation of our study lies in the origin of TRAF7 upregulation and whether it interacts with multiple signal transduction pathways to promote PCa progression remains obscure, which needs to be investigated in future studies.

In our current study, the downstream target genes of HOXA5 were predicted, by which SPRY2 was identified as a candidate target gene of HOXA5. The direct interaction between HOXA5 and SPRY2 was further confirmed using luciferase and ChIP assays. Previous studies reported that SPRY2 was frequently inactivated in PCa and its deficiency led to the occurrence of castration-resistant PCa [25, 42]. More importantly, it has been shown that SPRY2 may further function as a negative-feedback regulator of MEK/ERK signaling and exert its inhibitory role in tumor progression [43, 44]. In concert with those results, our study verified that HOXA5 could regulate the SPRY2-mediated MEK/ERK pathway. Therefore, our data suggest that HOXA5 transcriptionally activates SPRY2 and subsequently inhibits the activation of MEK/ERK signaling, thereby suppressing the malignant properties of PCa.

Taken together, our study demonstrates that HOXA5 is downregulated in PCa, and its levels are significantly associated with PCa progression and metastasis. Moreover, the loss of HOXA5 could promote PCa malignancy, EMT phenotype and drive cancer stemness through a previously-unreported TRAF7/HOXA5/SPRY2–MEK/ERK signaling axis. These findings provide a foundation for understanding the mechanisms underlying PCa progression and shed light on potential novel therapeutic approaches for PCa.

## Materials and methods

### Alterations in the genes of the HOX family

HOX family gene expression data were collected from The Cancer Genome Atlas database (TCGA, https://www.cancer.gov/tcga). The expression profiles of the HOX genes were illustrated using the R package heatmap. Gene networks were constructed using the GeneMANIA database (http://genemania.org/) to analyze the gene interaction network [45]. Interactions between the HOX genes were further explored using the STRING database (https://string-preview.org/). HOXA5 expression values were downloaded from the TCGA database, which included information from 498 PCa samples and 204 normal prostatic tissues. Hitpredict database (http://www.hitpredict.org/) was utilized to identify proteins that potentially interact with HOXA5. Functional enrichment analysis was performed based on the Gene Ontology (GO) resource (http://geneontology.org/).

### Human tissue specimens

Thirty freshly-frozen human PCa samples and adjacent prostate tissues were harvested from patients at Shengjing Hospital of China Medical University. Paraffinized samples were also collected for the immunohistochemical (IHC) staining of HOXA5. Two experienced urological pathologists evaluated each section. The clinicopathological characteristics of all the patients are summarized in Supplementary Table 1. All experiments were approved by the Institutional Research Ethics Committee of Shengjing Hospital of China Medical University (2021PS252K). Written informed consent was obtained from all the patients.

### IHC assay

The paraffin-embedded sections were dewaxed and rehydrated. Following incubation in H_2_O_2_, the sections were blocked with 5% normal goat serum and hybridized with primary antibodies against HOXA5 (Bs-5713R, Bioss, China), Ki-67 (AF0198, Affinity, China), and CD133 (AF5120, Affinity, China) at 4 °C overnight. The sections were then co-incubated with horseradish peroxidase-conjugated secondary antibodies (IgG, #31460, Thermo Fisher Scientific, China) for 1 h at 37 °C. Specimens were detected using a DAB solution in the dark, counterstained with hematoxylin, and mounted. Images were obtained using an optical microscope (Olympus, Tokyo, Japan).

### Cell line culture and cell transfection

The immortalized benign prostate cell line RWPE-1 and PCa cell lines DU145, 22RV1, and PC-3 were obtained from iCell Bioscience (China). VCap cells were acquired from Procell (China). The RWPE-1 cells were grown in specific media (iCell-h286-001b, iCell Bioscience, China); DU145 cells were grown in MEM (41500, Solarbio, China); 22RV1 cells were maintained in RPMI-1640 medium (31800, Solarbio, China); PC-3 cells were cultivated in F12K medium (PM150910, Procell, China); and VCap cells were grown in Dulbecco’s Modified Eagle Medium (DMEM; G4510, Servicebio, China). All cell lines were cultured at 37 °C with 5% CO_2_ and free from mycoplasma contamination. The PC-3 and VCap cells were used for transfection and then treated with 10 µM U0126 inhibitors (S81225, Shanghai yuanye Bio-Technology, China) for 48 h. DMSO was used as the control. To determine the protein half-life, the transfected cells were treated with the protein synthesis inhibitor cycloheximide (CHX, 100 mg/ml, C112766, Aladdin, China) for 0, 2, 4, 6, and 8 h. The proteins were then extracted, and their expression was assessed using western blot assays.

Lentiviruses expressing HOXA5 or containing empty vectors were transfected into the PCa cell lines. Additionally, lentiviruses carrying short hairpin RNA (shRNA) against HOXA5 (shHOXA5-1 and shHOXA5-2) and or negative control shRNA (shNC) were constructed. All vectors were transfected into PC-3 and VCap cells using a Lipofectamine 3000 kit (L3000015, Invitrogen, USA). Stable cell lines were selected using puromycin after infection.

### Quantitative real-time PCR (qRT-PCR)

Total RNA was extracted from the PCa tissues and cells using TRIzol reagent (RP1001, BioTeke, China). cDNA was prepared using the SYBR Green PCR detection system (Bioneer, Korea). The target mRNA expression was calculated relative to β-actin expression using the 2^-ΔΔCt^ method. The sequences of the primers were as follows: homo *HOXA5* sense, 5′-TTCAACCGTTACCTGACCCG-3′, and anti-sense, 5′-CGGCCATGCTCATGCTTT-3′; homo *SPRY2* sense, 5′-ACTCGCAGGTCCATTCTTC-3′, and anti-sense, 5′-TTCCTTGCTCAGTGGCTTA-3′.

### Western blot assay

The PCa tissues and cells were lysed in RIPA buffer (P0013B, Beyotime, China) containing PMSF (ST506, Beyotime, China) to extract the total protein. Protein concentrations were determined using a BCA Assay Kit (P0009, Beyotime, China). The proteins were fractionated using SDS-PAGE and electroblotted onto PVDF membranes (LC2005, Thermo Fisher Scientific, China). The membranes were cultured in 5% bovine serum albumin and hybridized with primary antibodies overnight at 4 °C. The blots were then incubated with horseradish peroxidase-conjugated secondary antibodies for 40 min. The immune complexes were then detected using ECL reagents. All of the antibodies used are listed in Supplementary Table 2.

### Cancer stem cell sphere formation assay

The PCa cells were placed into Ultra-Low Attachment Surface 96-well plates at a density of 200 cells/well. The cells were cultured in DMEM/F12 (BL305A, Biosharp, China) containing 20 ng/mL of epidermal growth factor (EGF, 10605-HNAE, Sino Biological Inc., China), 10 ng/mL of basic fibroblast growth factor (bFGF, 10014-HNAE, Sino Biological Inc., China), and 2% B27 supplement (17504044, Thermo Fisher Scientific, USA). The cells were incubated for 2 weeks, and tumor sphere formation was assessed using a light microscope (Olympus, Tokyo, Japan). The tumorsphere numbers (diameter > 50 µm) were then counted.

### Flow cytometry

The PCa cells were harvested following transfection and treatment. Approximately 10^6^ cells were incubated with CD133 antibodies (0.5 µg) for 20 min at 4 °C. CD133 staining was performed using a CD133 (Prominin-1) Monoclonal Antibody ((EMK08)-FITC eBioscience, 11-1339-41, Thermo Fisher Scientific, USA). The cells were then resuspended in 500 µL of binding buffer and analyzed using a flow cytometer (Aceabio, USA).

### Cell viability assay

For the cell proliferation assay, PCa cells were seeded in 96-well plates at a density of 4 × 10^3^ cells/well. Following transfection and treatment, cell viability was assessed using a cell counting kit-8 kit (CCK-8; C0037, Beyotime, China) after 24, 48, 72, and 96 h. Relative cell viability was evaluated using a microplate (E0226, Beyotime, China) reader at a wavelength of 450 nm.

### Cell cycle assay

The cell cycle was assessed using a cell cycle detection kit (C1052, Beyotime, China). In brief, transfected and treated PCa cells (5 × 10^5^) were fixed in 70% ethanol for 12 h and then stained with propidium iodide and RNase A in the dark for 30 min. Cell cycle distribution was analyzed using a NovoCyte flow cytometer (ACEA, USA), and the percentage of cells in different phases was calculated.

### Colony formation assay

After transfection, approximately 300 cells/well were seeded onto plates and cultured to form colonies. After 14 days of incubation, the colonies became visible, and the culture medium was replaced. The cells were then fixed and stained with Wright-Giemsa stain solution (D011-1-2, Nanjing Jiancheng Bioengineering Institute, China). The number of PCa cell colonies formed was counted using an inverted microscope (Olympus, Japan).

### EdU proliferation assay

Cell proliferation was assessed by EdU staining using the Click-iT EdU kit (KGA335, KeyGEN, China). In brief, transfected PCa cells were cultured in 96-well plates and incubated with EdU solution (10 µM) for 2 h. The cells were fixed using 4% paraformaldehyde and eluted using 0.5% TritonX-100 for 20 min, followed by Click-iT and DAPI staining. Images of the EdU-labeled cells were acquired using a fluorescence microscope (Olympus, Tokyo, Japan) and EdU-positive cells were counted.

### Transwell assays

Cell migration and invasion were monitored using transwell assays. The chambers (5 µm pore size, 14131 LABSELECT, China) were pre-coated with Matrigel (Corning, USA) for the transwell invasion assay. Approximately 5 × 10^4^ cells were suspended in serum-free culture media in transwell inserts. The basolateral chambers were loaded with growth media supplemented with 10% fetal bovine serum. The cells were incubated at 37 °C for 24 h. Invasive cells were stained with crystal violet, photographed, and counted. For the transwell migration assay, the PCa cells were plated at a density of 5 × 10^3^ cells into the upper chamber of 24-well plate transwell inserts and allowed to migrate for 24 h. Media containing 10% fetal bovine serum in the lower chamber served as the chemoattractant. Migrating cells stained with crystal violet were counted under a microscope in five random fields.

### Wound healing assay

PCa cells were cultured until spread over the plate and incubated in a serum-free medium with mitomycin C (M0503, Sigma, USA) for 1 h. Wounds were created by a 200 µL pipette tip, and cells were washed with PBS. The images were captured with a light microscope (Olympus, Tokyo, Japan) from the same field at 0 and 24 h, and the wound closure was measured.

### Growth of xenograft tumors in mice and pulmonary metastasis models

All animal experiments were approved by the Institutional Animal Care and Use Committee of Shengjing Hospital of China Medical University (No. 2021PS353K) and followed the ARRIVE Guidelines for Reporting Animal Research. Six-week-old male nude mice (Beijing HFK Bioscience Co., Ltd.) were housed under standard conditions to establish tumor xenograft models. The sample size was estimated by power analysis and our experience. No animals were excluded.

Mice were divided into five groups (*n* = 6/group): shNC, shHOXA5-1, shHOXA5-2, NC, and HOXA5, based on the random number table approach. PC-3/vector cells, HOXA5-overexpression cells (1 × 10^6^), or PC-3 cells transfected with shNC or shHOXA5 were injected into the right armpits of the mice. Tumor growth was monitored every 3 days using a digital caliper (V = length × width^2^/2). After 30 days, the mice were euthanized according to the protocol of the Animal Research Committee. Tumor tissues were dissected, weighed, and fixed in 4% paraformaldehyde for further analysis.

For metastasis detection, PCa cells (1 × 10^5^ cells) infected with recombinant pHIV-Luc-ZsGreen lentivirus (#39196, Addgene) with overexpressed or silenced HOXA5 were injected intracardially into the left ventricles of the mice. After 4 weeks, the mice were injected with 5 mg/kg of d-luciferin. In vivo fluorescence imaging was performed using a small animal living imager after 5 min. Only mice with a detectable bioluminescence were used. Quantitative bioluminescence imaging signal after the d-luciferin injection was measured. The mice were then euthanized, and their lung tissues were harvested and fixed with 4% paraformaldehyde for subsequent experiments.

### Hematoxylin and eosin (H&E) staining

The paraffin-embedded lung specimens were deparaffinized, rehydrated, and stained with hematoxylin to observe lung metastasis of the PCa cells. Slides were mounted, and images were captured using a microscope (40× magnification, Olympus, Japan).

### Gene set enrichment analysis (GSEA)

A GSEA was performed using the ClusterProfiler package in R to identify the gene sets associated with HOXA5 expression. The curated gene sets from the Molecular Signatures Database (MSigDB v2023.1.Hs) served as background gene sets for analysis. The analysis involved using the fold changes of the genes between the HOXA5-overexpressed (HOXA5 OE) and HOXA5-knockdown (HOXA5 KD) cells as the sorting metric. The parameters during the operation were as follows: exponent = 1, nPerm = 1000, minGSSize = 10, maxGSSize = 500, pvalueCutoff = 0.25, pAdjustMethod = “BH,” verbose = TRUE, seed = TRUE, by = “fgsea.”

### Co-immunoprecipitation (Co-IP) and ubiquitination assays

The interaction between TRAF7 and HOXA5 was determined using a Co-IP assay. The transfected cells were treated with MG132 (10 µg/mL, M126521, Aladdin, China) for 4 h and harvested to detect HOXA5 ubiquitination. Total protein was extracted using western blotting and IP lysis buffer (P0013, Beyotime, China). The protein lysates were incubated with IP-indicated antibodies against HOXA5 (sc-365784, Santa Cruz Biotechnology, China), TRAF7 (11780-1-AP, Proteintech, China), ubiquitin (10201-2-AP, Proteintech, China), or negative control IgG at 4 °C overnight. The Co-IP assay was performed using the Pierce™ Co-Immunoprecipitation Kit (#26149, USA) according to the manufacturer’s instructions. The immunoprecipitates were analyzed using SDS-PAGE and western blotting.

### Dual-luciferase reporter assay

For the dual-luciferase reporter assay, 293T cells were obtained from iCell Bioscience (China) and cultured in DMEM supplemented with 10% fetal bovine serum. Putative binding sites between HOXA5 and the SPRY2 promoter were determined using JASPAR (http://jaspar.genereg.net/). The SPRY2 promoter was co-transfected with the HOXA5 plasmid into PCa cells. After 48 h, the cells were harvested, and the luciferase activity of SPRY2 was assessed using the Dual-Luciferase Reporter Assay System (KGAF040, KeyGEN, China) according to the manufacturer’s protocol. The *Renilla* luciferase expression plasmid was used as an internal control.

### Chromatin immunoprecipitation (ChIP)

The binding between HOXA5 and the SPRY2 promoter was determined using a ChIP Assay Kit (WLA106a, Wanleibio, China). In brief, PCa cells were crosslinked with 1% formaldehyde. After termination with 1 M glycine, the cells were sonicated to fragmented sizes between 200–1000 bp. After centrifugation for 20 min at 12,000 rpm and 4 °C, the supernatant containing sheared chromatin was collected and incubated with the HOXA5 or IgG antibodies for immunoprecipitation. Protein A beads were added to collect antibody–histone complexes. Protein complexes have been extensively used to reverse histone–DNA crosslinks. The DNA was extracted using a DNA Gel Extraction Kit (WLA052a, Wanleibio, China). The immunoprecipitated chromatin was examined using Taq PCR with SPRY2-specific primers.

### Statistical analysis

All experiments were carried out three times and the quantitative data are presented as the mean ± standard deviation (SD). The normal distribution and the homogeneity of variance were tested first. For normally distributed data, two-tailed Student’s *t* test and one-way or two-way analyses of variance were adopted, followed by Tukey’s post hoc test for the latter two methods. A non-parametric Mann–Whitney test was applied for nonnormally distributed data. Statistical analyses were performed using GraphPad Prism (version 6.0, GraphPad Software, Inc.). ^*^P < 0.05 and ^**^P < 0.01 were considered statistically significant.

### Supplementary information

Supplementary Figure 1

Supplementary figure legend

Supplementary Table 1

Supplementary Table 2

Supplementary Table 3

Supplementary material for original WB

## Data Availability

All data and material are available from the corresponding author upon reasonable request.
