# Supplementary figures and images for: TRAF7-targeted HOXA5 acts as a tumor suppressor in prostate cancer progression and stemness via transcriptionally activating SPRY2 and regulating MEK/ERK signaling
Source: Cell Death Discov. 2023 Oct 16;9:378. doi: 10.1038/s41420-023-01675-9 (PMC10579307; doi:10.1038/s41420-023-01675-9)

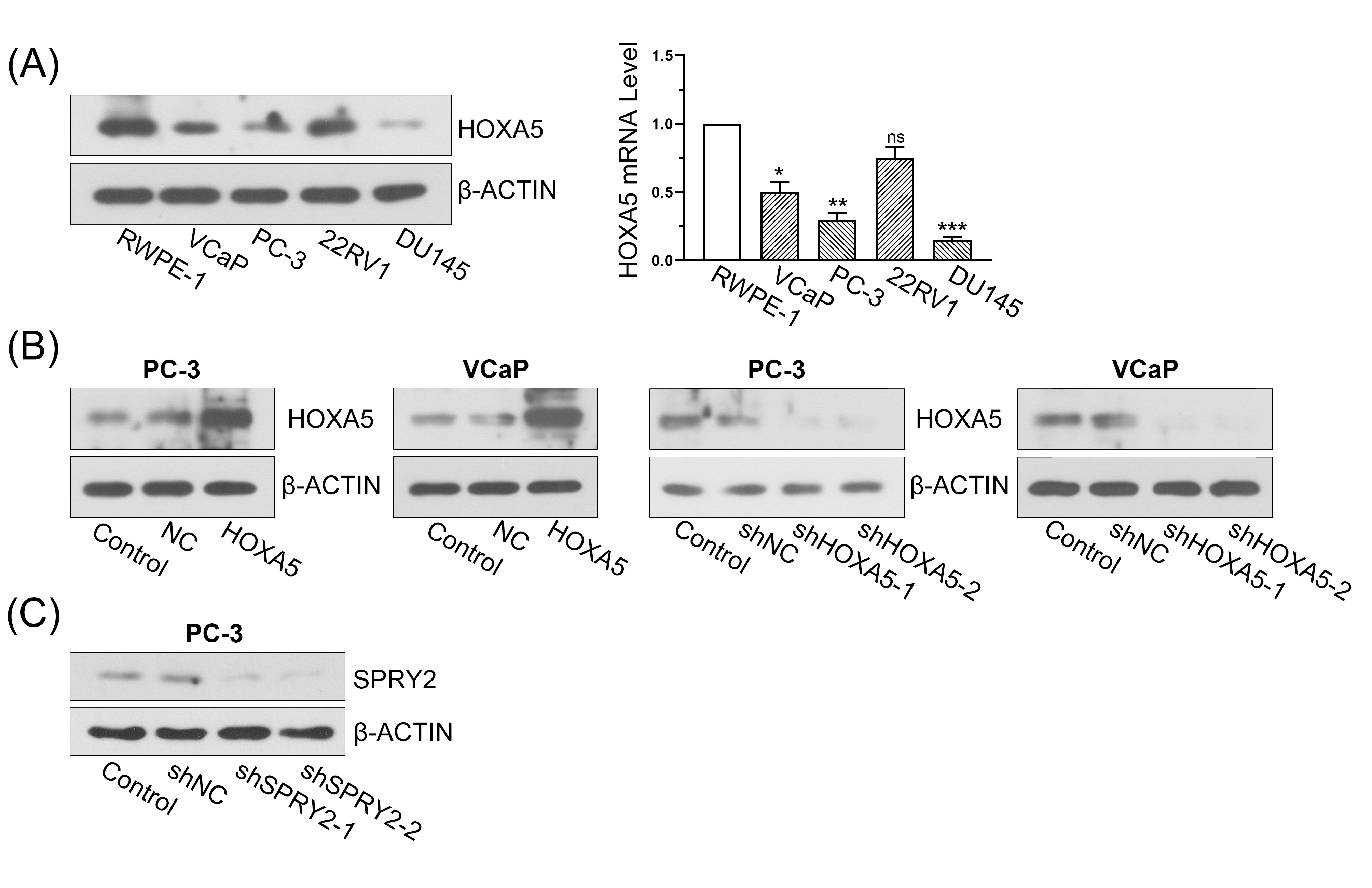

Supplement: Supplementary file 1 — Supplementary Figure 1 [file 41420_2023_1675_MOESM1_ESM.tif]
