# Supplementary material for: TRAF7-targeted HOXA5 acts as a tumor suppressor in prostate cancer progression and stemness via transcriptionally activating SPRY2 and regulating MEK/ERK signaling
Source: Cell Death Discov. 2023 Oct 16;9:378. doi: 10.1038/s41420-023-01675-9 (PMC10579307; doi:10.1038/s41420-023-01675-9)
Supplement: Supplementary file 2 — Supplementary figure legend [file 41420_2023_1675_MOESM2_ESM.docx]

**Supplementary Figure 1**

(A) RT-PCR and Western blot analysis of HOXA5 expression in four PCa cell lines (DU145, 22RV1, PC-3, and VCaP) as compared to non-malignant prostate cells (RWPE-1). (B) PCa cells were transfected with HOXA5 overexpression and shRNA plasmid for the establishment of stable cell lines. The effects were confirmed by Western blot analysis. The interfered efficiency of HOXA5 in PC-3, and VCaP cells was determined by Western blot analysis. (C) PCa-3 cells were transfected with SPRY2 shRNA plasmid. The effects were confirmed by Western blot analysis. ^*^P < 0.05, ^**^P < 0.01. Results were presented as mean ± SD.
