# Supplementary material for: TRAF7-targeted HOXA5 acts as a tumor suppressor in prostate cancer progression and stemness via transcriptionally activating SPRY2 and regulating MEK/ERK signaling
Source: Cell Death Discov. 2023 Oct 16;9:378. doi: 10.1038/s41420-023-01675-9 (PMC10579307; doi:10.1038/s41420-023-01675-9)
Supplement: Supplementary file 3 — Supplementary Table 1 [file 41420_2023_1675_MOESM3_ESM.docx]

**Supplementary Table 1**. The clinicopathological characteristics of PCa samples included in the study.

| *Characteristics* | *Median (IQR) or n (%)* |
| --- | --- |
| Patients (n) | 30 |
| Age (years) | 63.5 (58.0-73.8) |
| BMI (kg/m^2^) | 23.3 (21.5-27.9) |
| Prostate volume (mL) | 59.9 (52.5-69.1) |
| PSA (ng/mL) | 11.2 (7.9-19.5) |
| PSAD (ng/mL/cm^3^) | 0.19 (0.13-0.29) |
| Pathological Gleason score, n (%) |  |
| ≤6 | 5 (16.7) |
| 7 (3+4) | 5 (16.7) |
| 7 (4+3) | 13 (43.3) |
| ≥8 | 7 (23.3) |
| Pathological T stage, n (%) |  |
| pT2 | 16 (53.3) |
| pT3 | 11 (36.7) |
| pT4 | 3 (10.0) |
| Clinical risk stratification, n (%) |  |
| low | 5 (16.7) |
| intermediate | 16 (53.3) |
| high | 9 (30.0) |

PCa: prostate cancer; IQR: inter-quantile range; BMI: body mass index; PSA: prostate

specific antigen; PSAD: prostate specific antigen density; T: tumor
