# Supplementary material for: TRAF7-targeted HOXA5 acts as a tumor suppressor in prostate cancer progression and stemness via transcriptionally activating SPRY2 and regulating MEK/ERK signaling
Source: Cell Death Discov. 2023 Oct 16;9:378. doi: 10.1038/s41420-023-01675-9 (PMC10579307; doi:10.1038/s41420-023-01675-9)
Supplement: Supplementary file 4 — Supplementary Table 2 [file 41420_2023_1675_MOESM4_ESM.docx]

**Supplementary Table 2. Antibodies used in Western blot**

| Primary antibodies | Dilution | Company | Catalog No. |
| --- | --- | --- | --- |
| HOXA5 | 1:300 | Santa Cruz Biotechnology | sc-365784 |
| Nanog | 1:1000 | Boster Biological Technology | A00153-3 |
| CD44 | 1:500 | ABclonal | A12410 |
| Oct4 | 1:500 | ABclonal | A7920 |
| SOX2 | 1:500 | Boster Biological Technology | BA3292 |
| MMP-2 | 1：1000 | Proteintech | 10373-2-AP |
| MMP-9 | 1：500 | Affinity | AF5228 |
| N-cadherin | 1:400 | ABclonal | A19083 |
| E-cadherin | 1:1000 | ABclonal | A20798 |
| Vimentin | 1:500 | ABclonal | A19607 |
| p-MEK1/2 | 1:500 | ABclonal | AP0209 |
| MEK1/2 | 1:500 | Affinity | AF6385 |
| p-ERK1/2 | 1:1000 | Affinity | AF8208 |
| ERK1/2 | 1:1000 | Affinity | AF6240 |
| TRAF7 | 1:500 | Proteintech | 11780-1-AP |
| SPRY2 | 1:500 | ABclonal | A8611 |
| β-actin | 1:2000 | Proteintech | 60008-1-Ig |
| Secondary antibody | Dilution | Company | Catalog No. |
| HRP Goat Anti-Rabbit | 1:10000 | Proteintech | SA00001-2 |
| HRP Goat Anti-Mouse | 1:10000 | Proteintech | SA00001-1 |
