# Supplementary material for: TRAF7-targeted HOXA5 acts as a tumor suppressor in prostate cancer progression and stemness via transcriptionally activating SPRY2 and regulating MEK/ERK signaling
Source: Cell Death Discov. 2023 Oct 16;9:378. doi: 10.1038/s41420-023-01675-9 (PMC10579307; doi:10.1038/s41420-023-01675-9)
Supplement: Supplementary file 6 — Supplementary material for original WB [file 41420_2023_1675_MOESM6_ESM.pptx]

## Slide 1
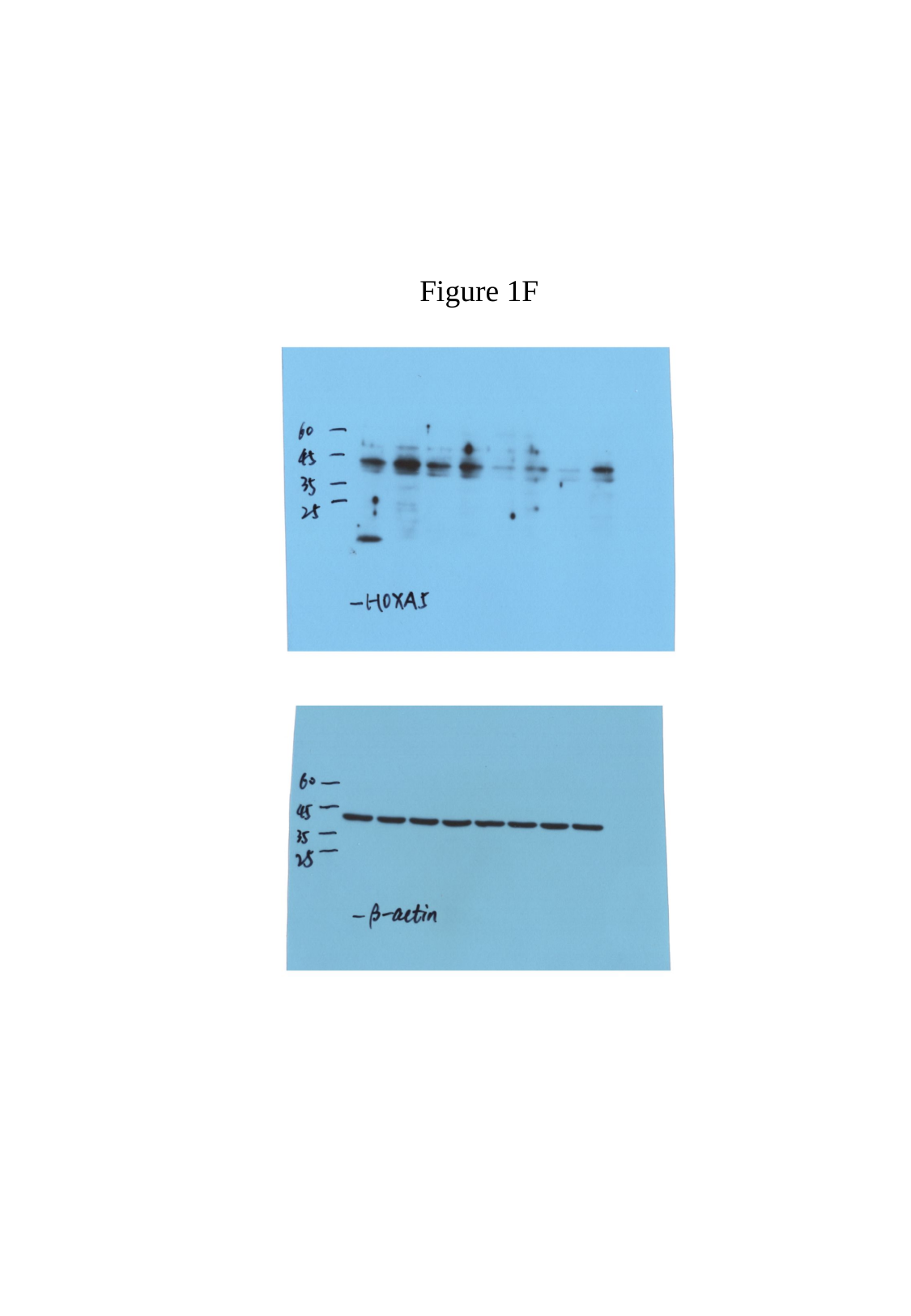

Figure 1F

## Slide 2
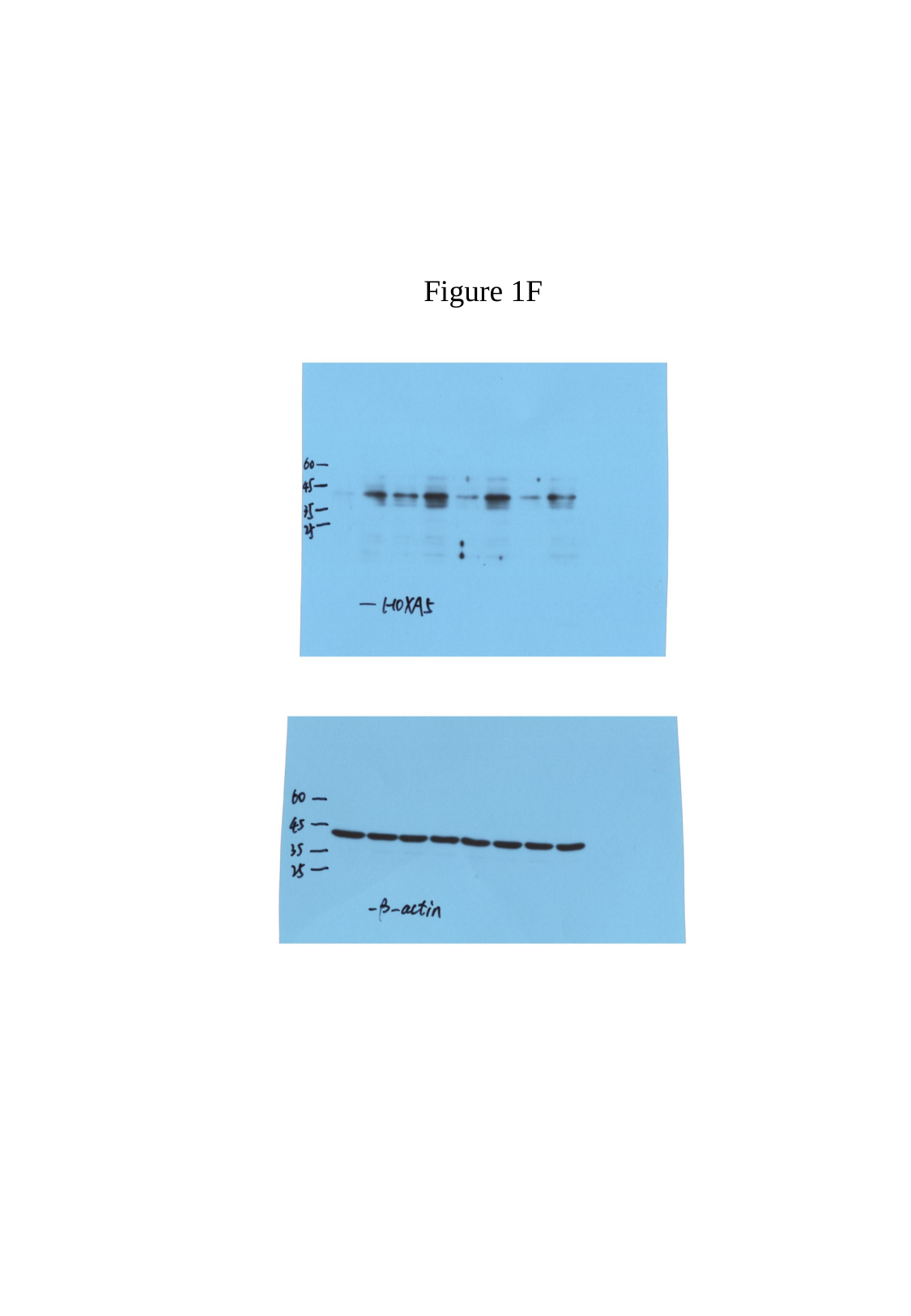

Figure 1F

## Slide 3
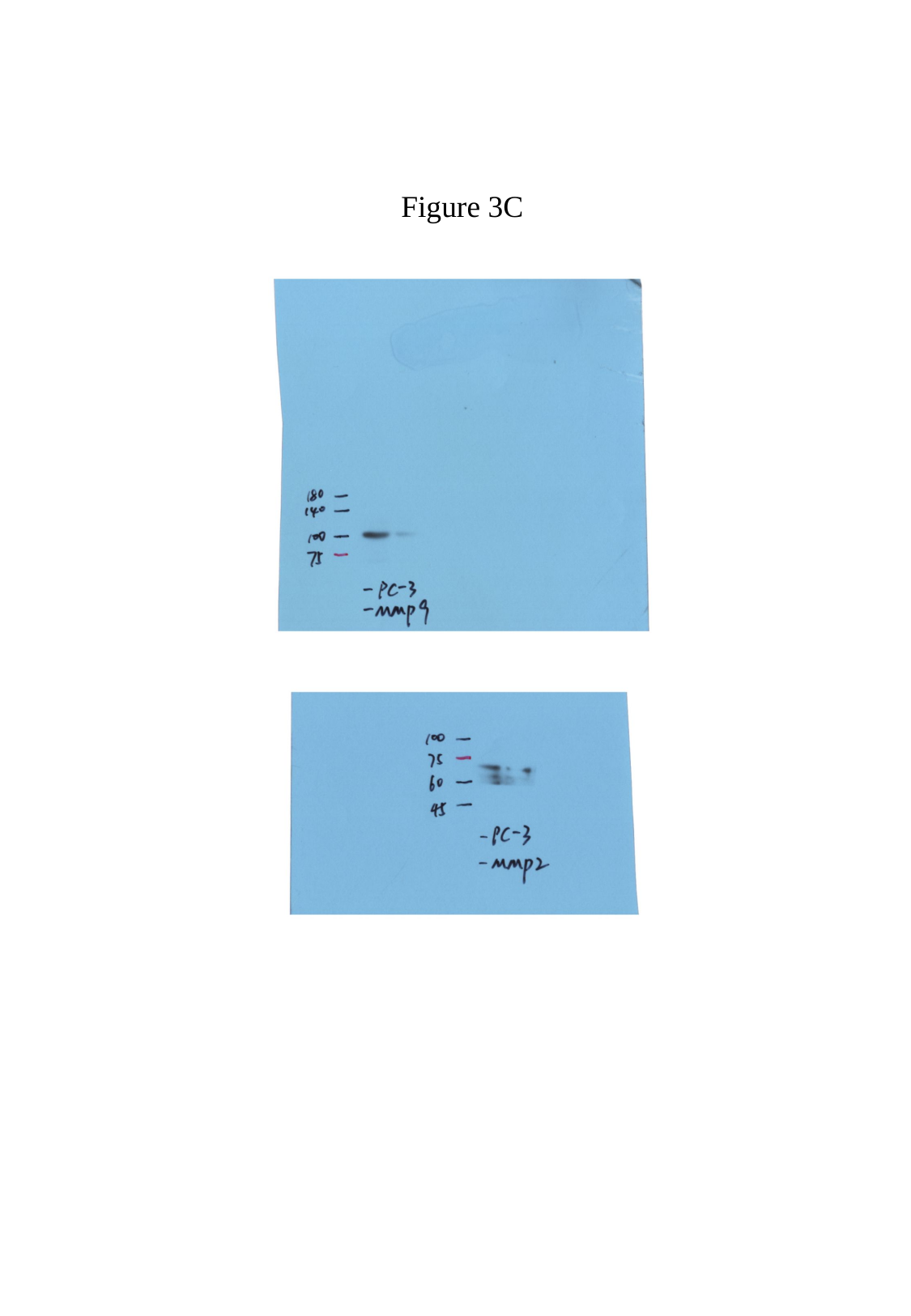

Figure 3C

## Slide 4
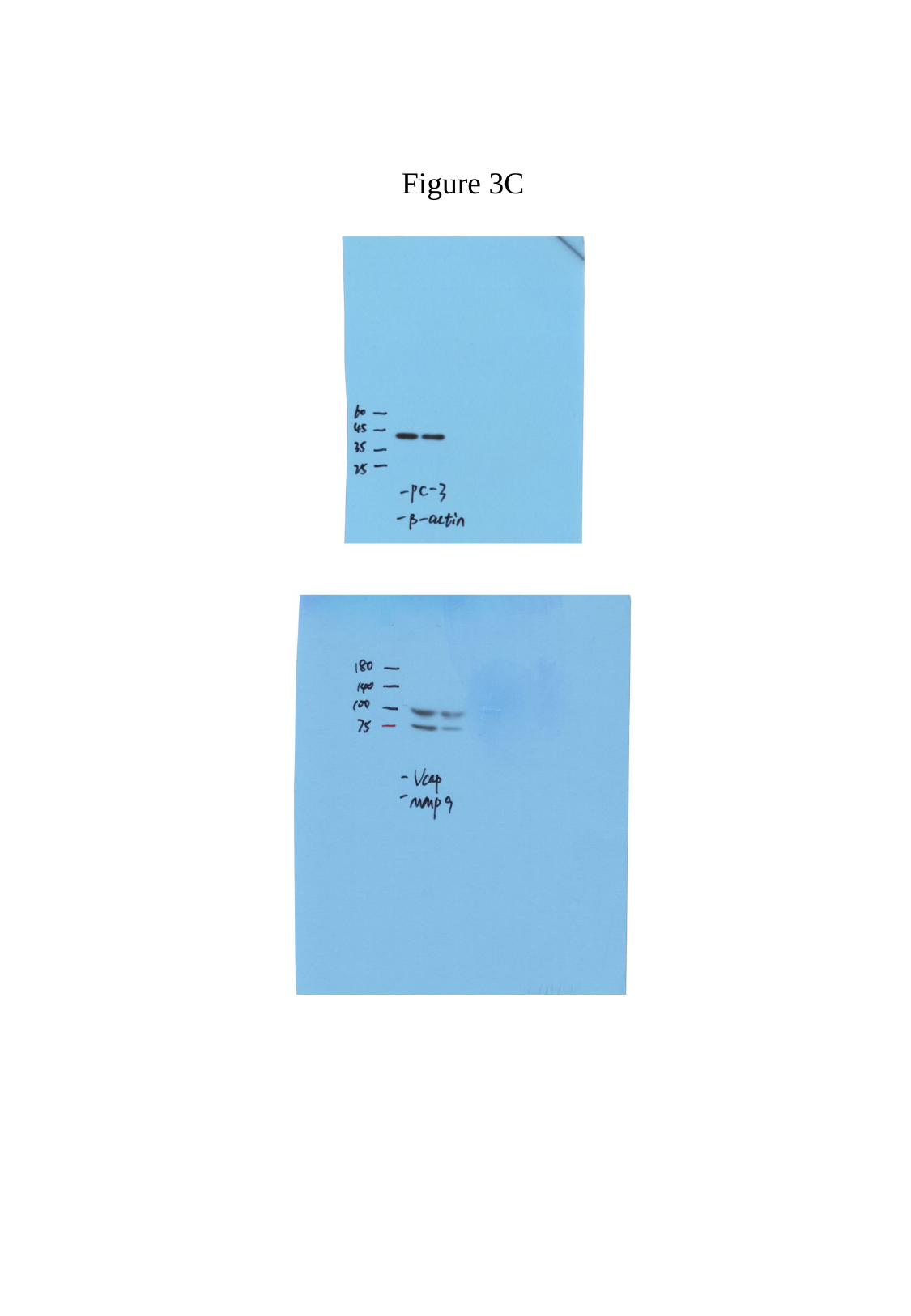

Figure 3C

## Slide 5
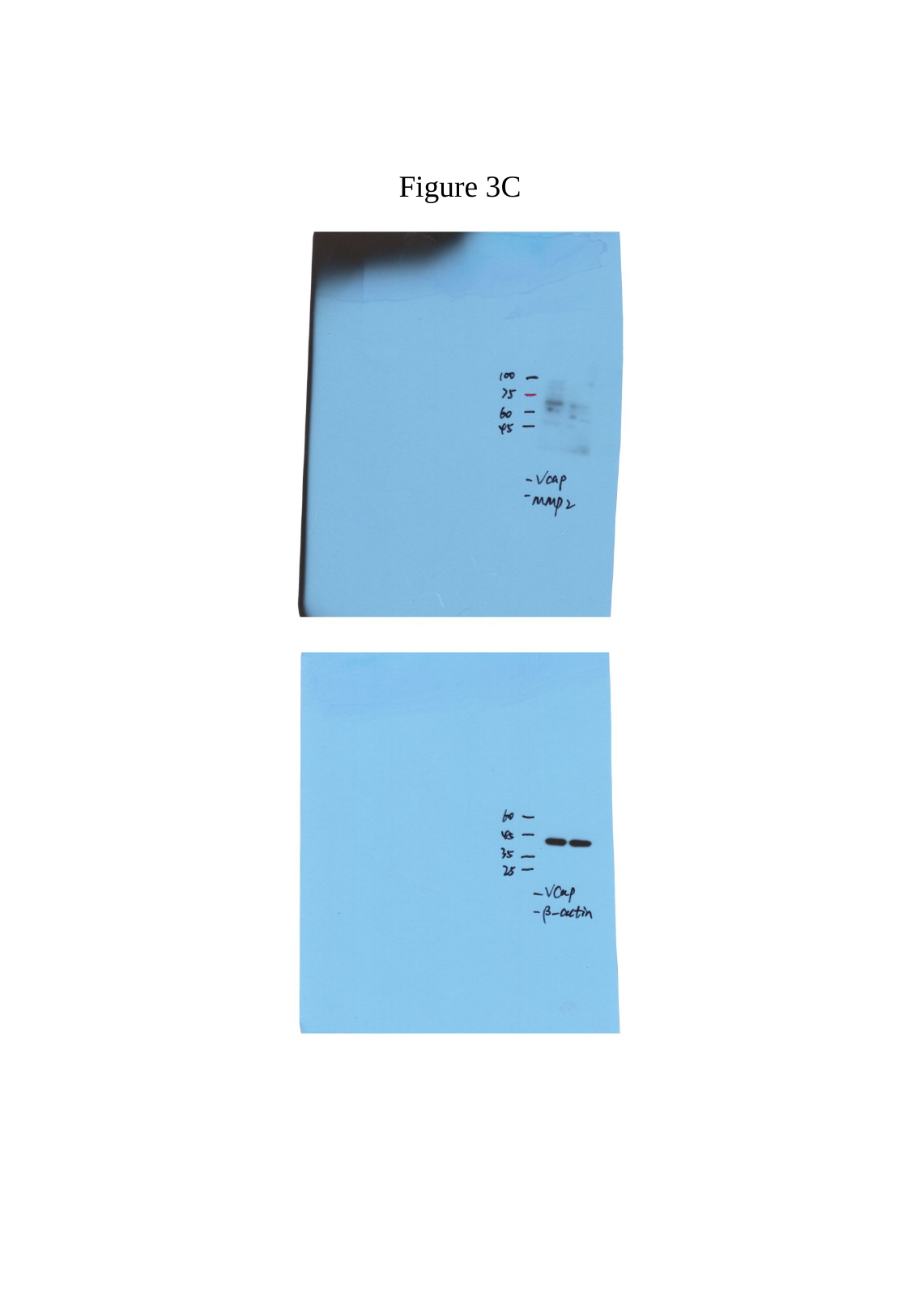

Figure 3C

## Slide 6
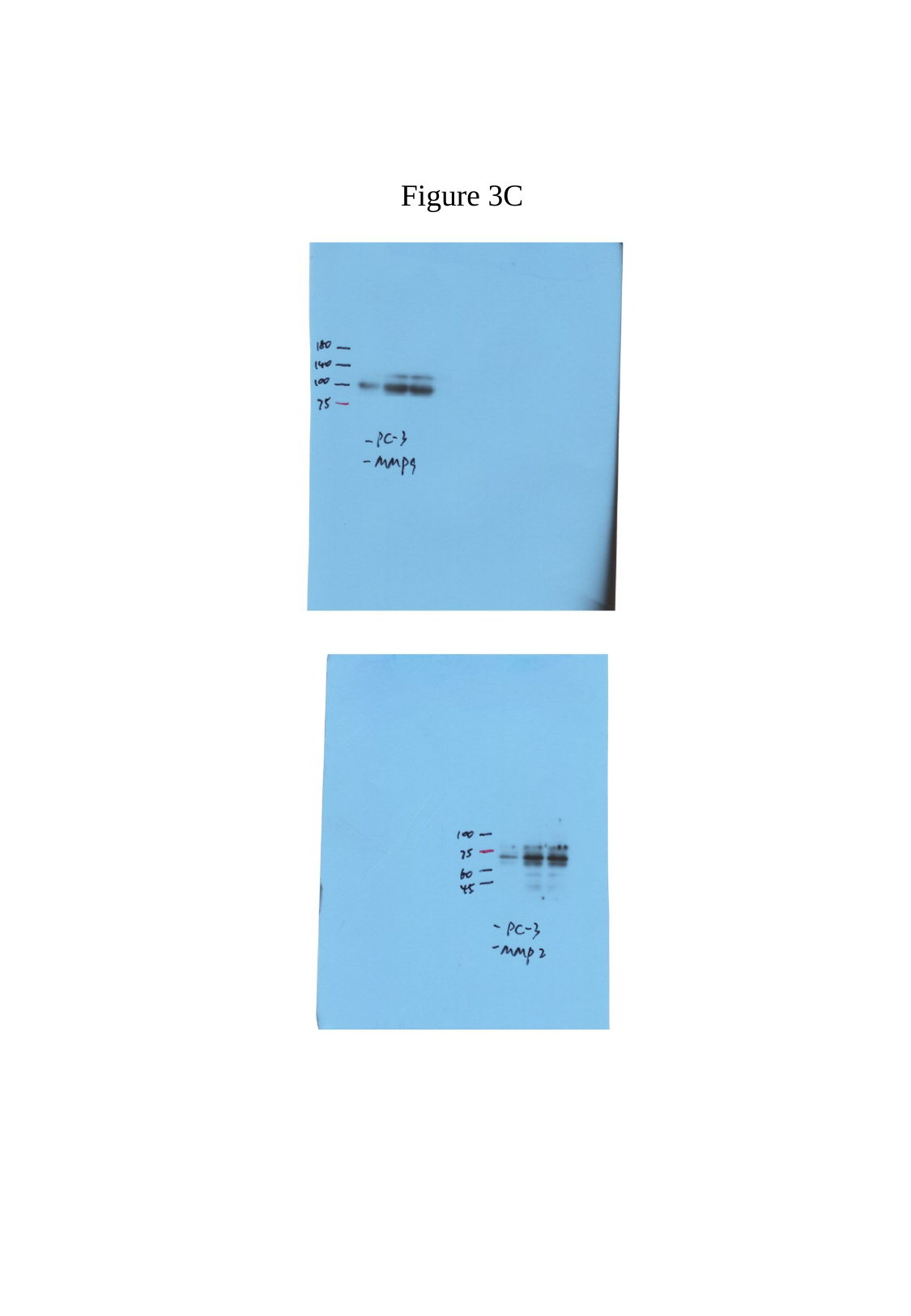

Figure 3C

## Slide 7
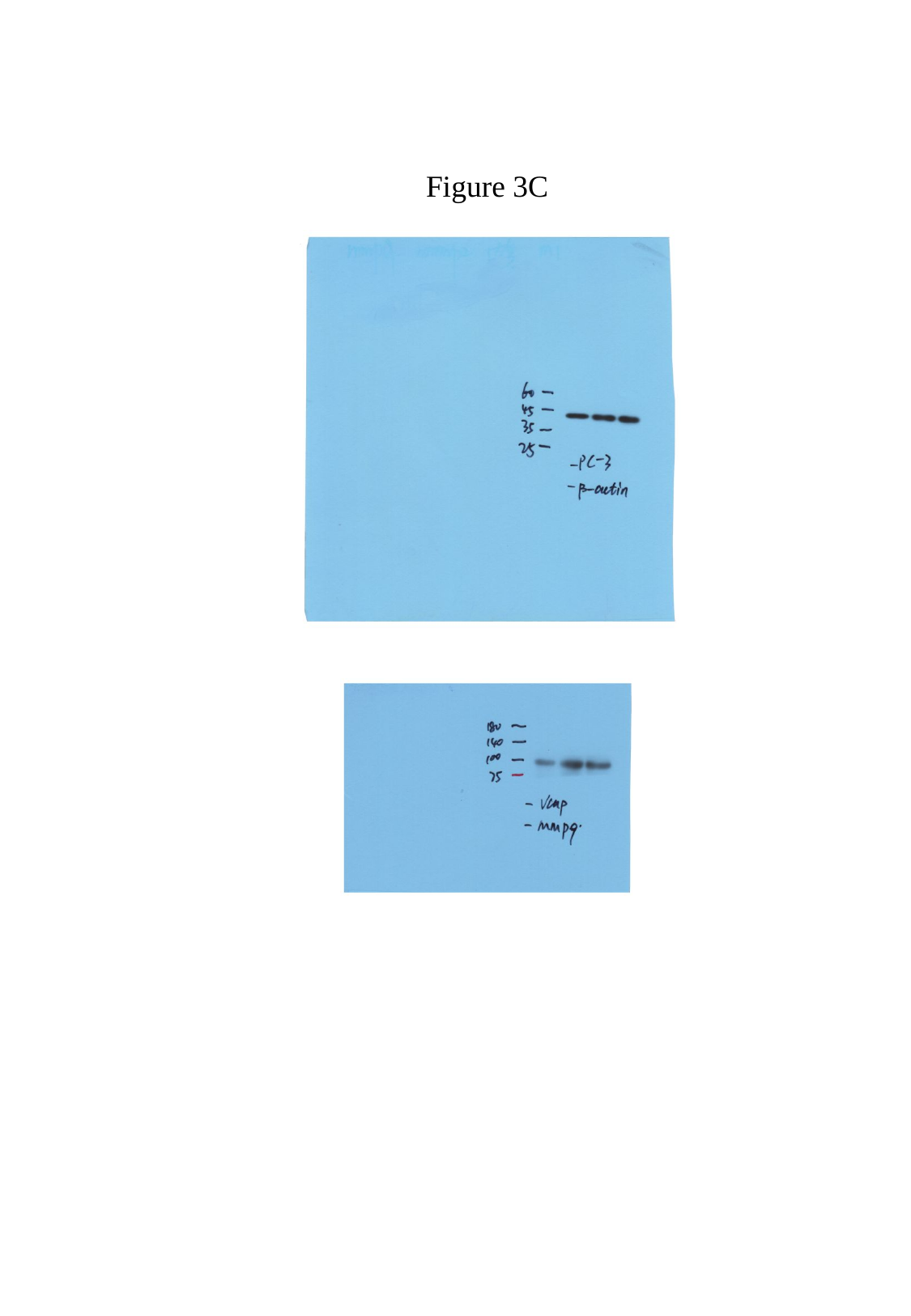

Figure 3C

## Slide 8
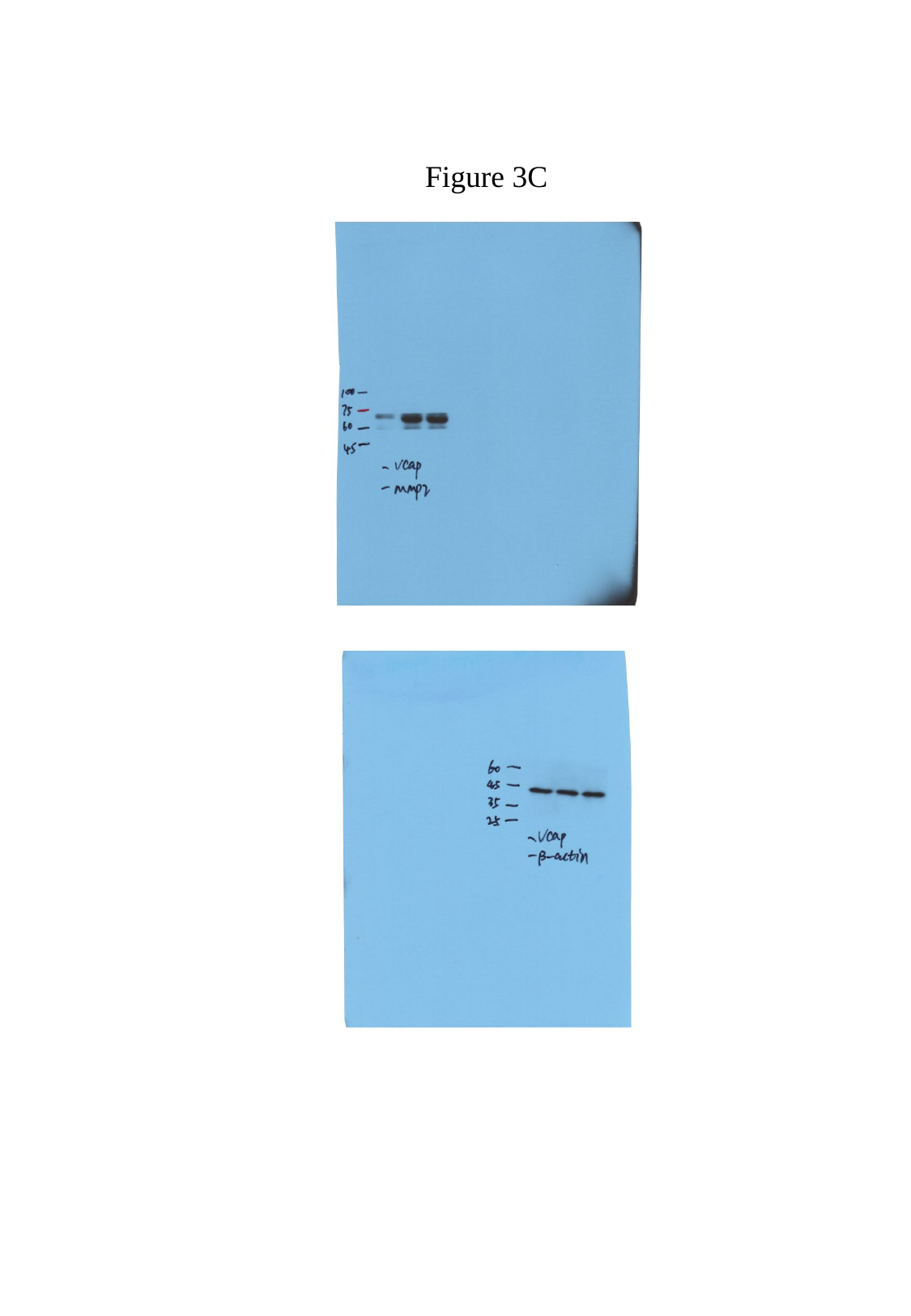

Figure 3C

## Slide 9
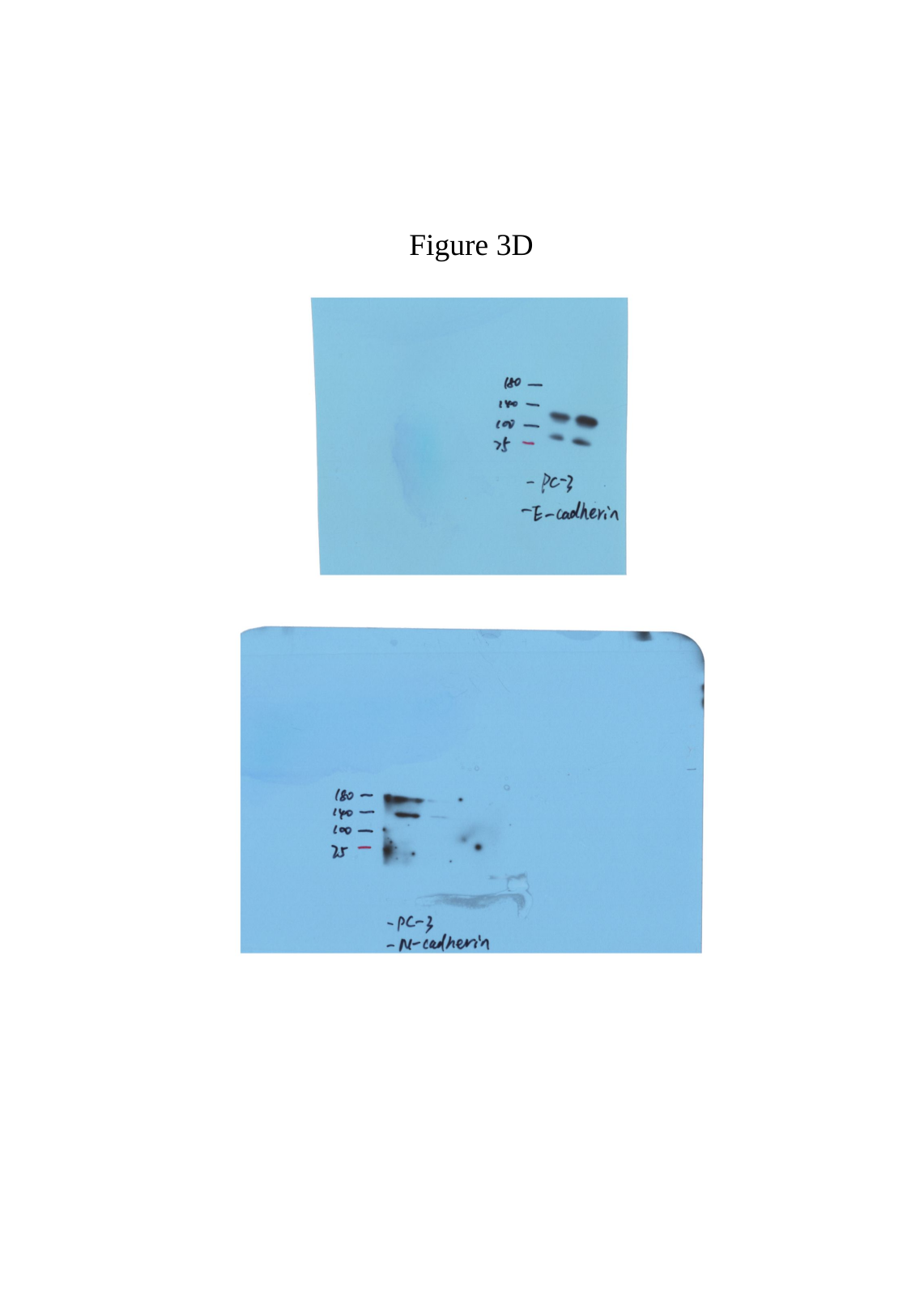

Figure 3D

## Slide 10
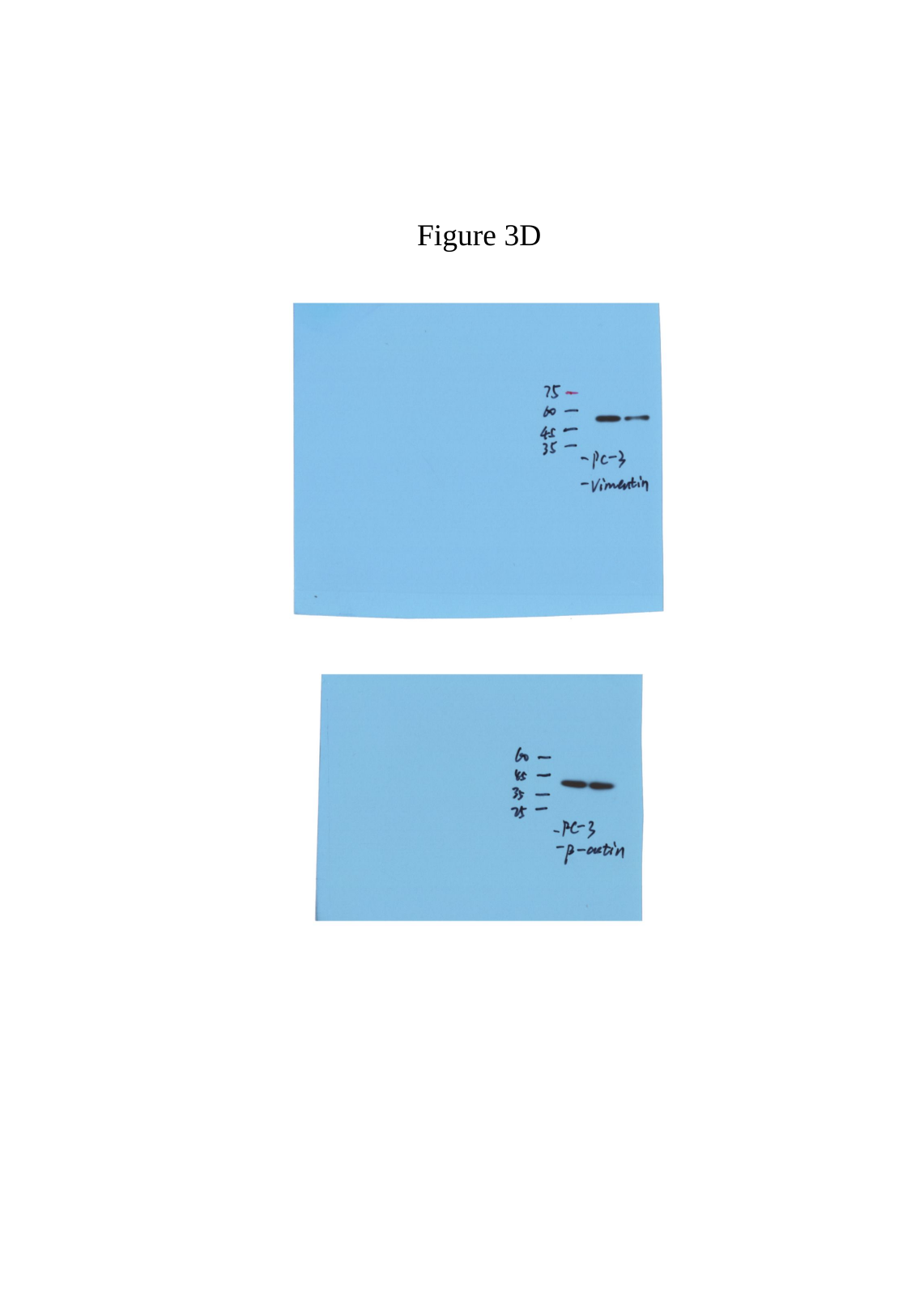

Figure 3D

## Slide 11
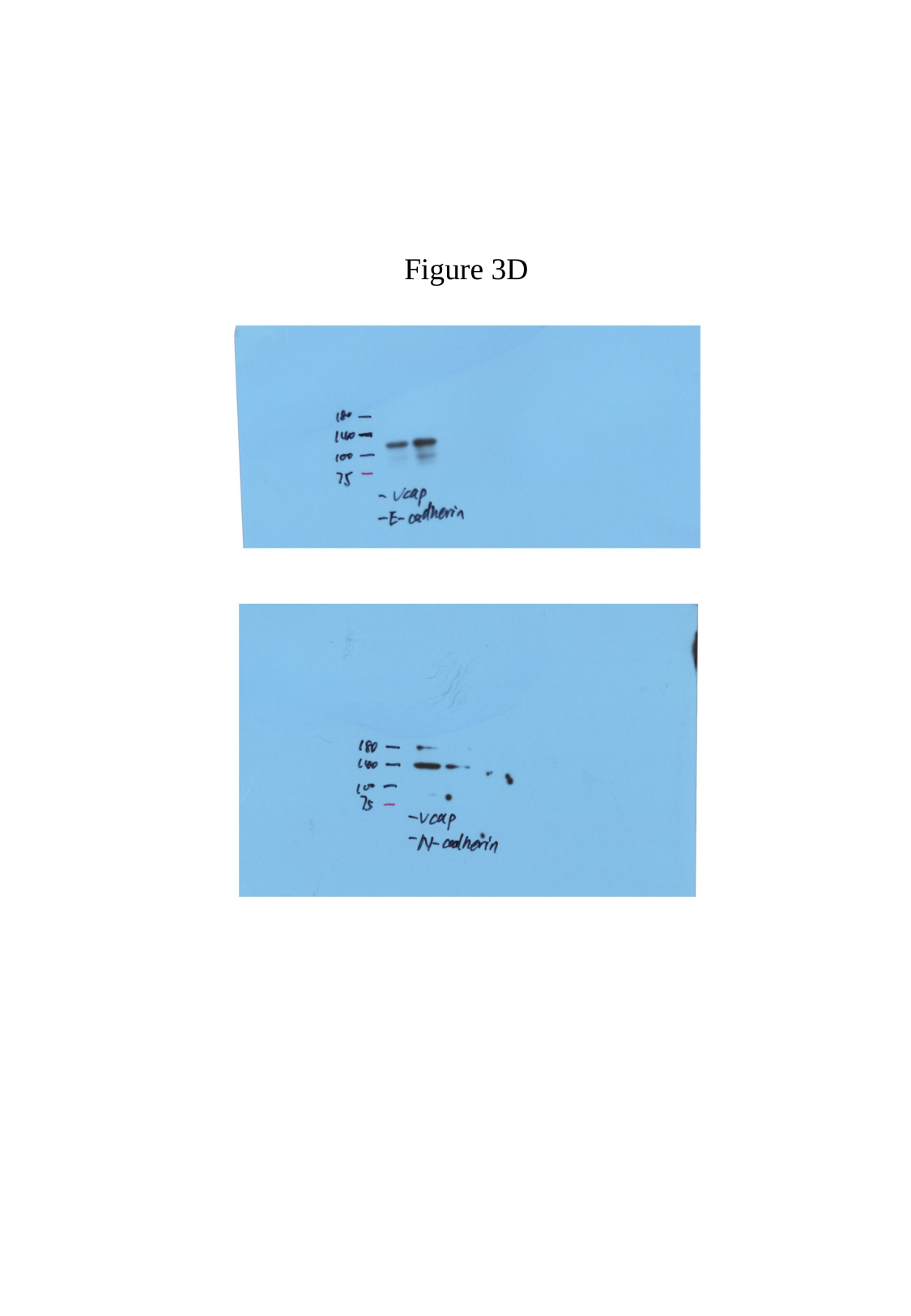

Figure 3D

## Slide 12
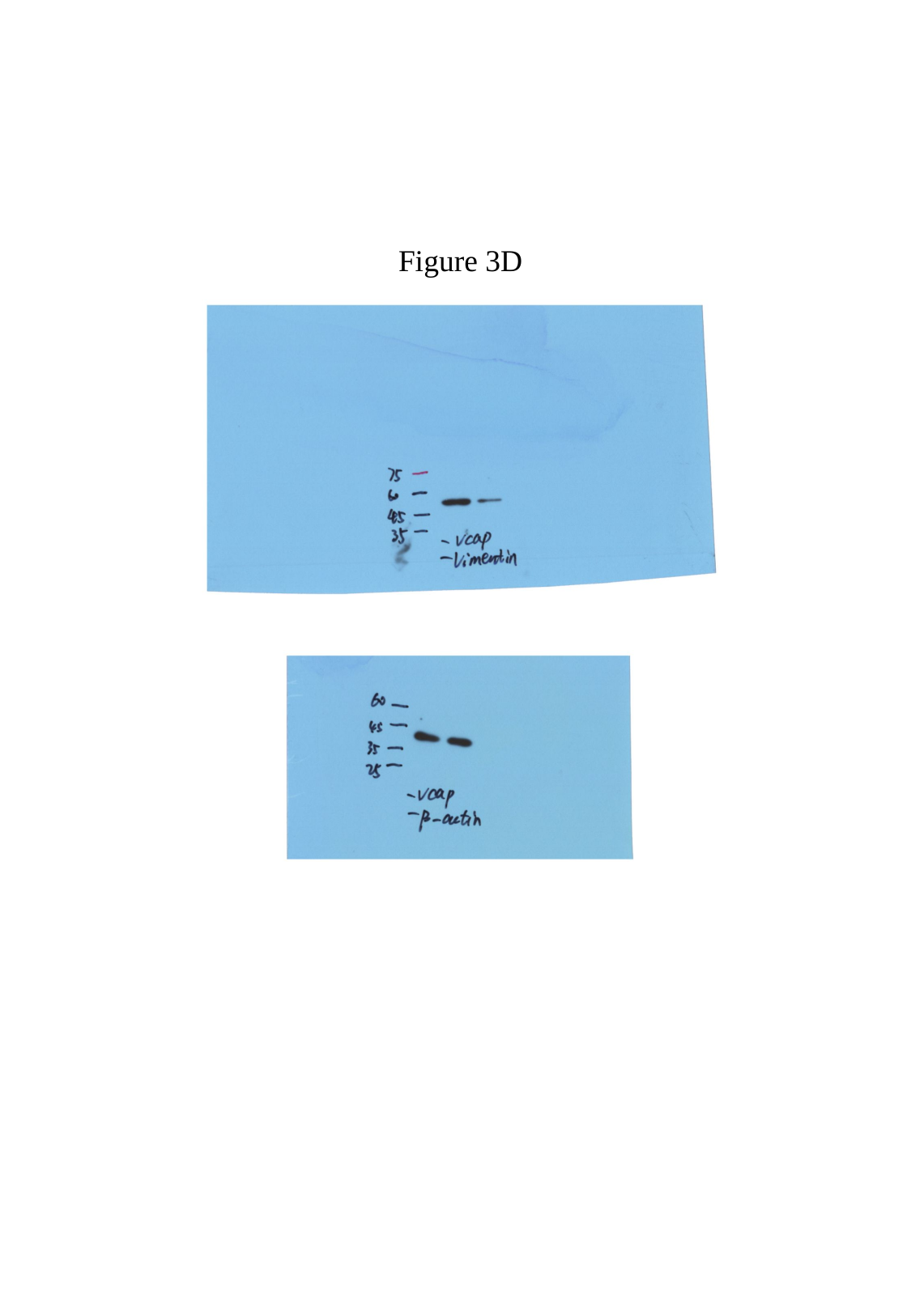

Figure 3D

## Slide 13
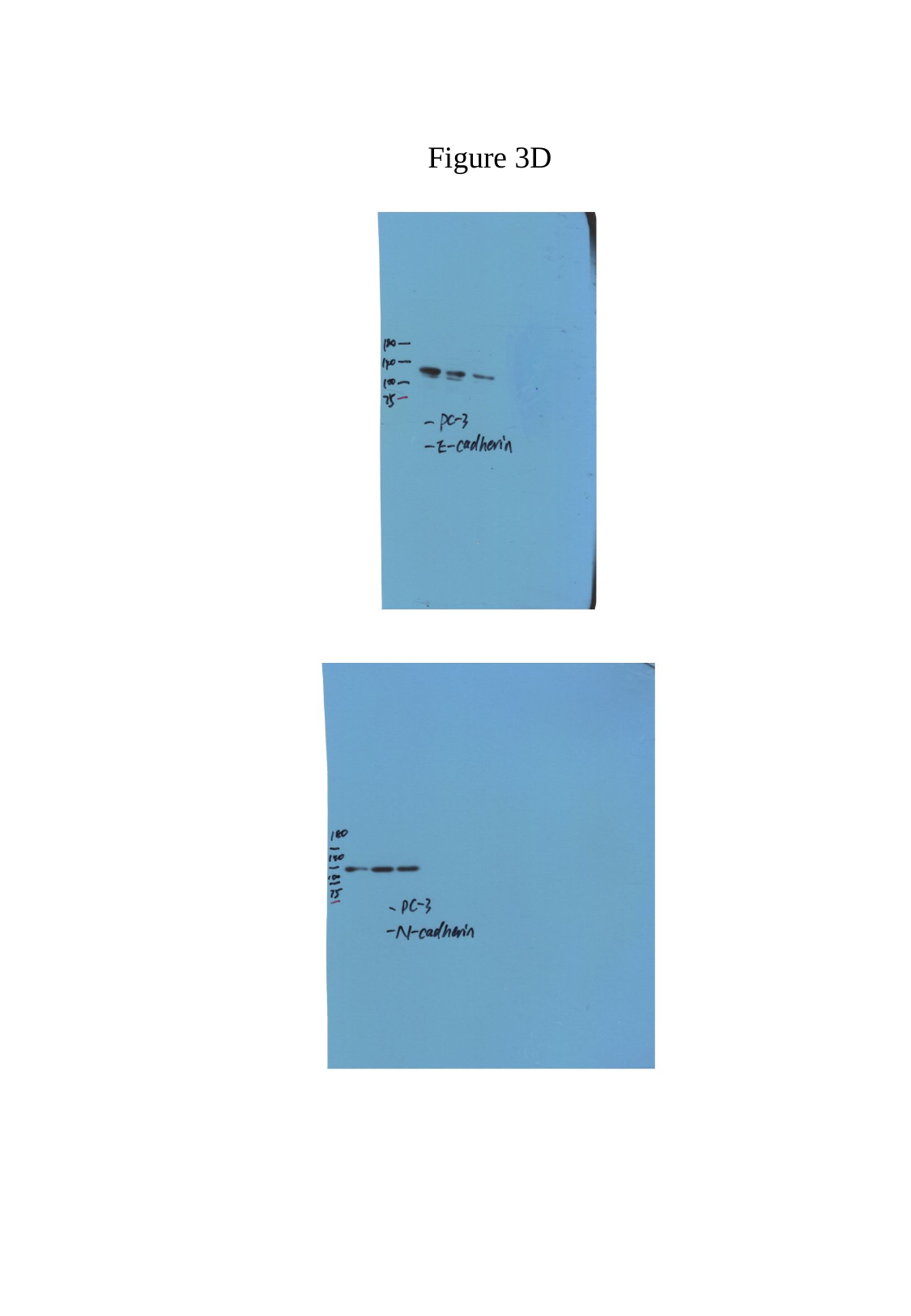

Figure 3D

## Slide 14
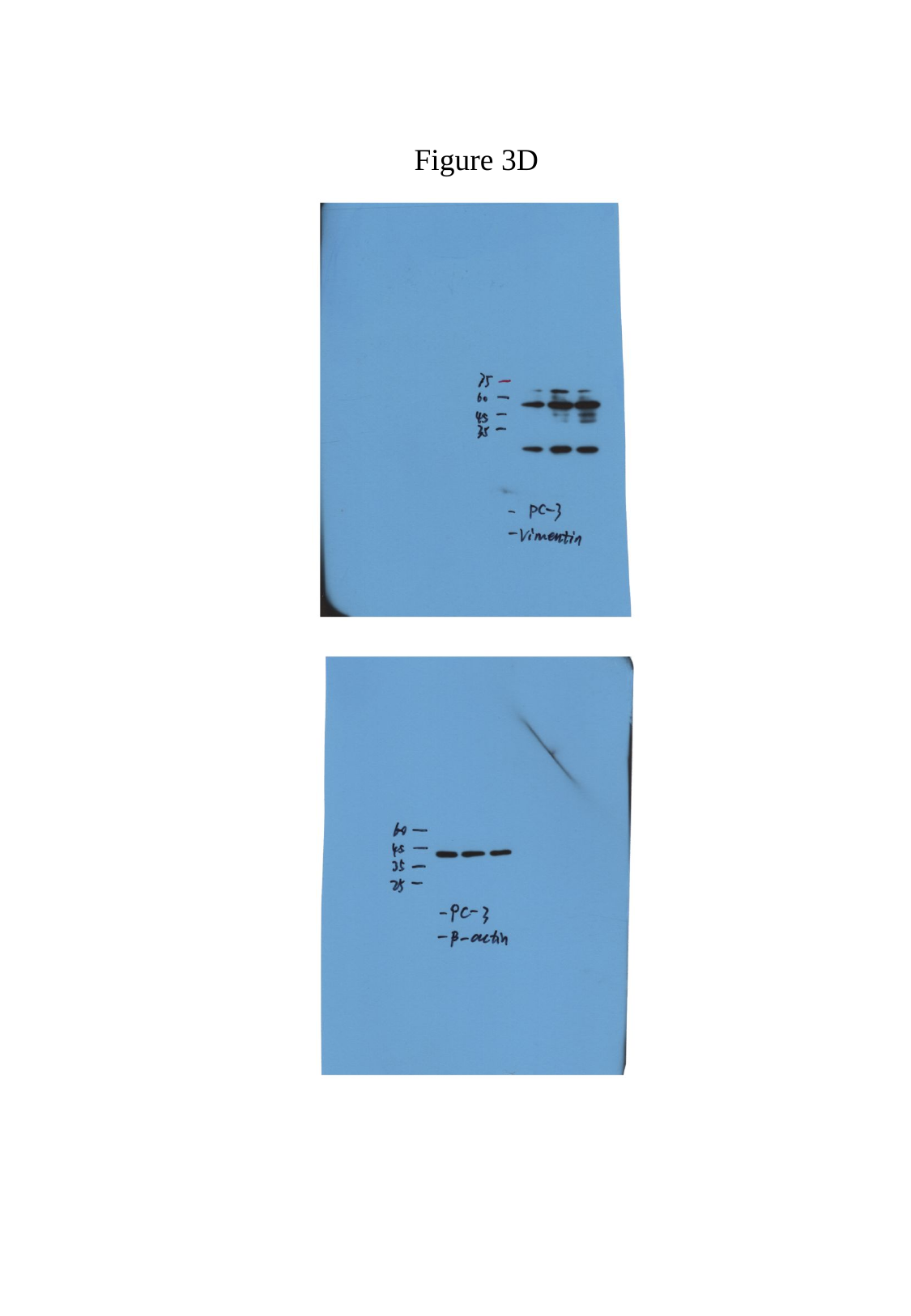

Figure 3D

## Slide 15
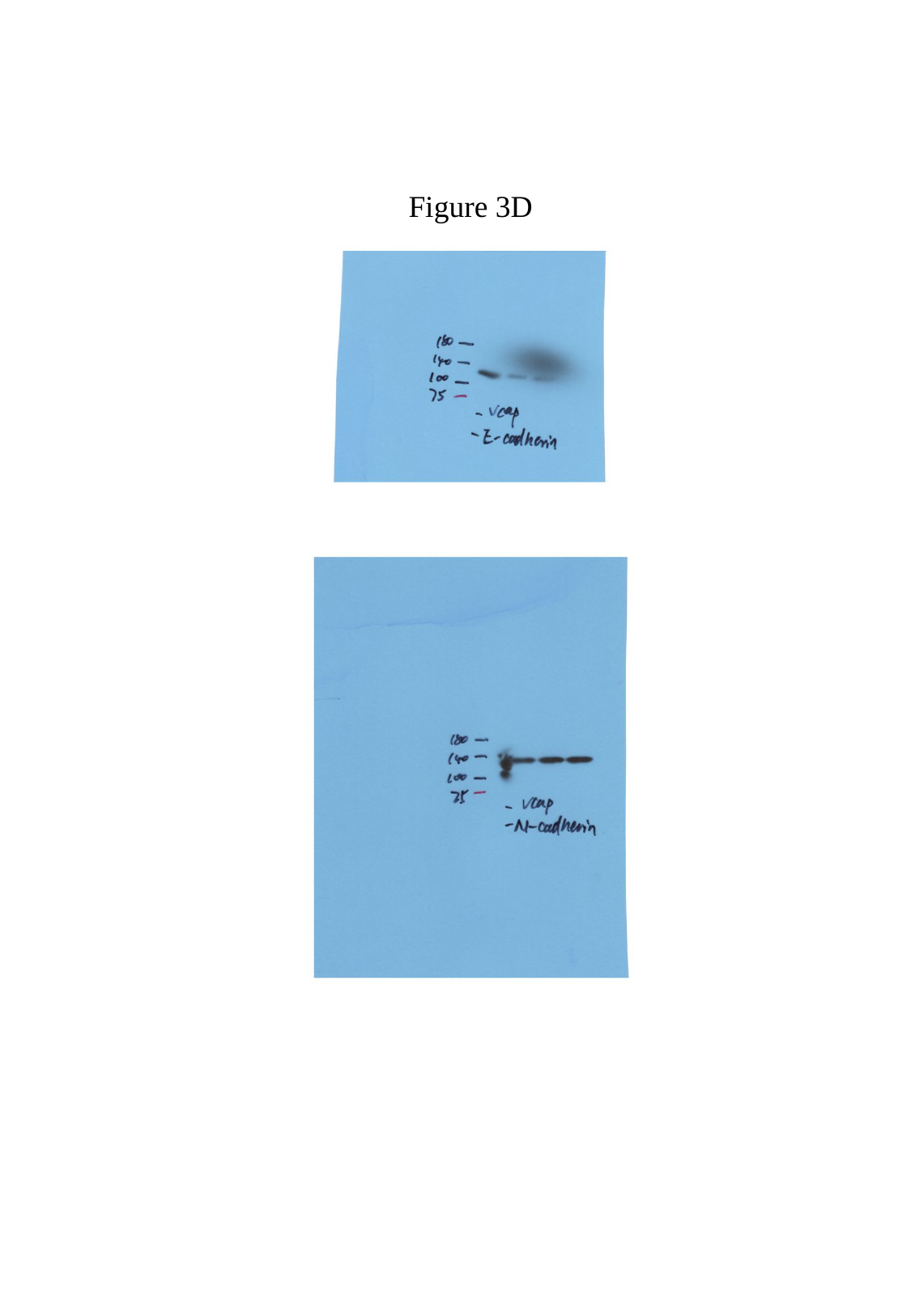

Figure 3D

## Slide 16
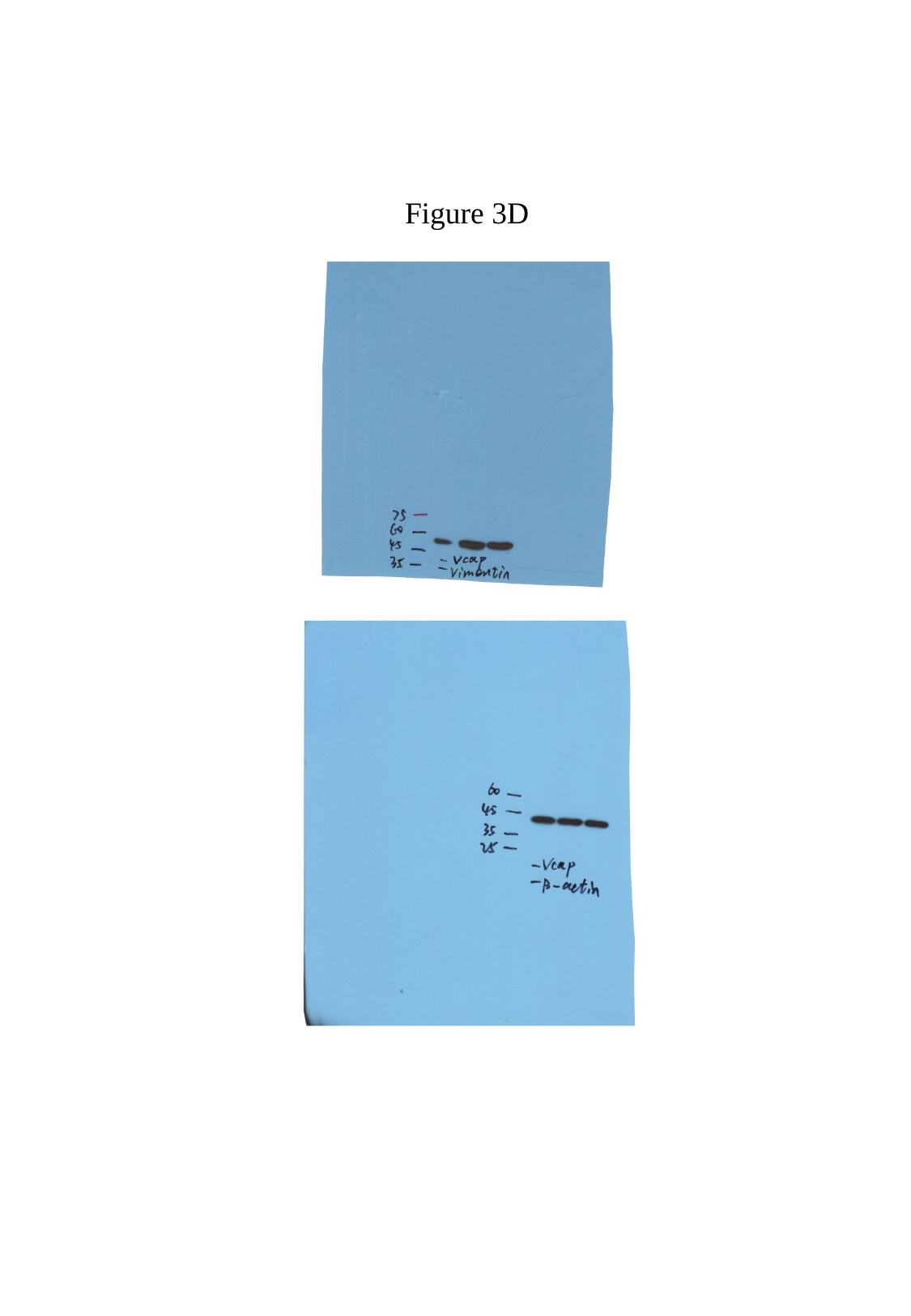

Figure 3D

## Slide 17
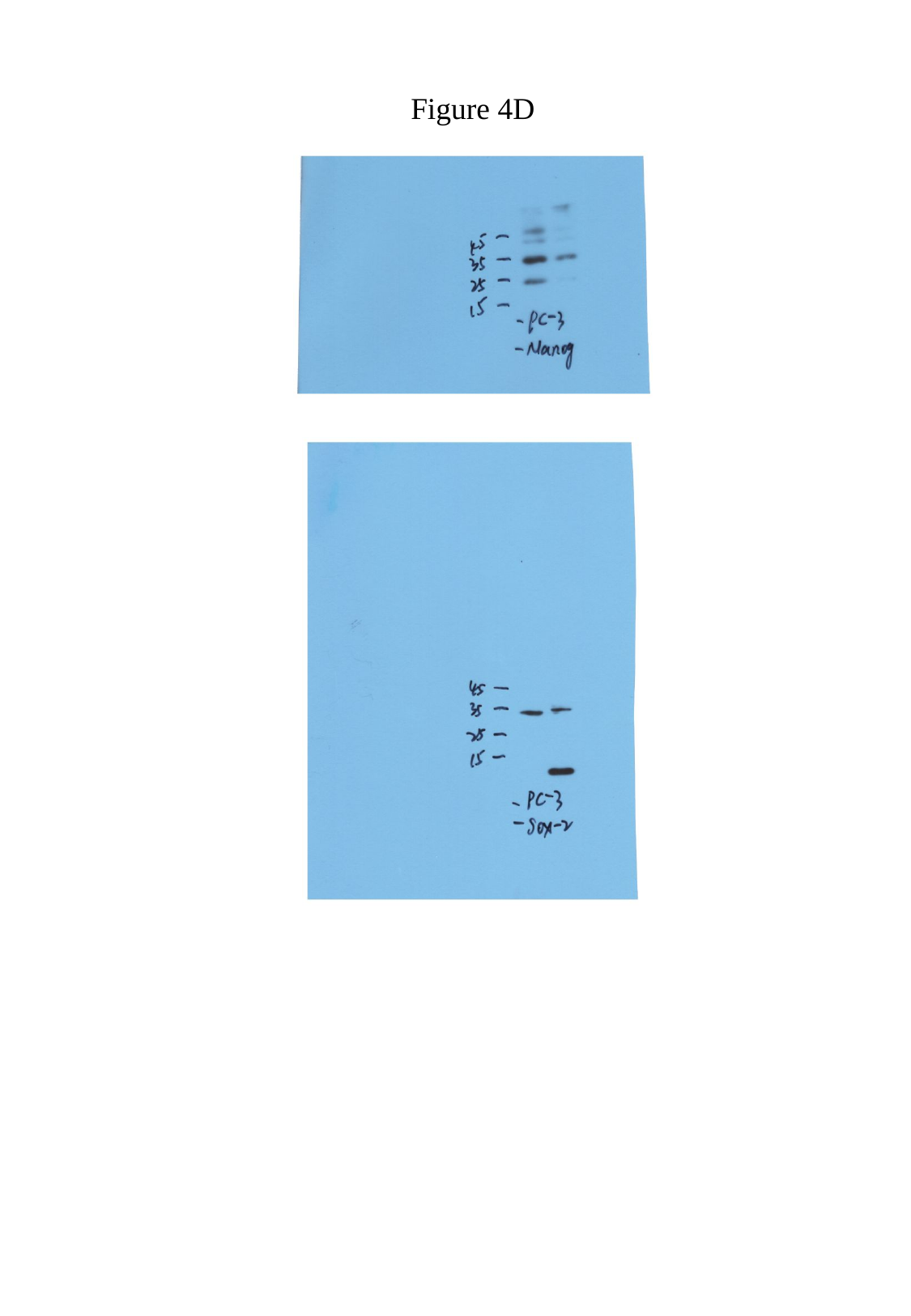

Figure 4D

## Slide 18
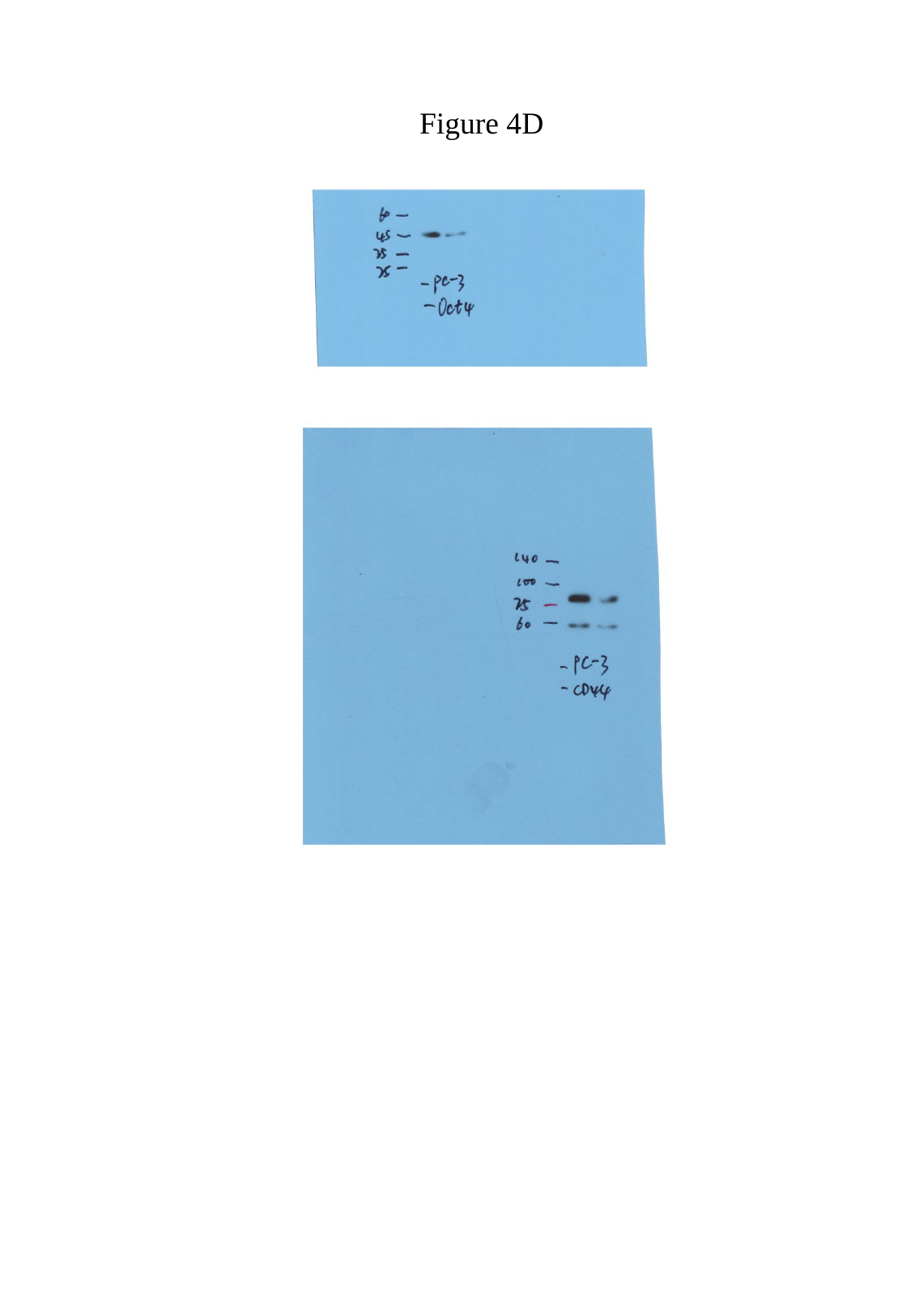

Figure 4D

## Slide 19
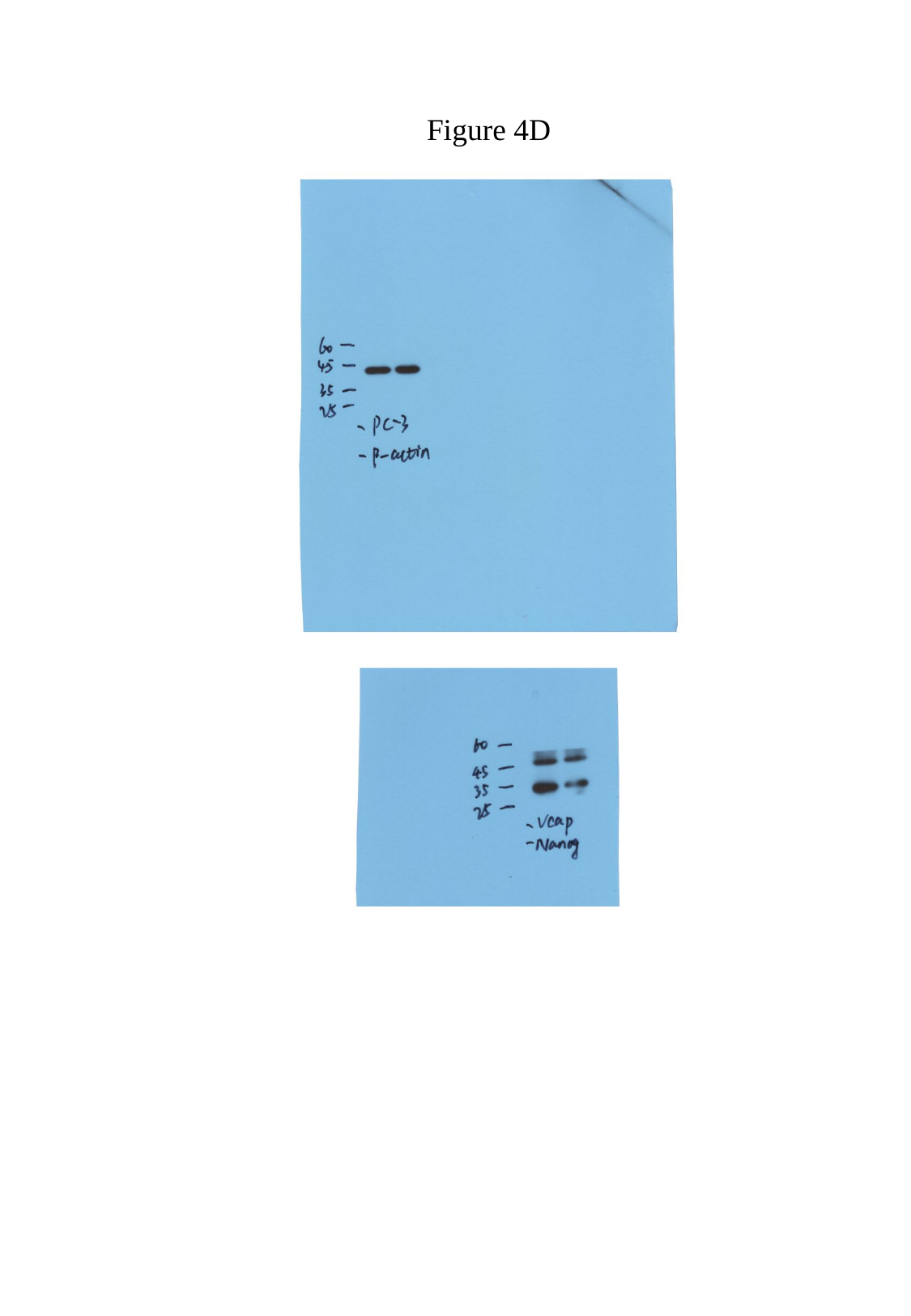

Figure 4D

## Slide 20
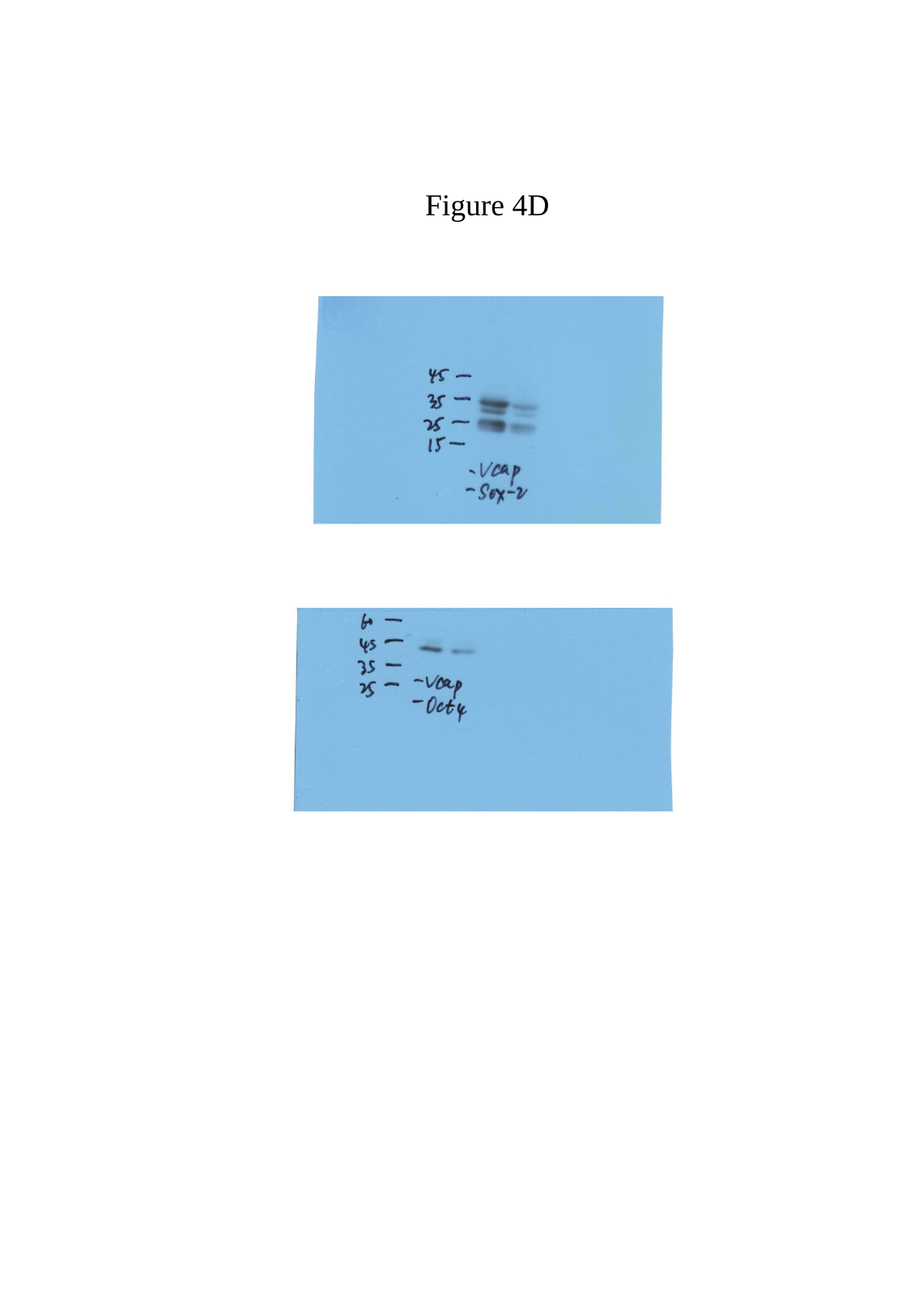

Figure 4D

## Slide 21
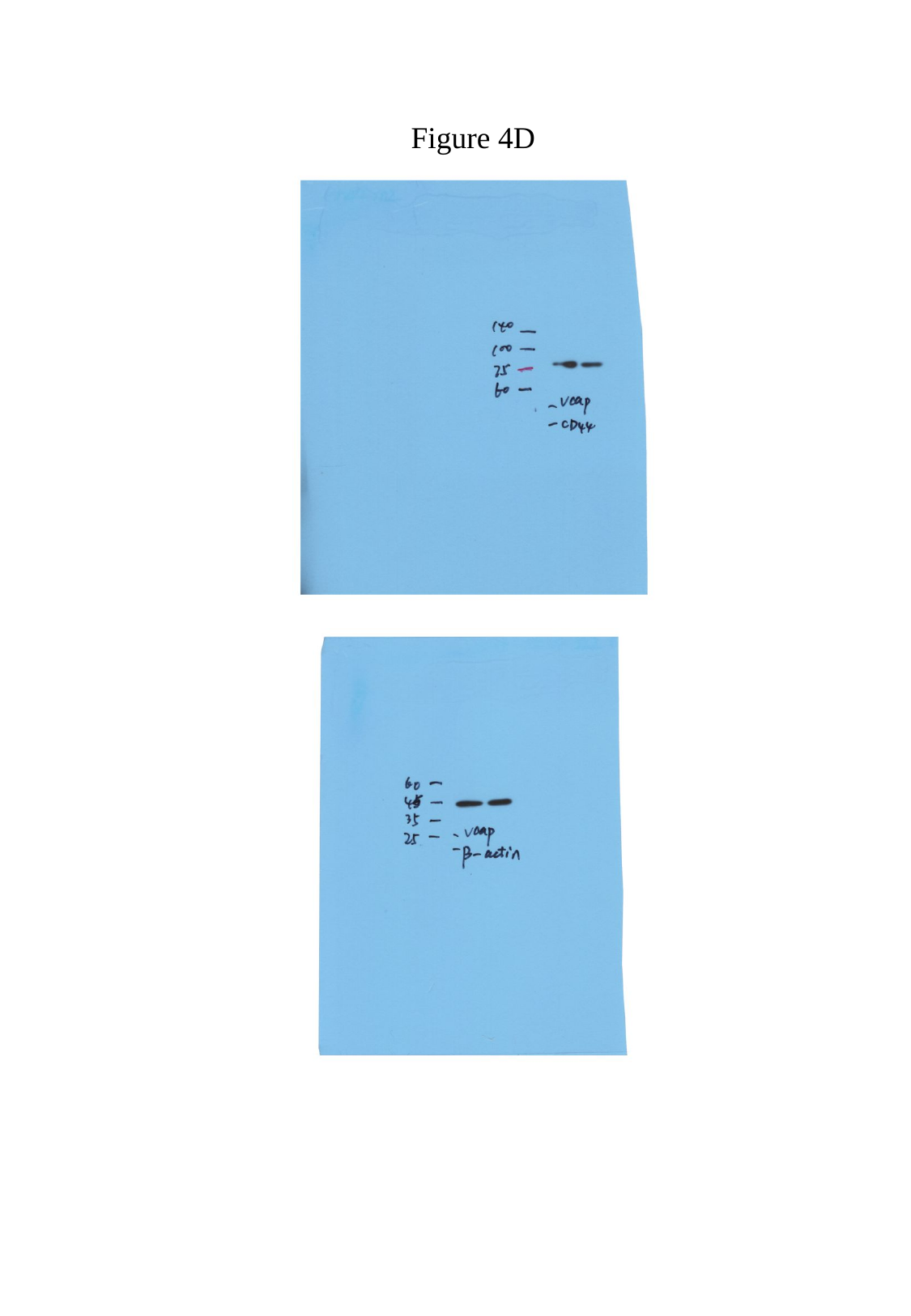

Figure 4D

## Slide 22
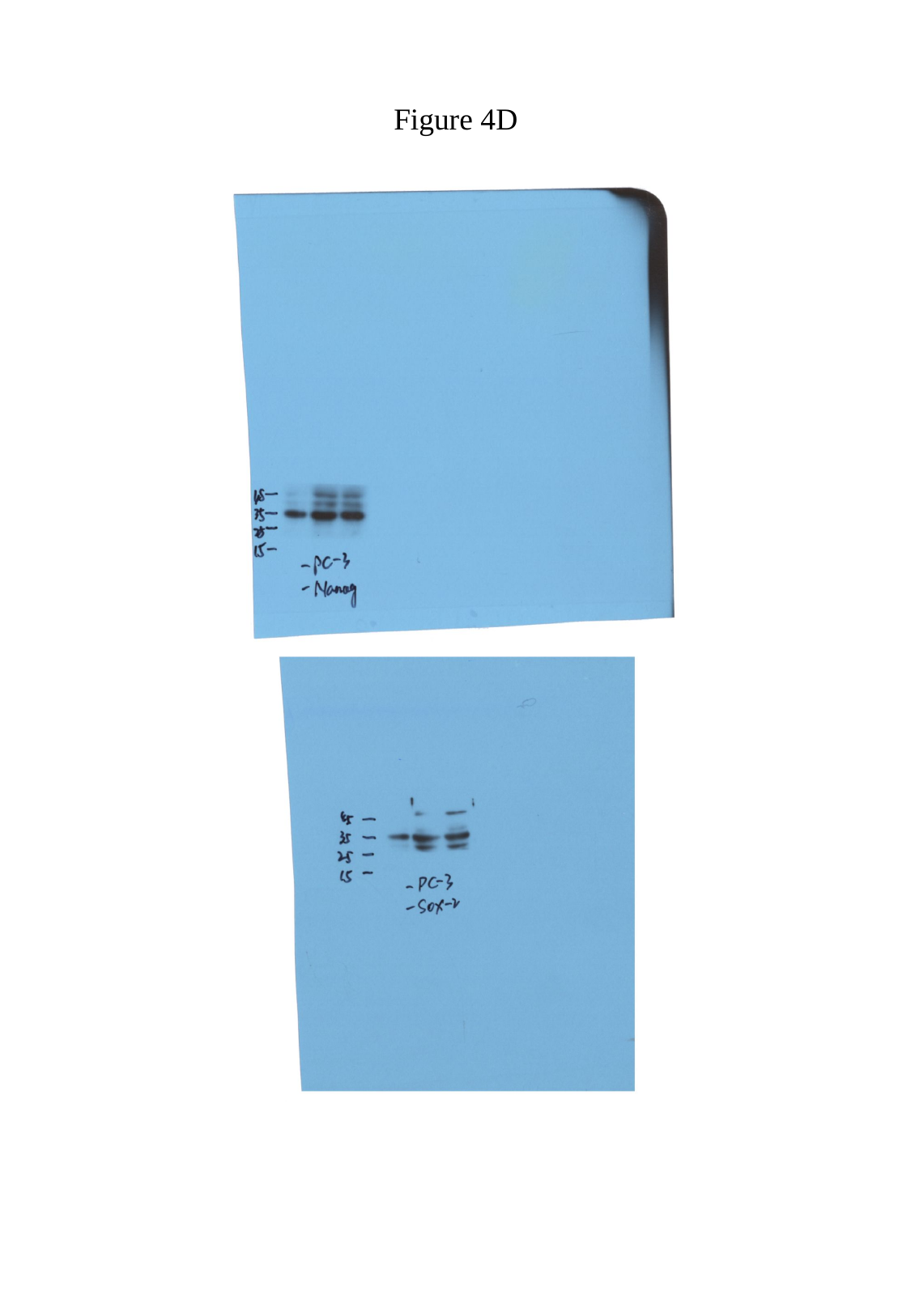

Figure 4D

## Slide 23
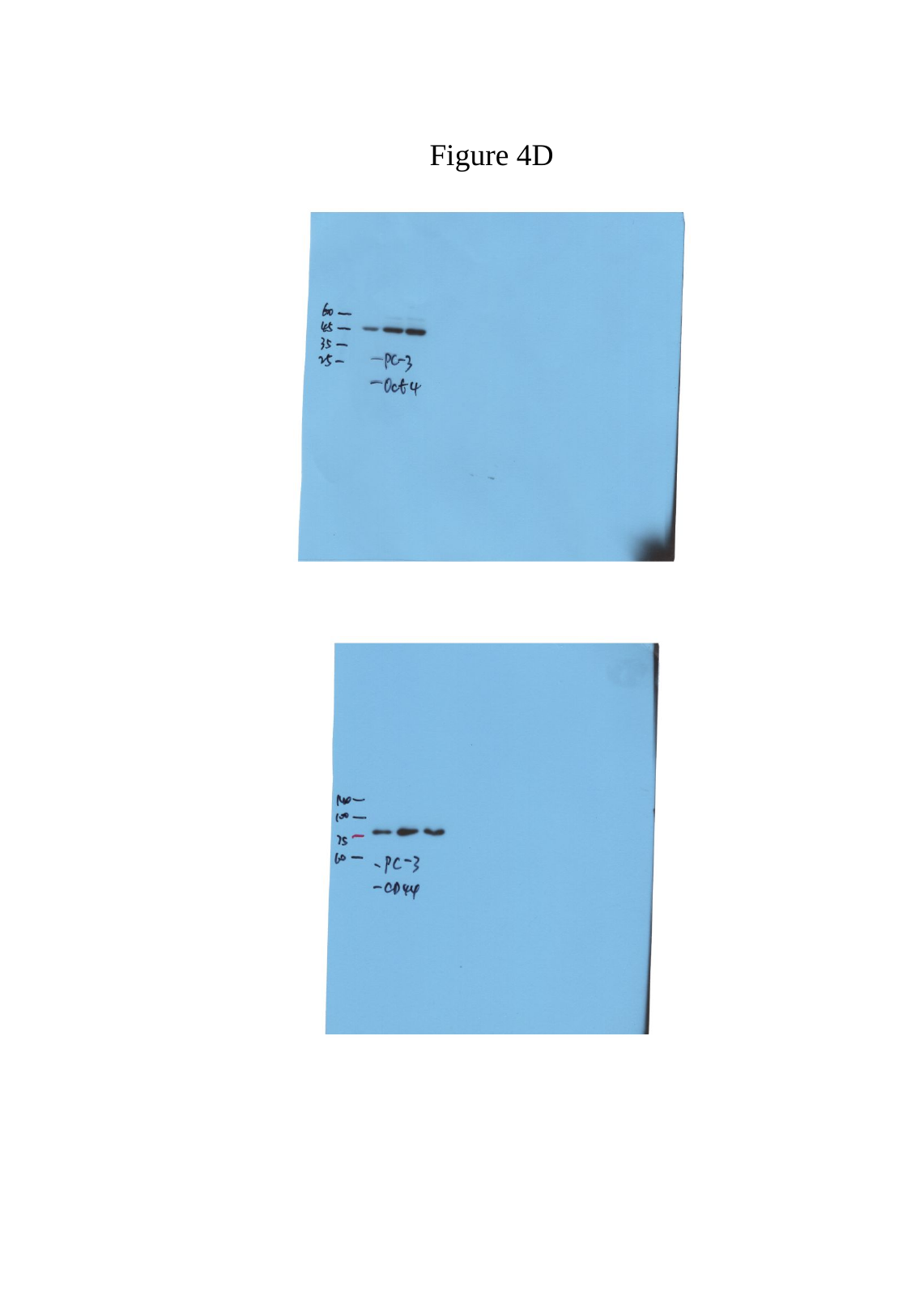

Figure 4D

## Slide 24
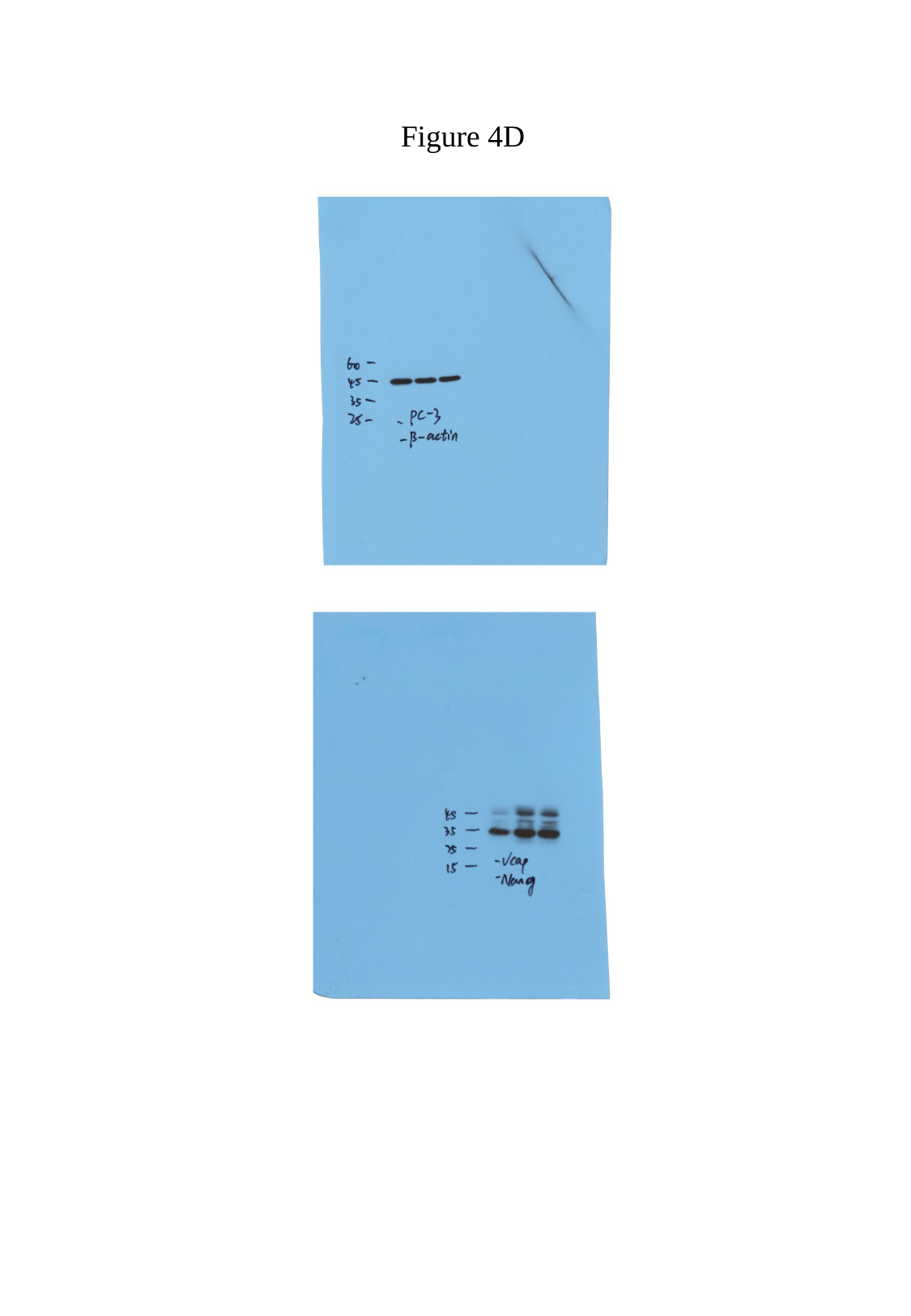

Figure 4D

## Slide 25
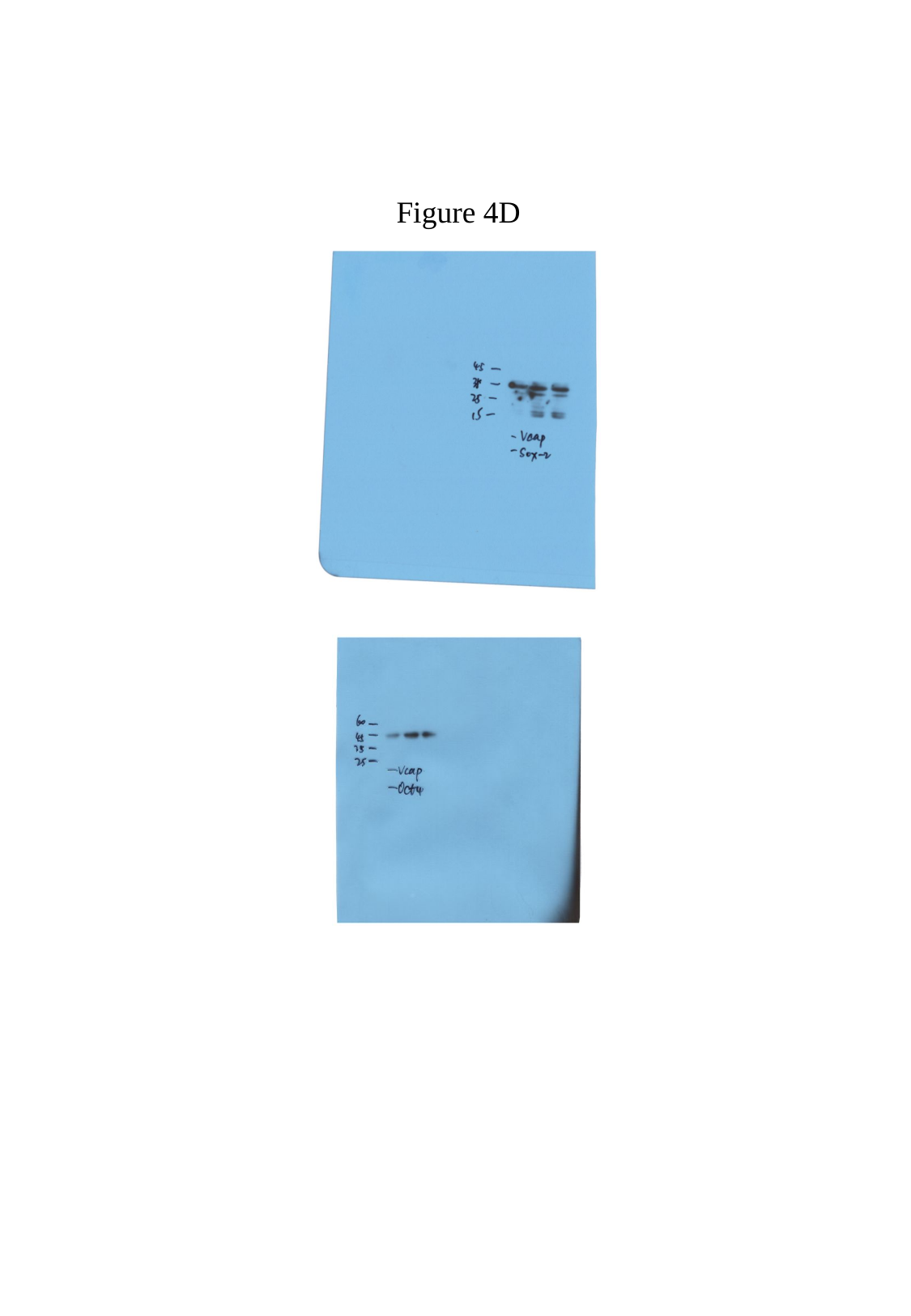

Figure 4D

## Slide 26
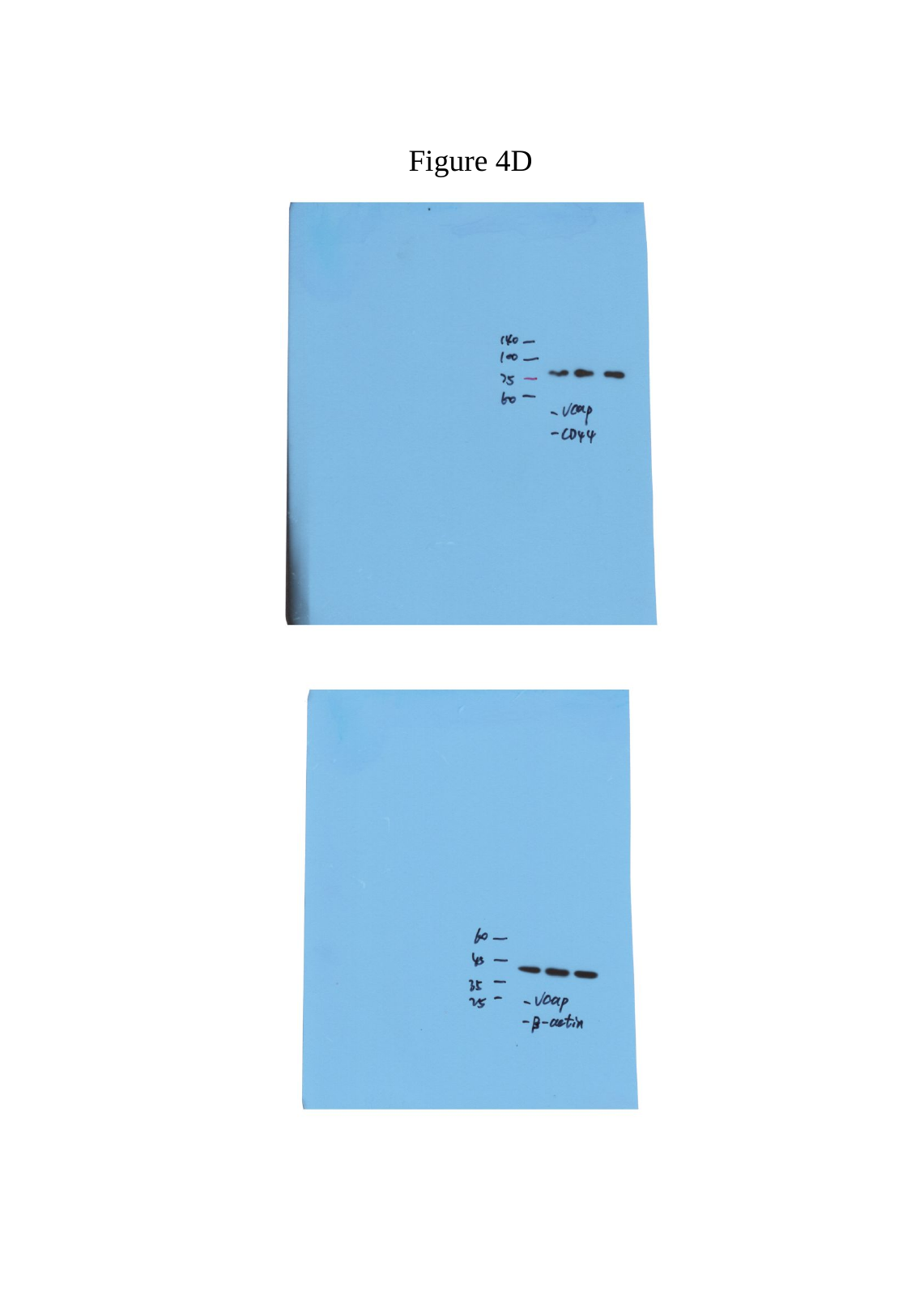

Figure 4D

## Slide 27
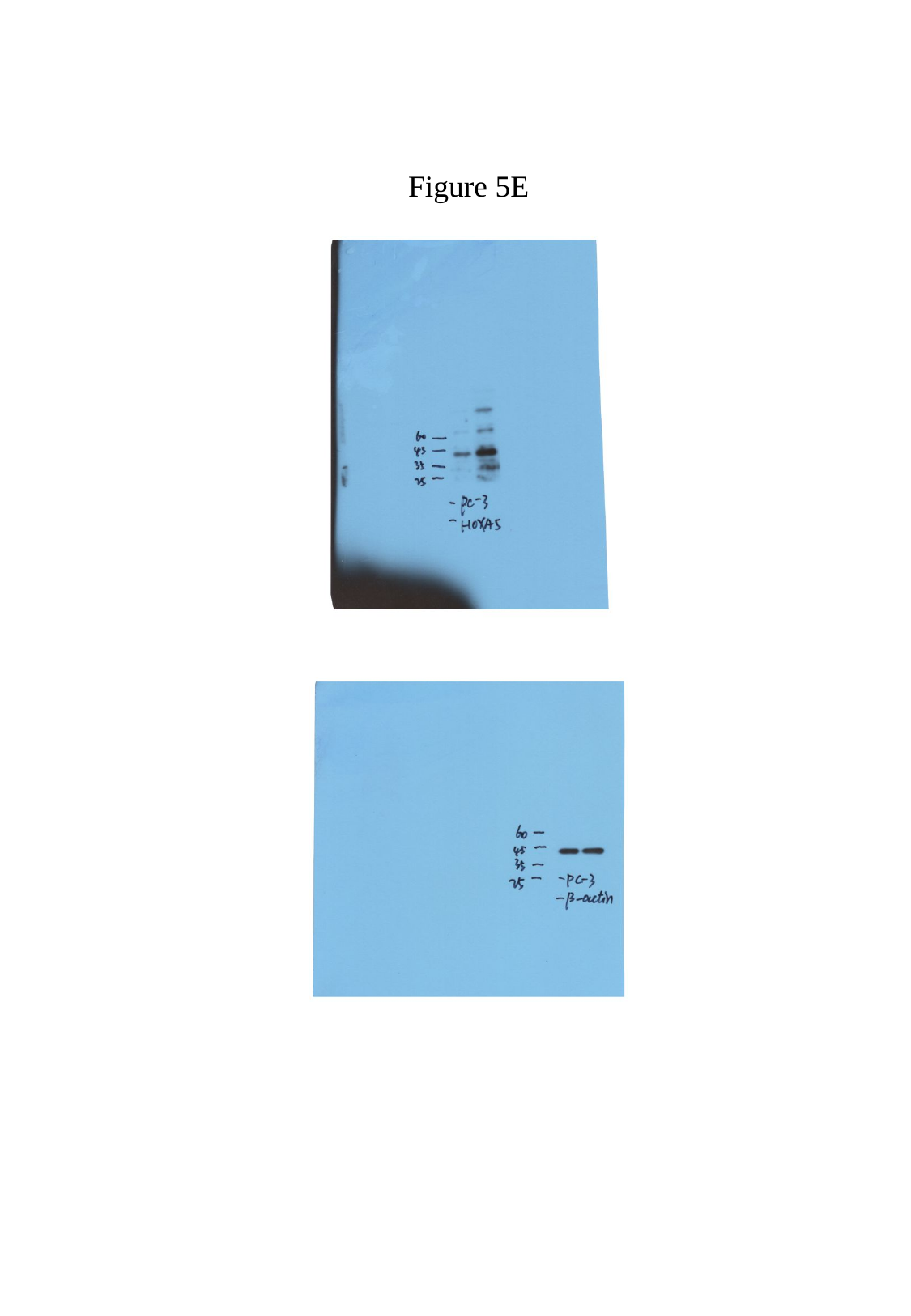

Figure 5E

## Slide 28
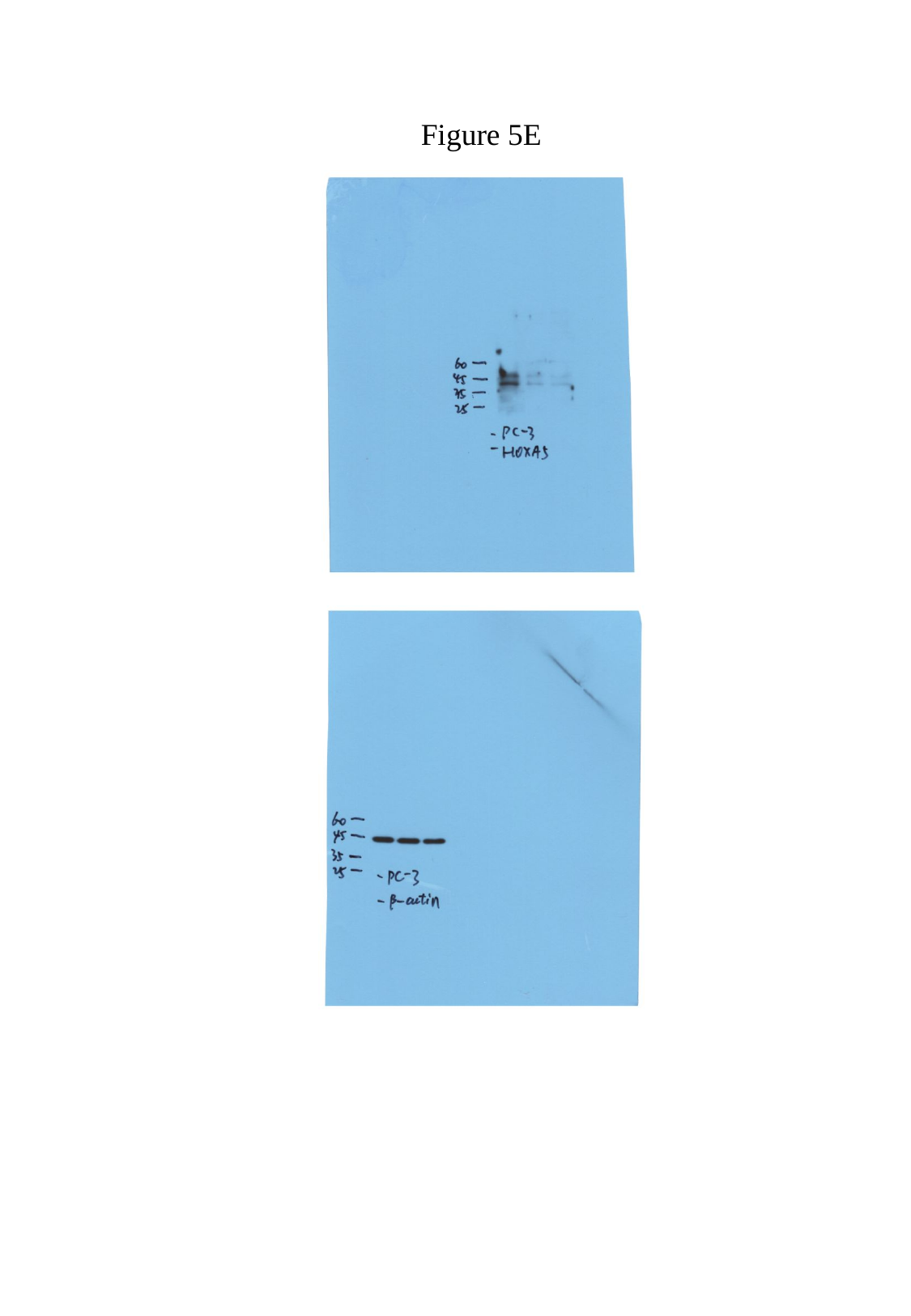

Figure 5E

## Slide 29
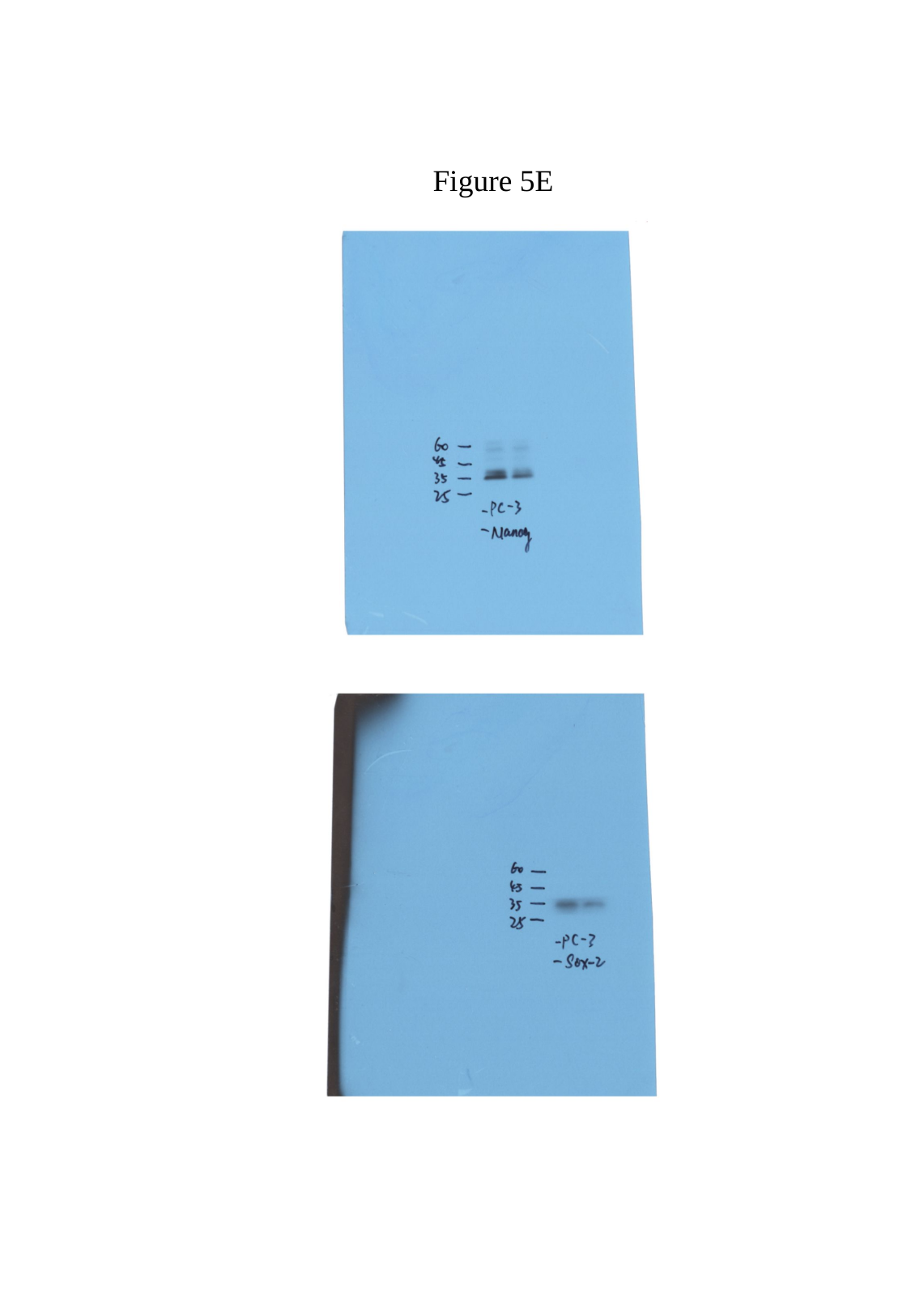

Figure 5E

## Slide 30
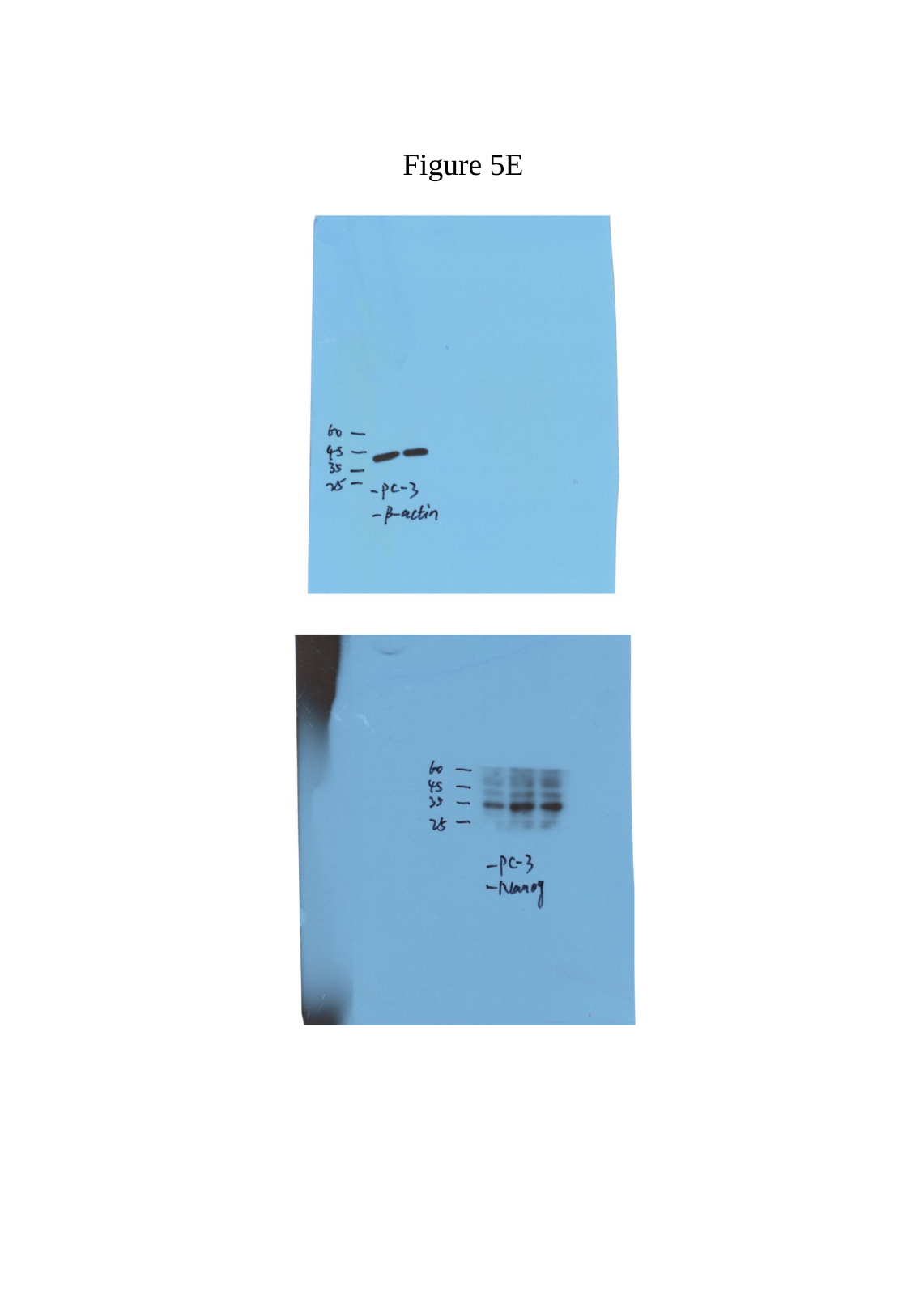

Figure 5E

## Slide 31
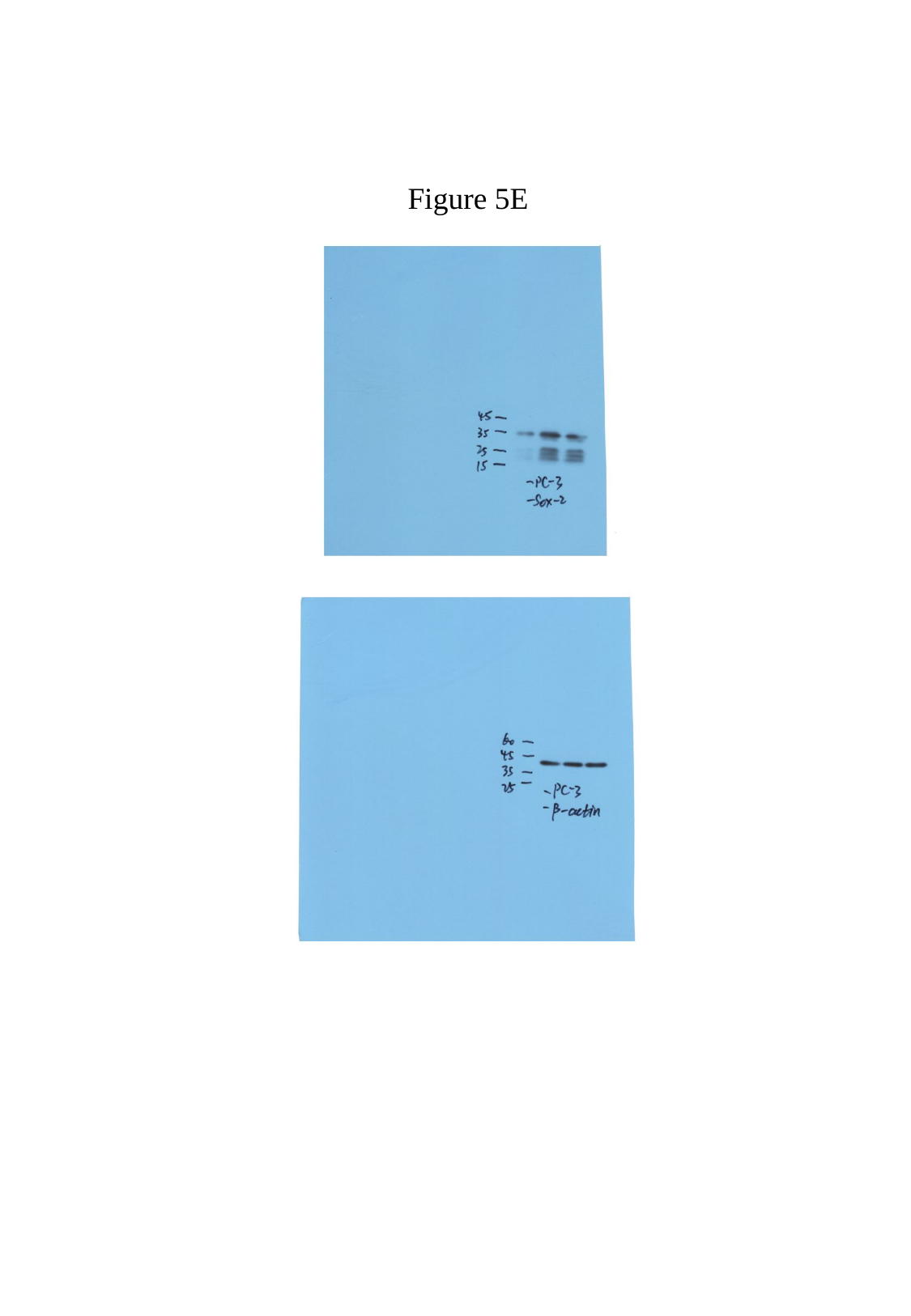

Figure 5E

## Slide 32
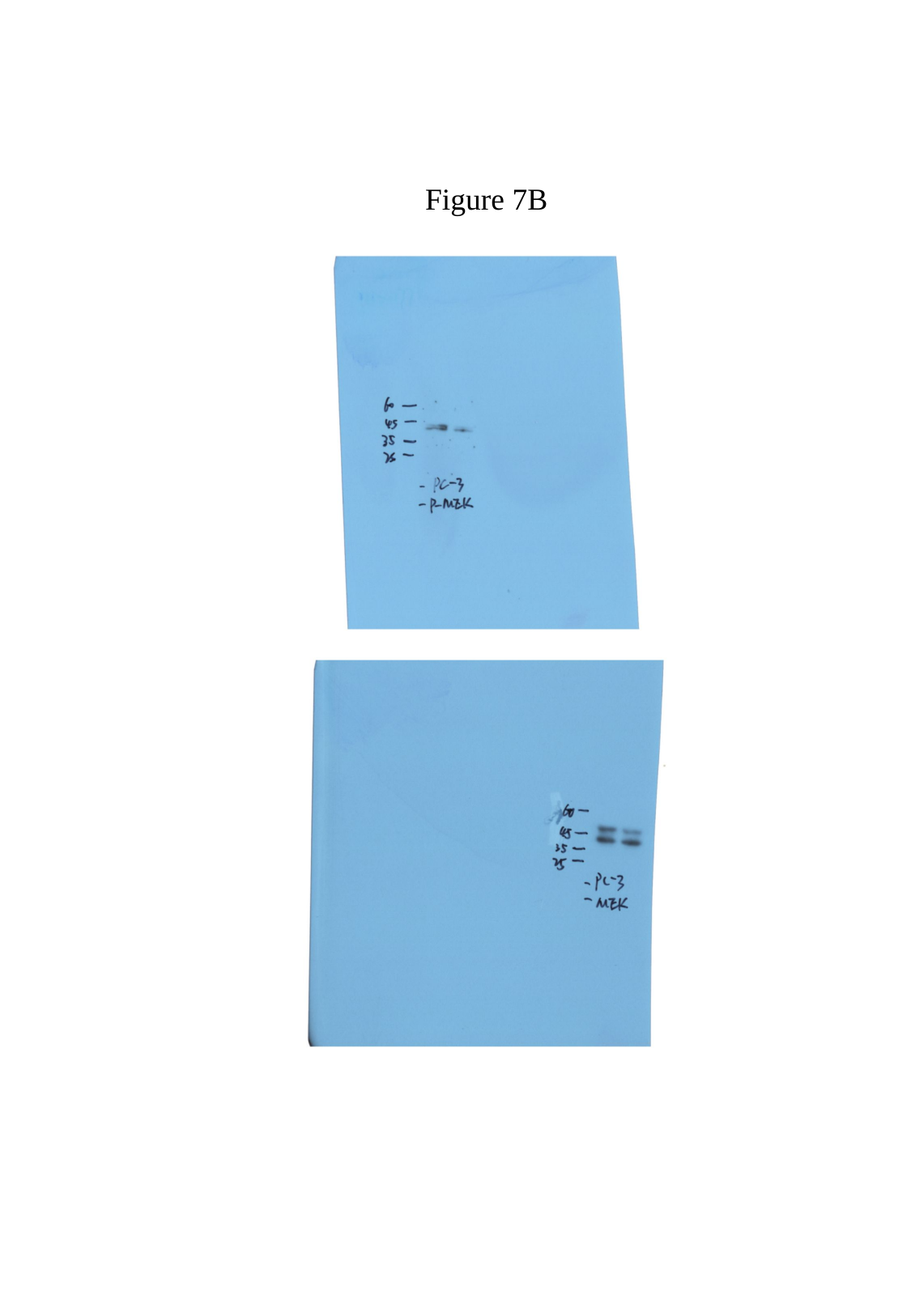

Figure 7B

## Slide 33
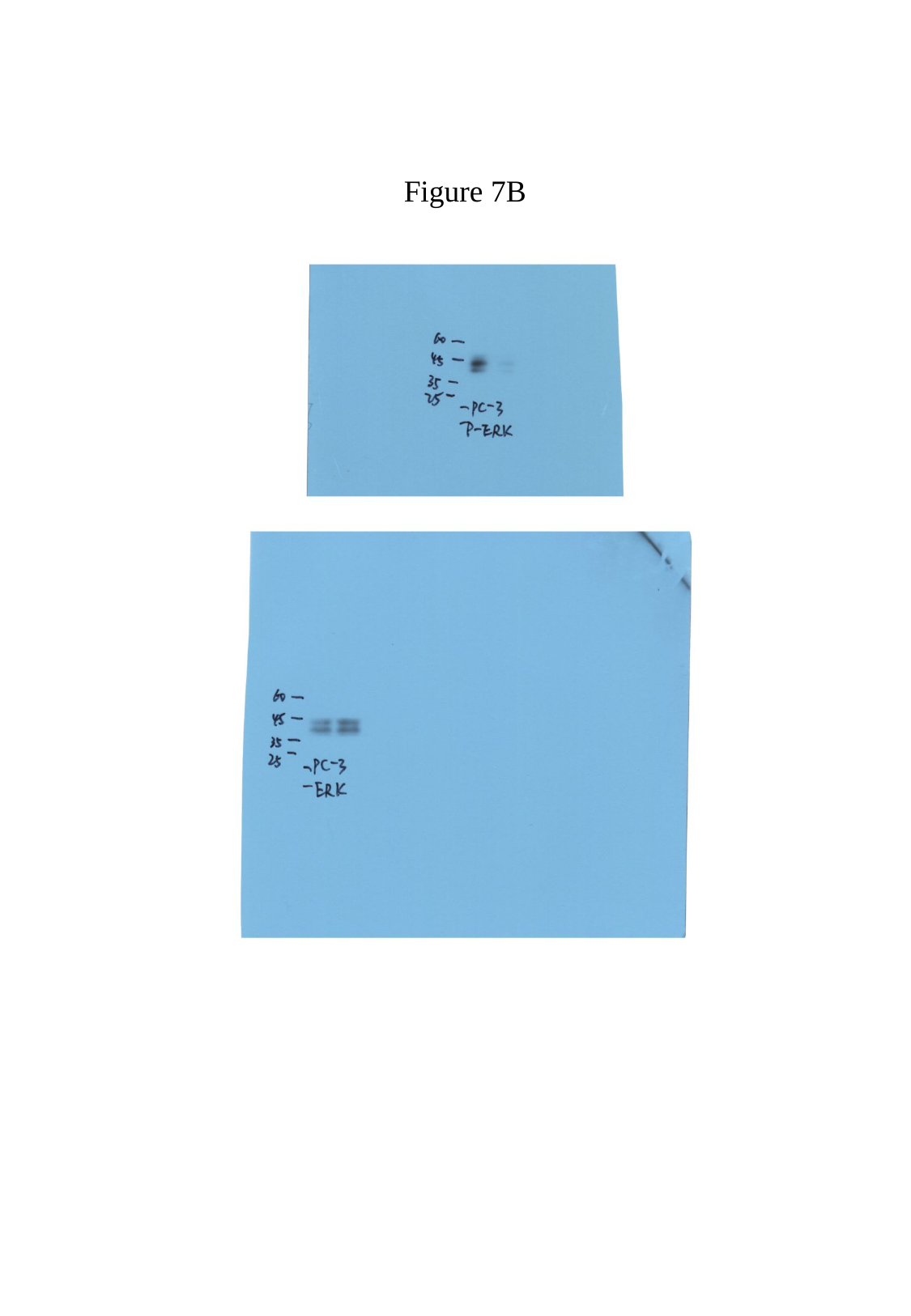

Figure 7B

## Slide 34
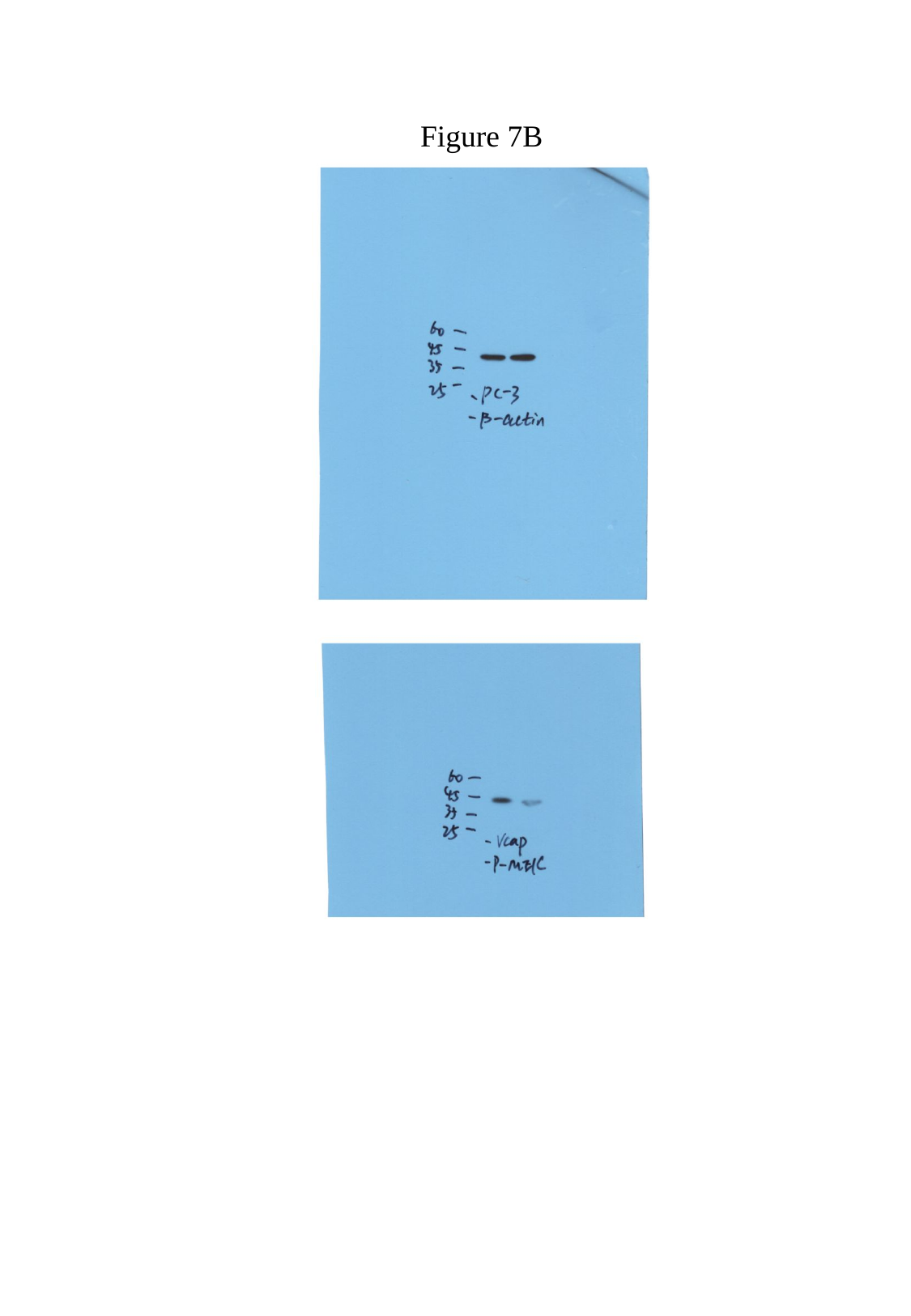

Figure 7B

## Slide 35
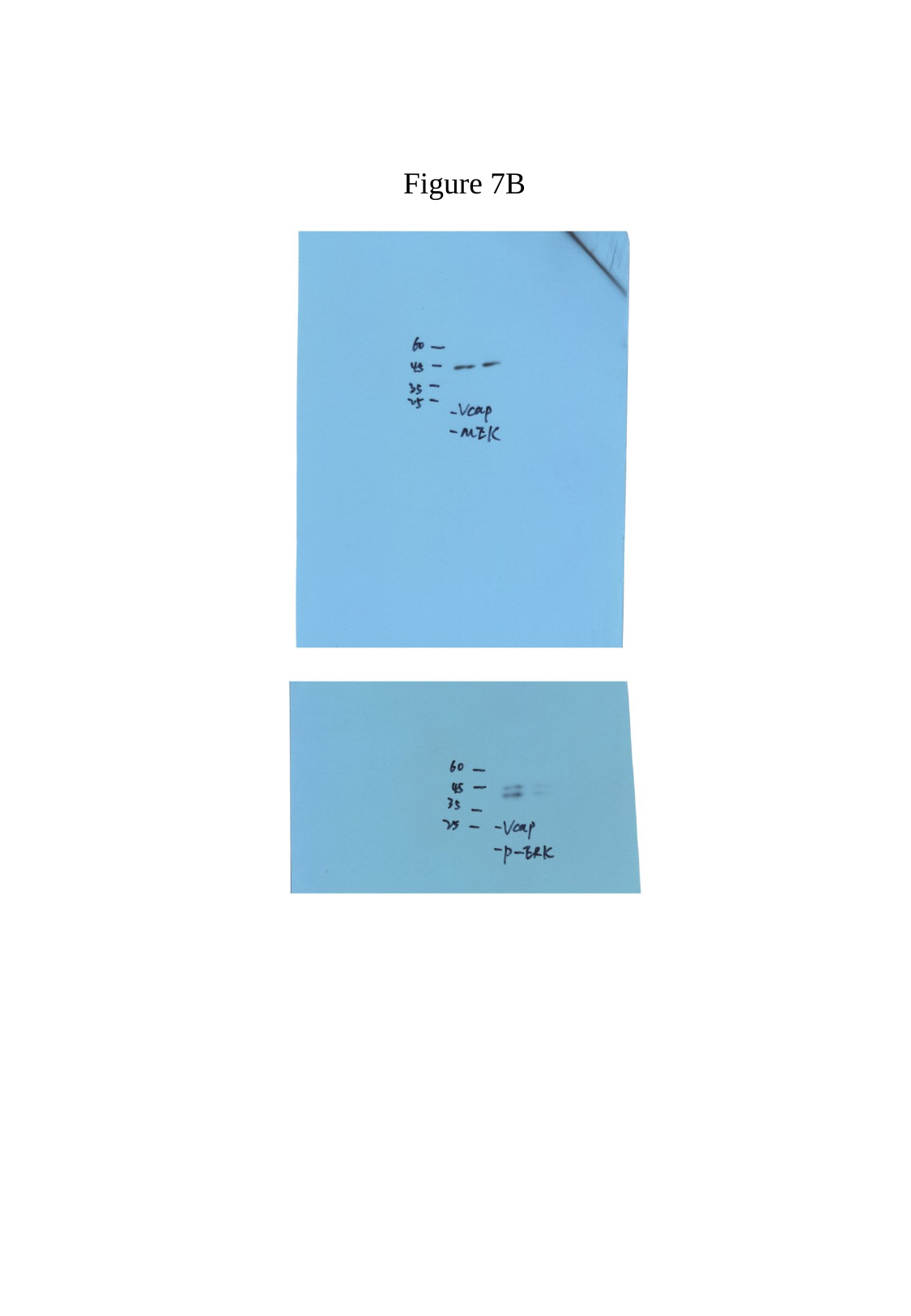

Figure 7B

## Slide 36
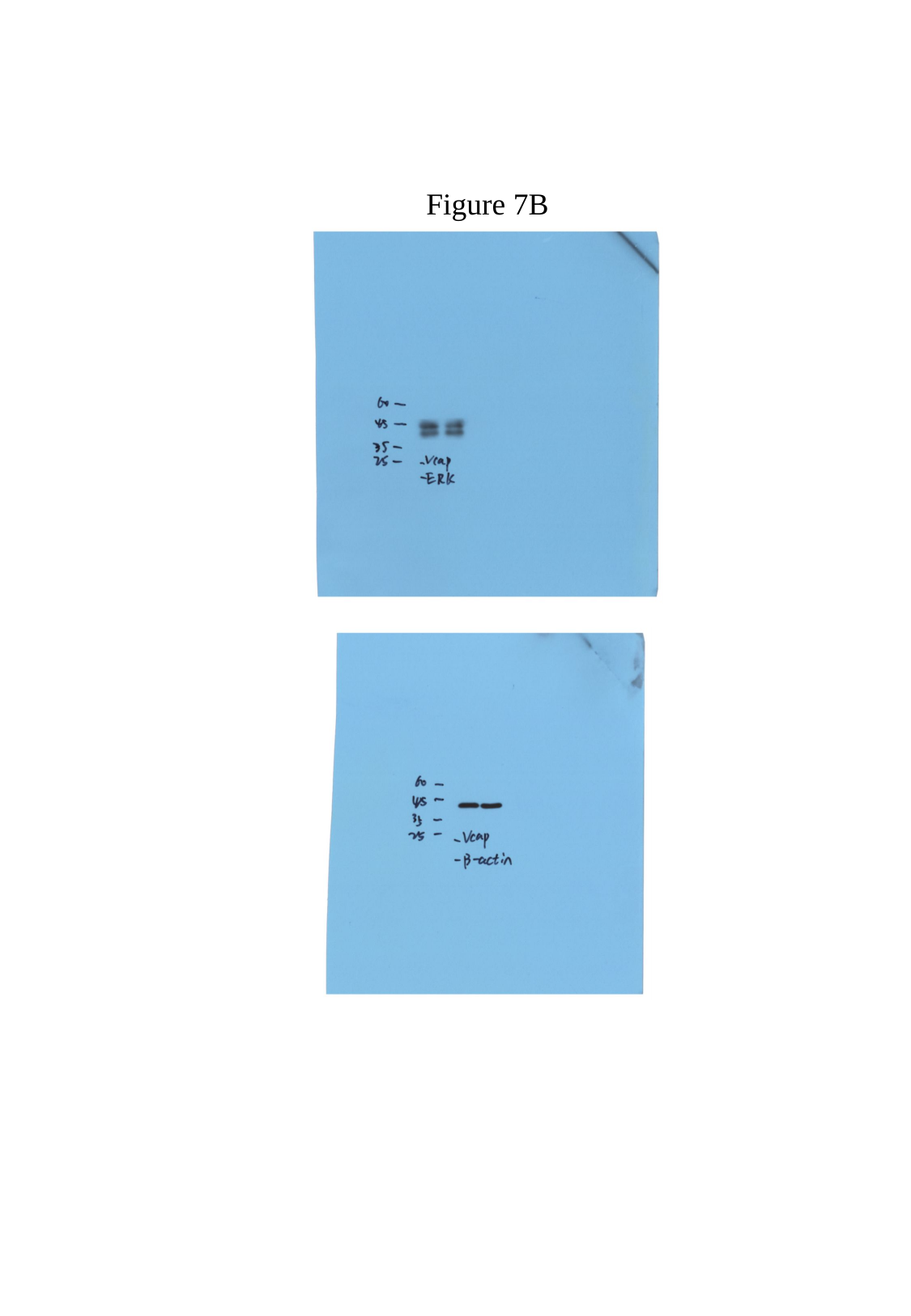

Figure 7B

## Slide 37
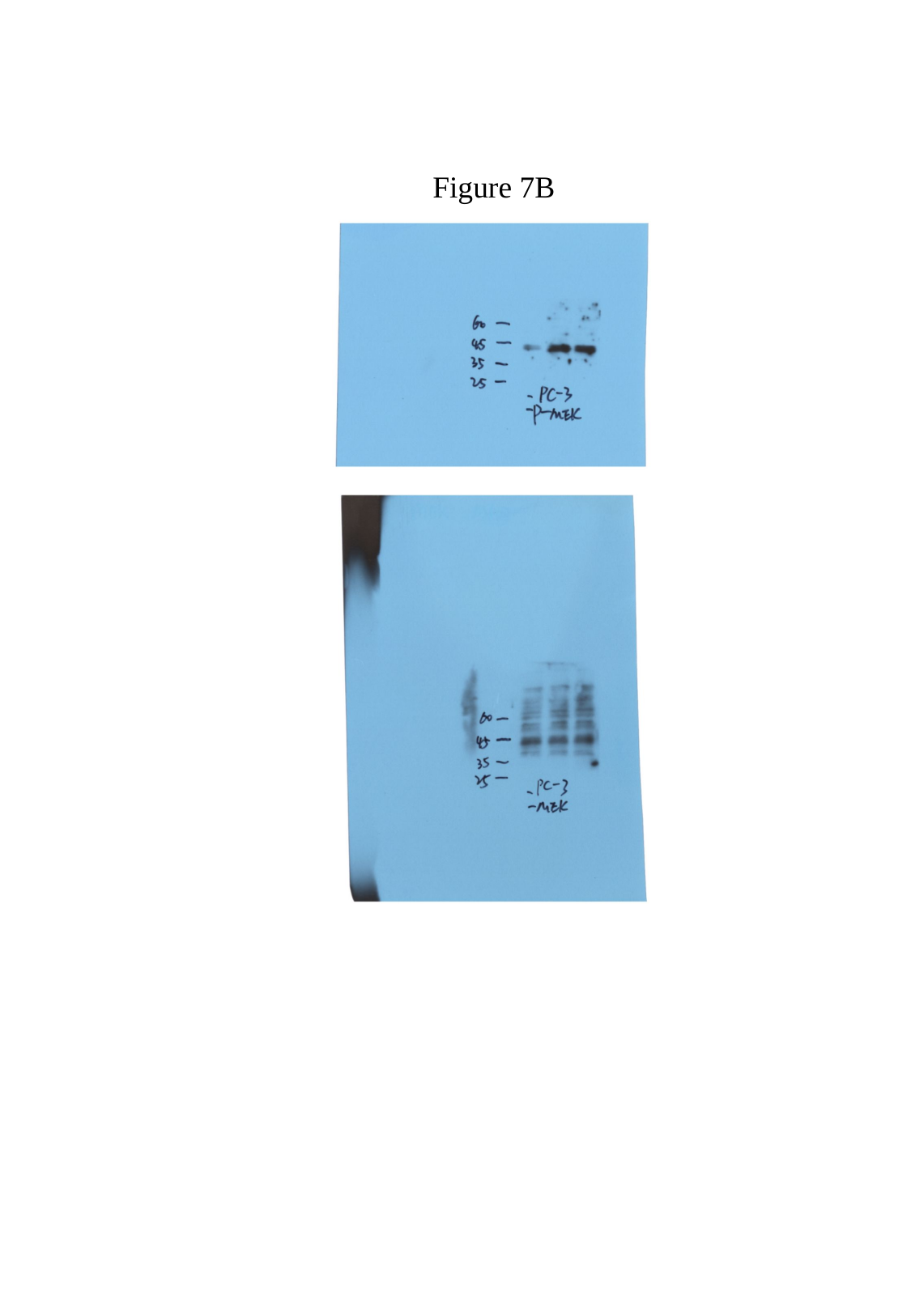

Figure 7B

## Slide 38
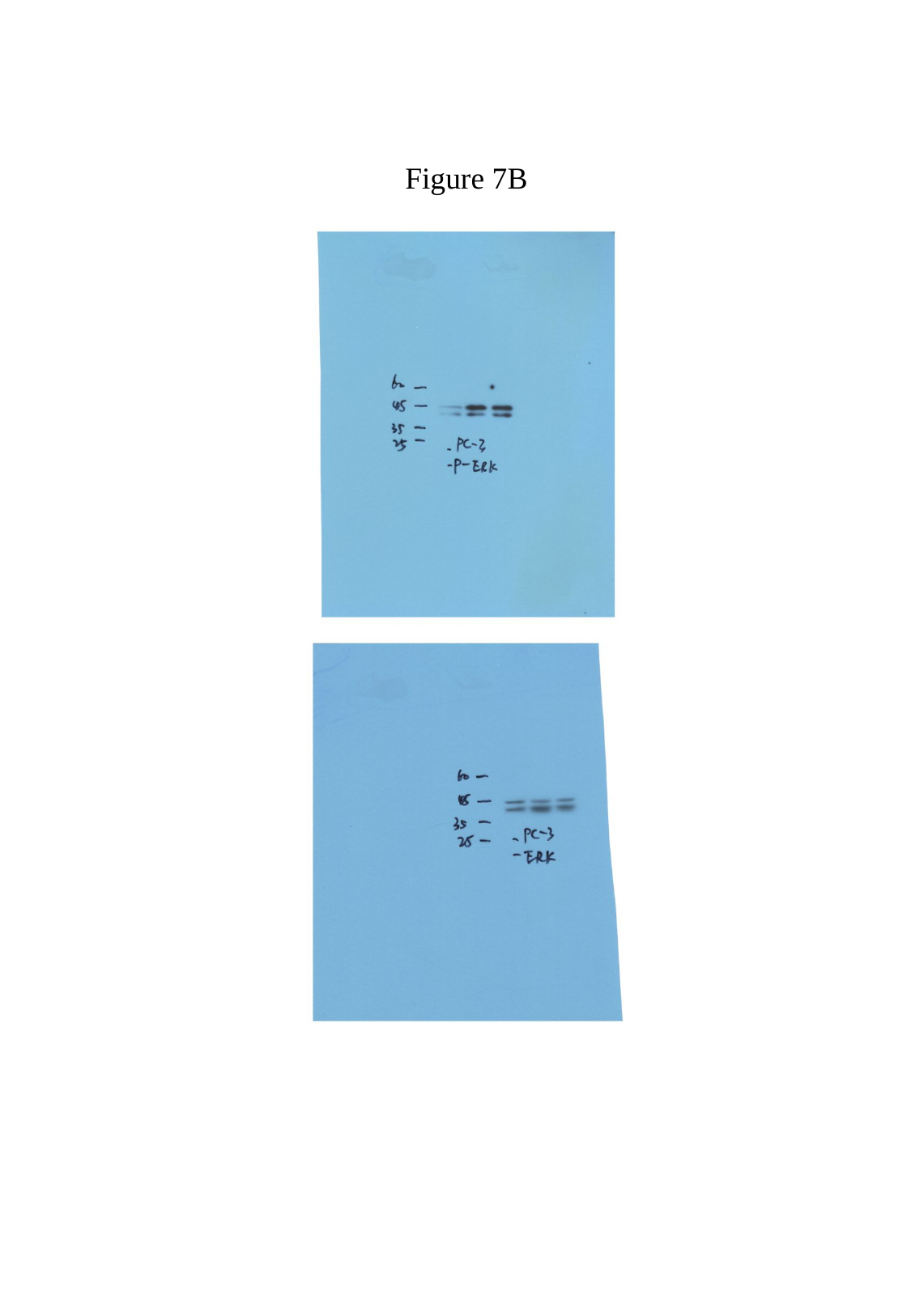

Figure 7B

## Slide 39
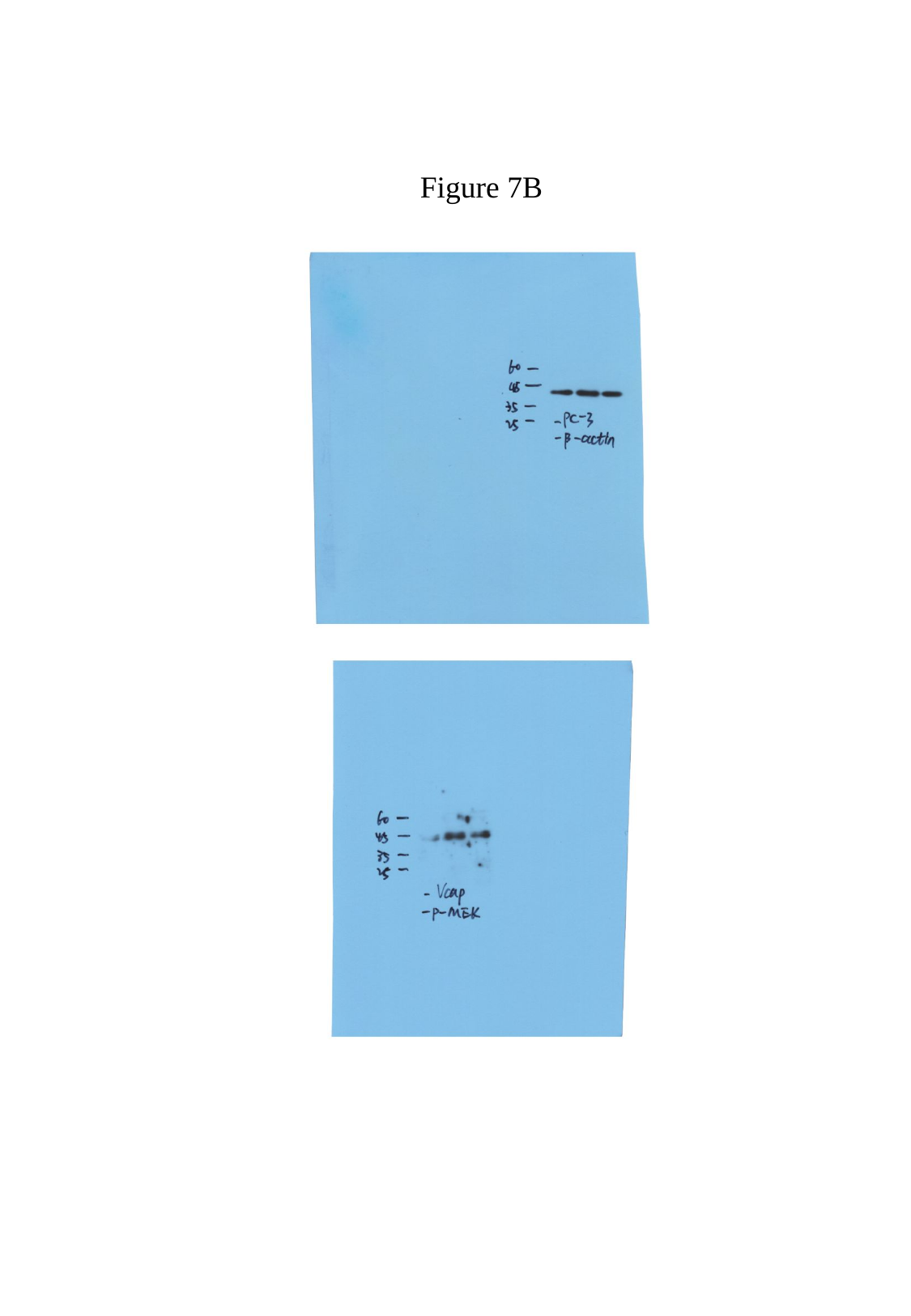

Figure 7B

## Slide 40
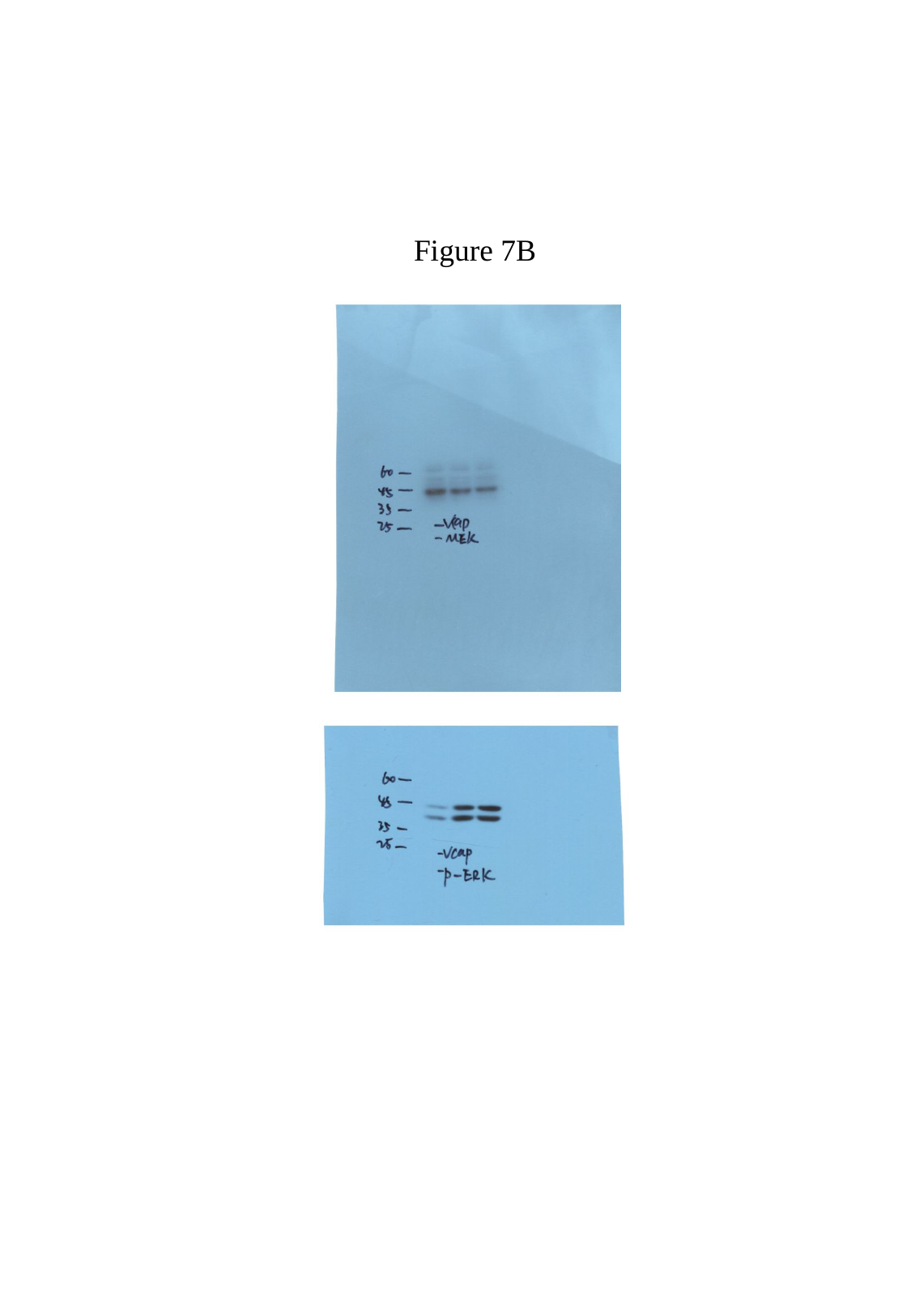

Figure 7B

## Slide 41
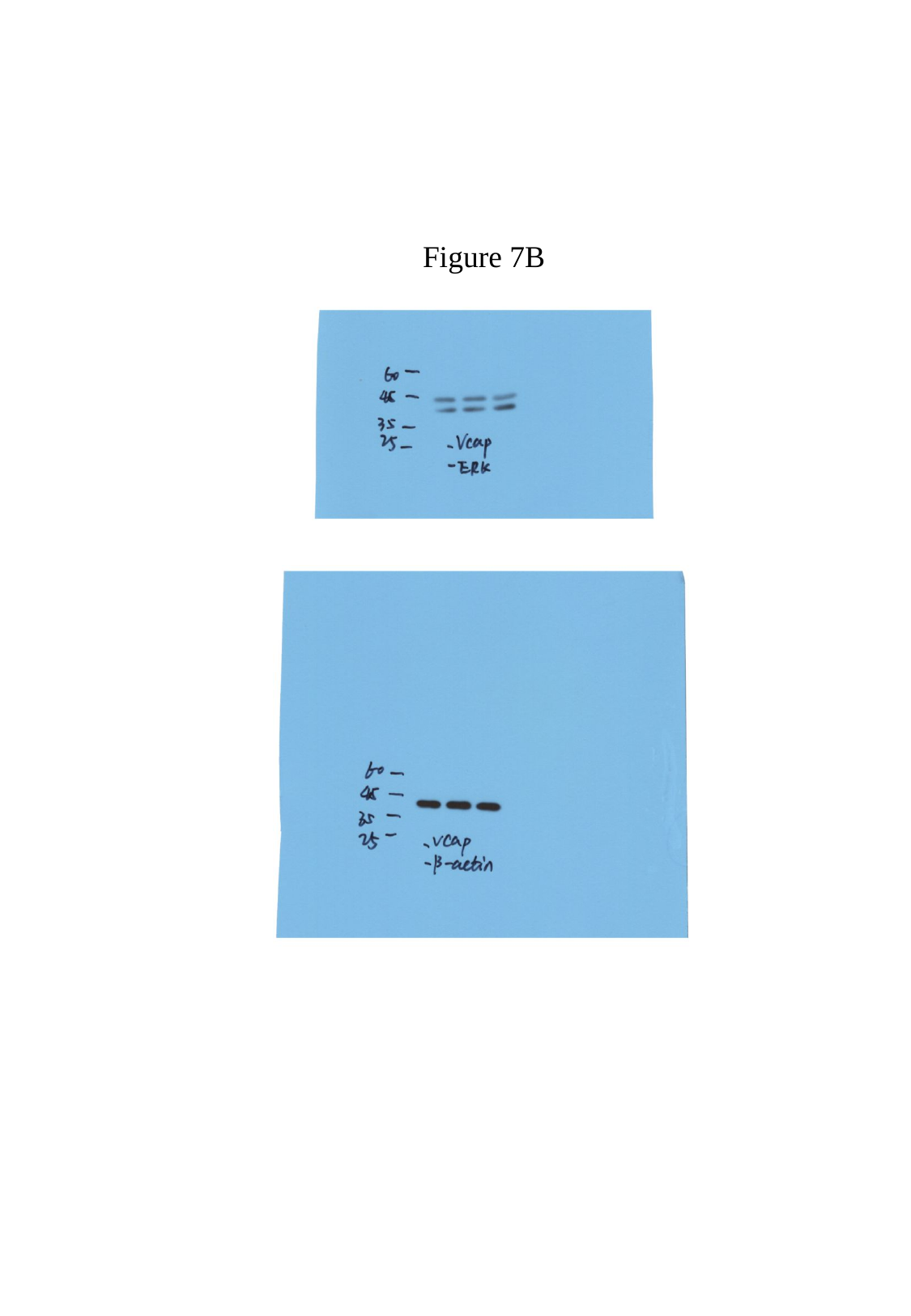

Figure 7B

## Slide 42
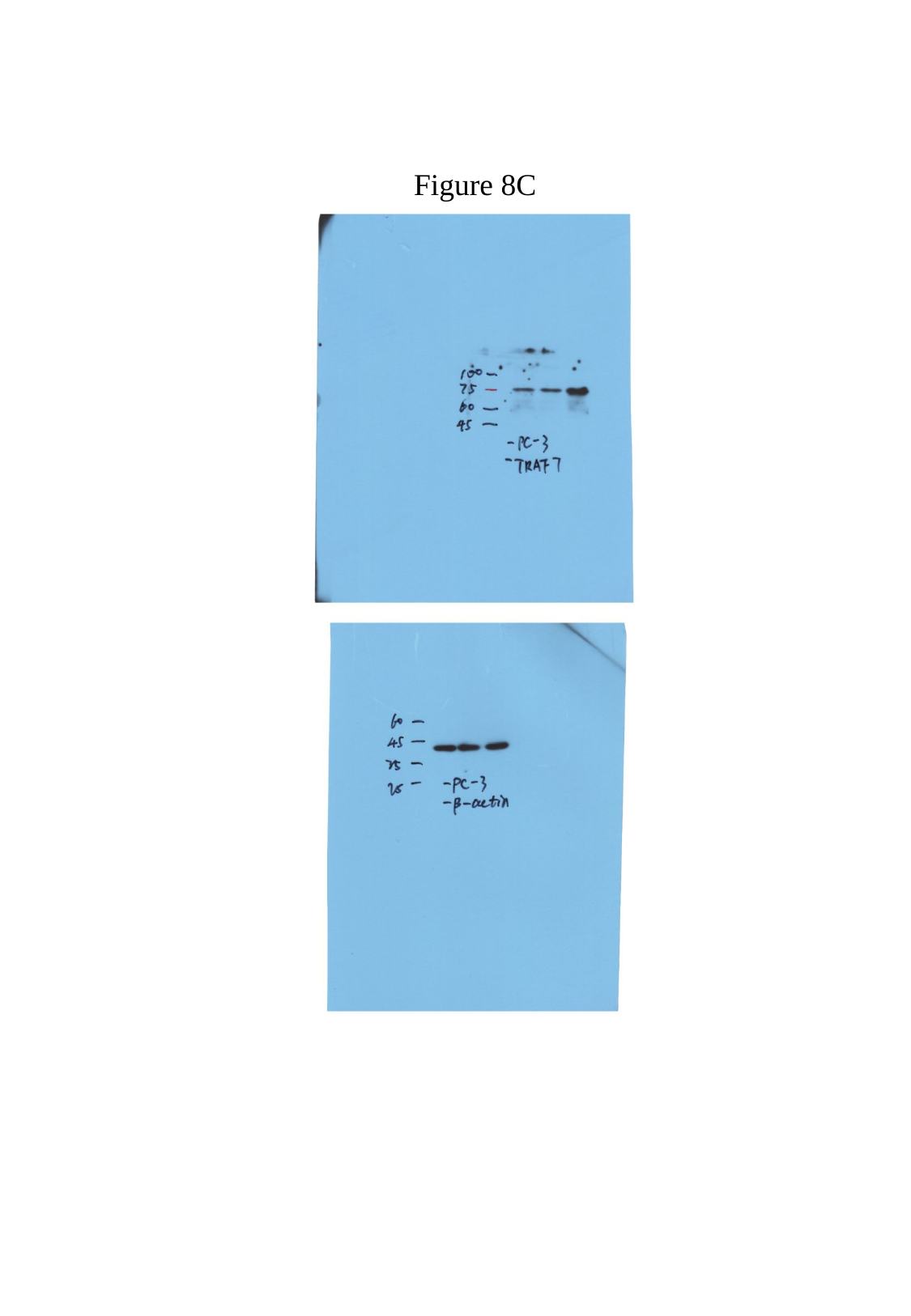

Figure 8C

## Slide 43
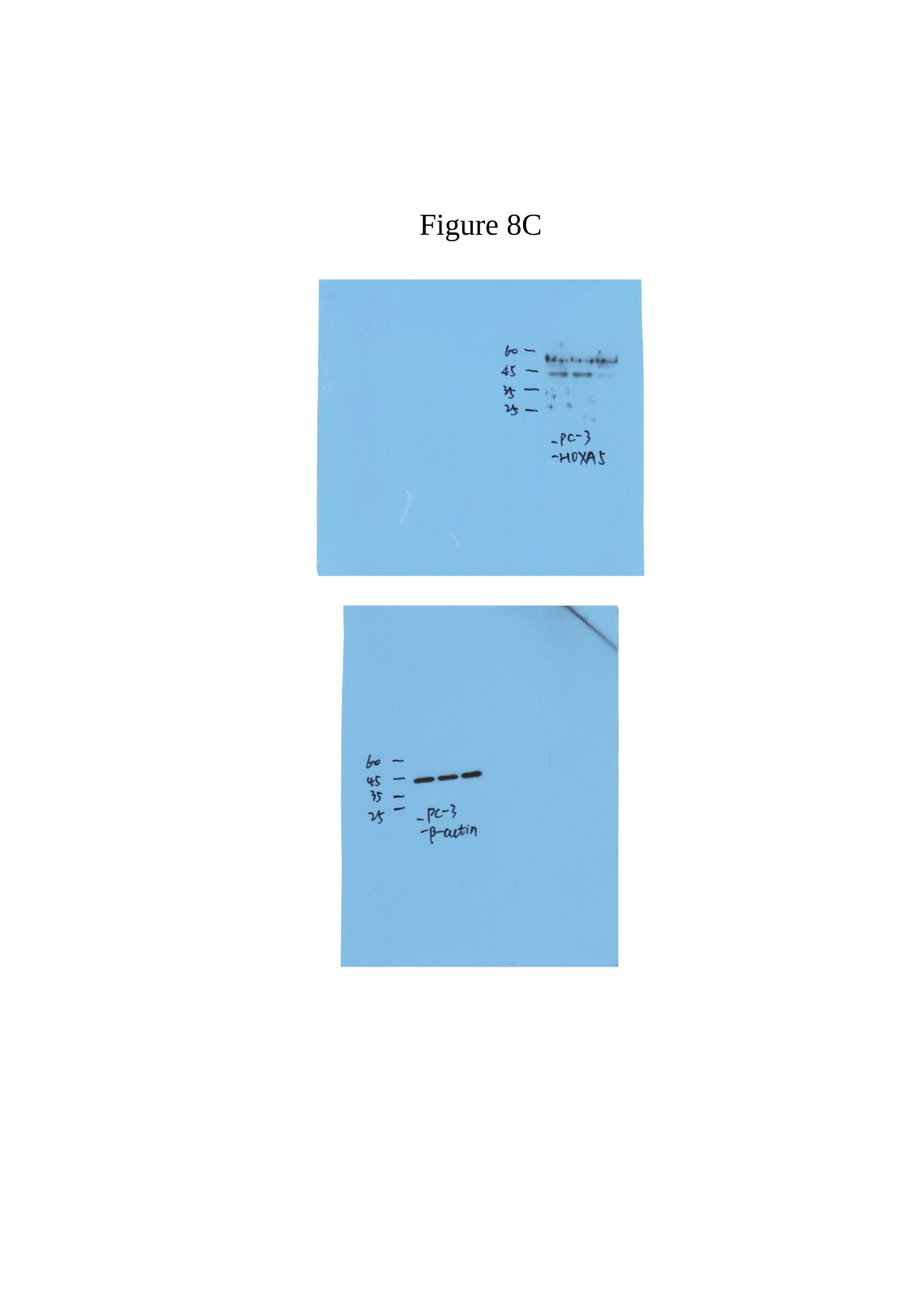

Figure 8C

## Slide 44
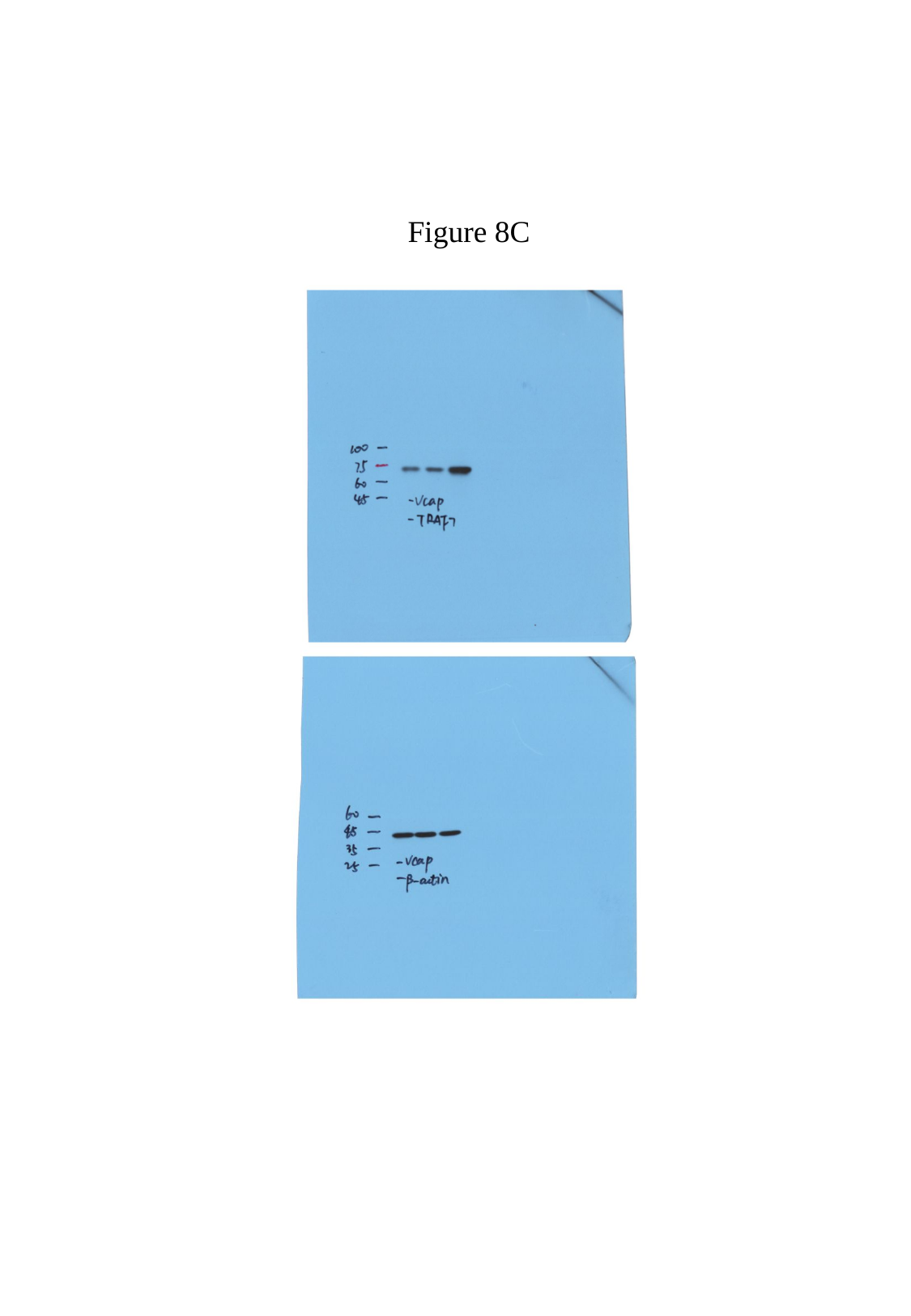

Figure 8C

## Slide 45
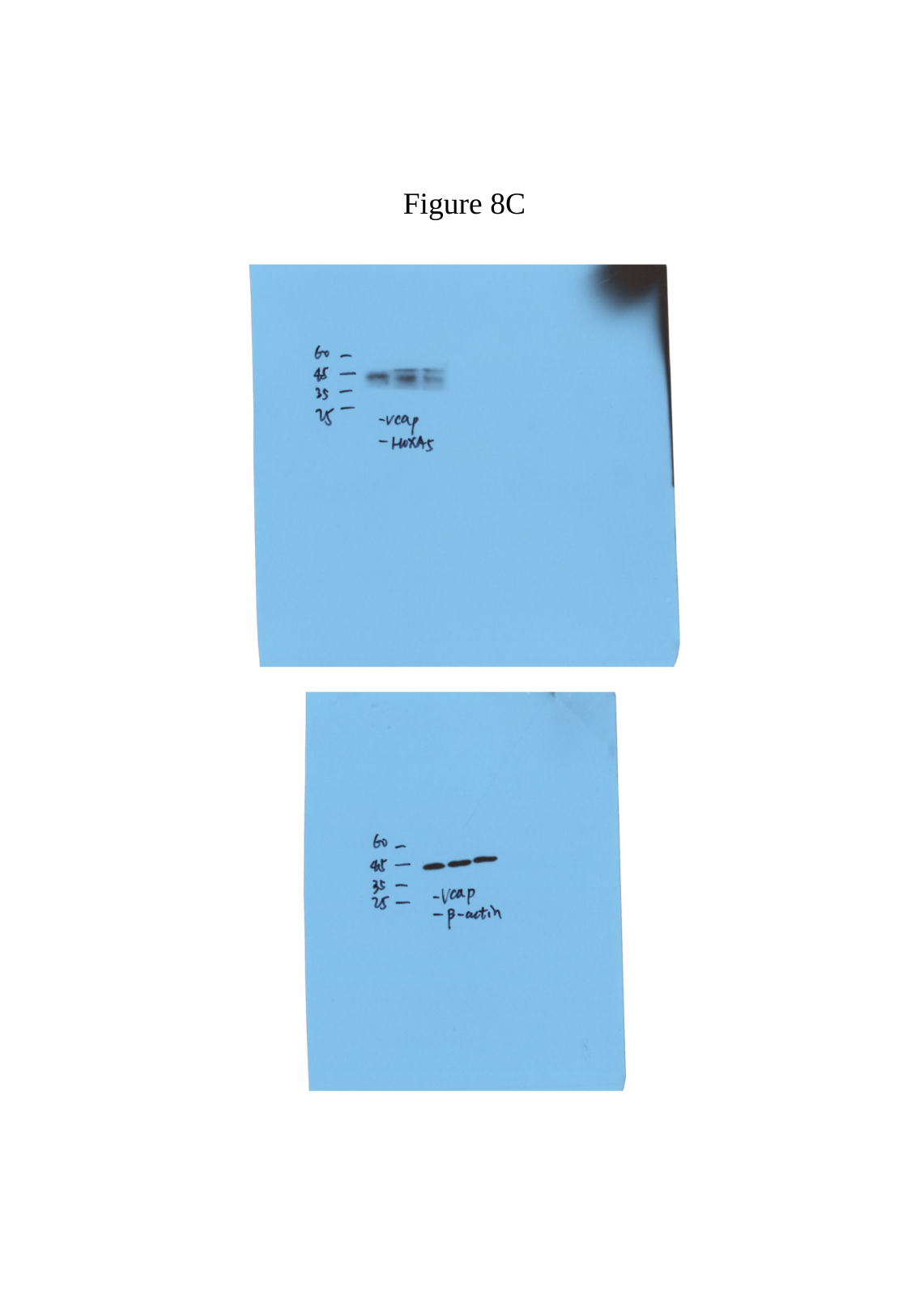

Figure 8C

## Slide 46
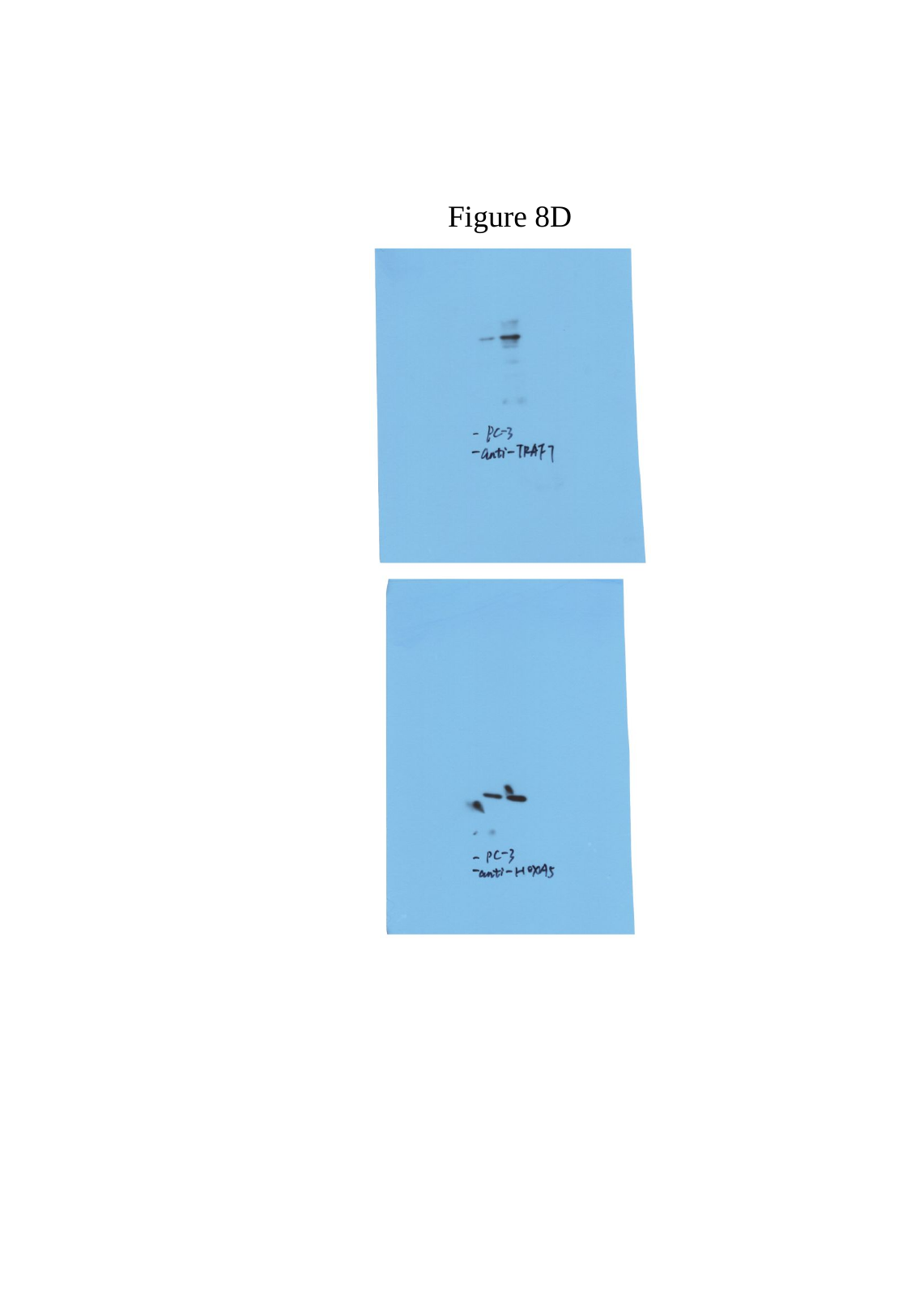

Figure 8D

## Slide 47
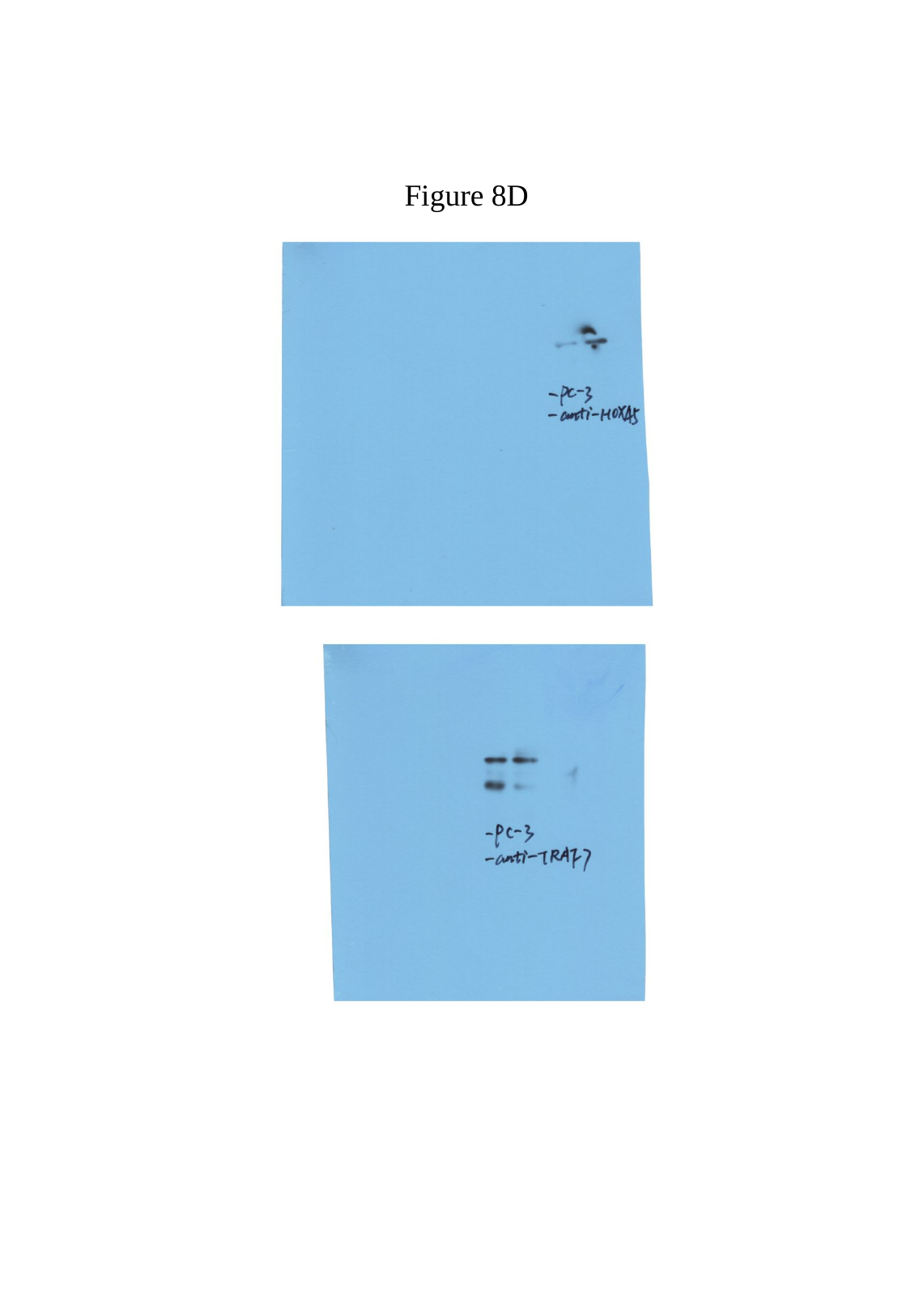

Figure 8D

## Slide 48
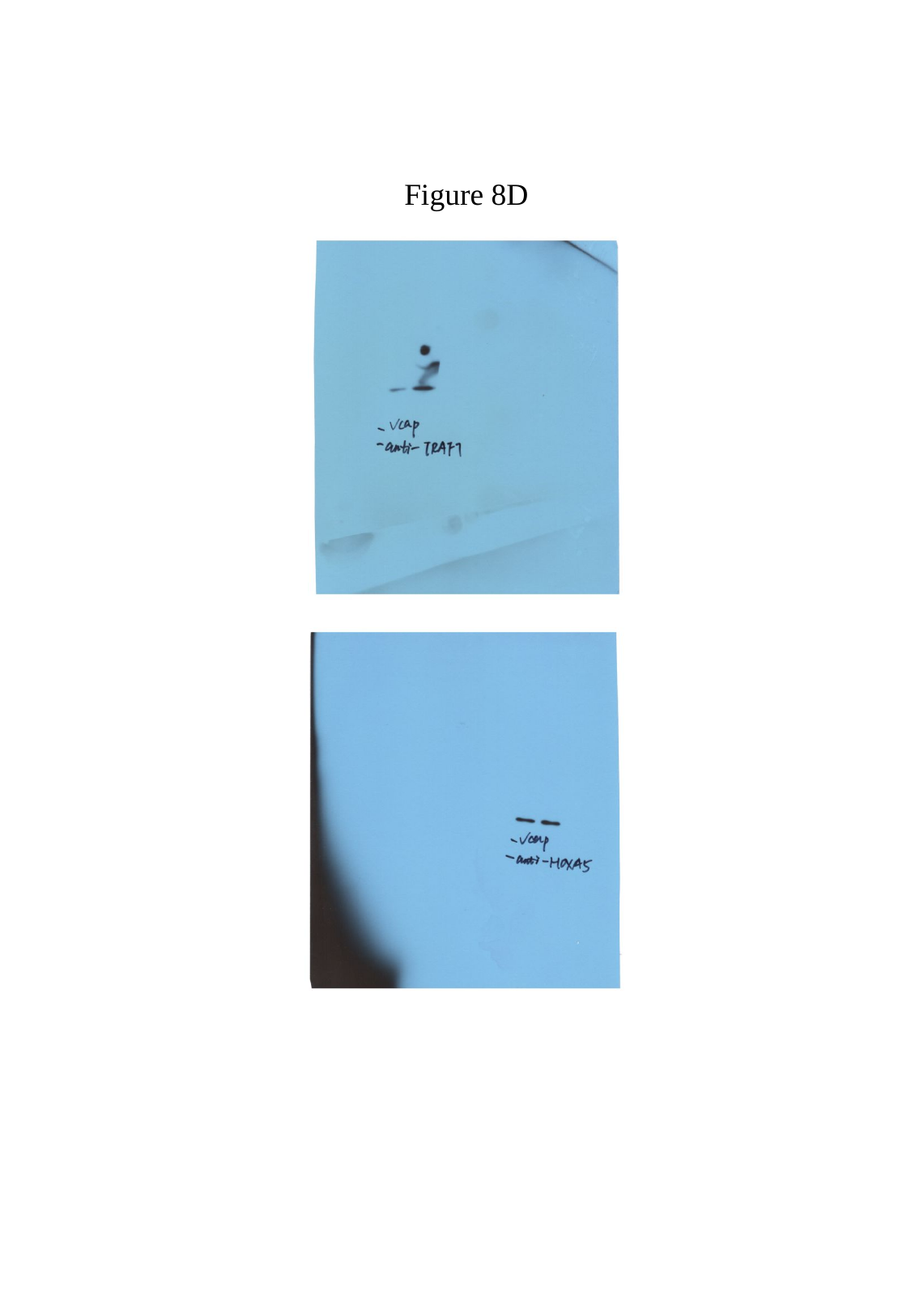

Figure 8D

## Slide 49
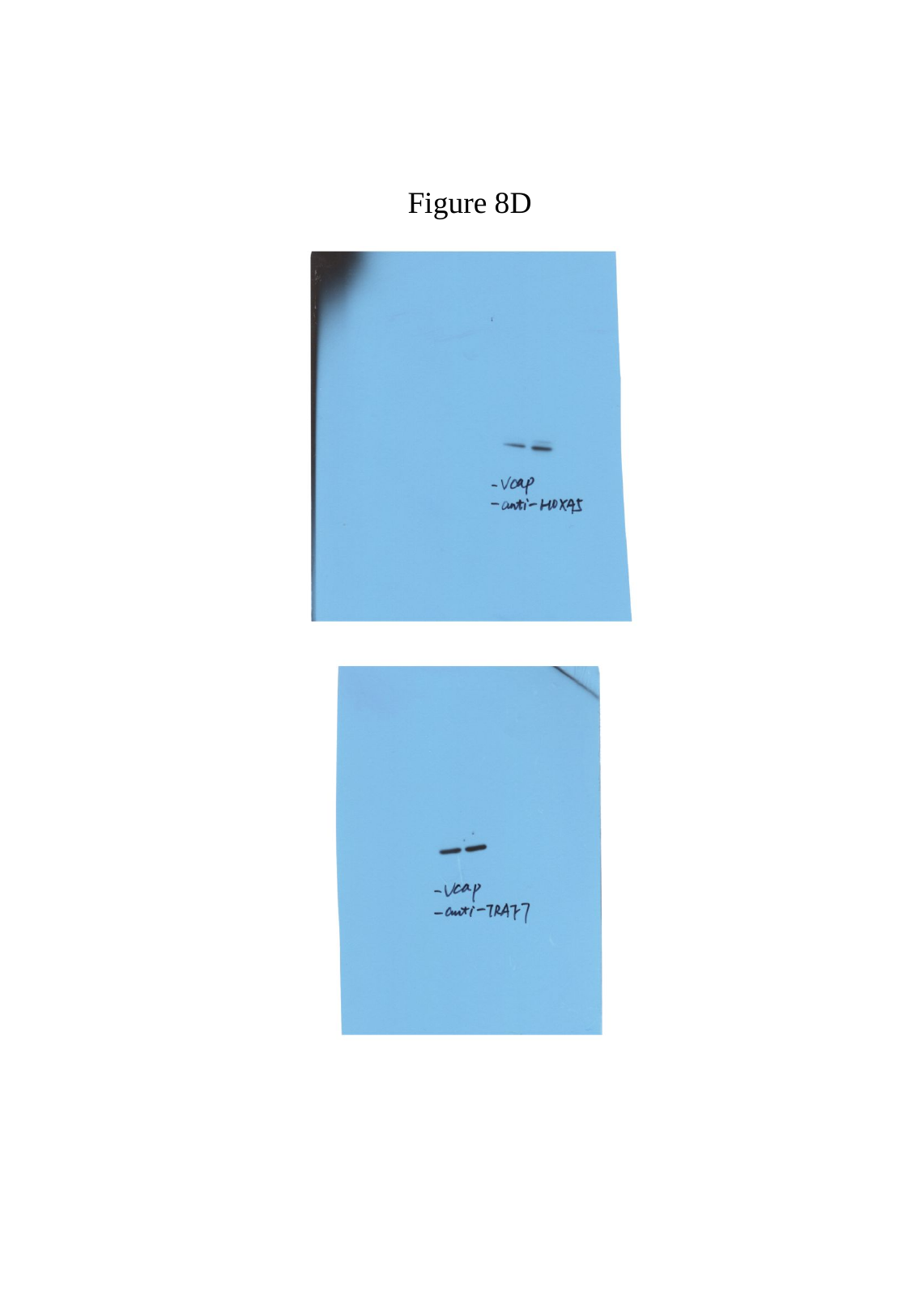

Figure 8D

## Slide 50
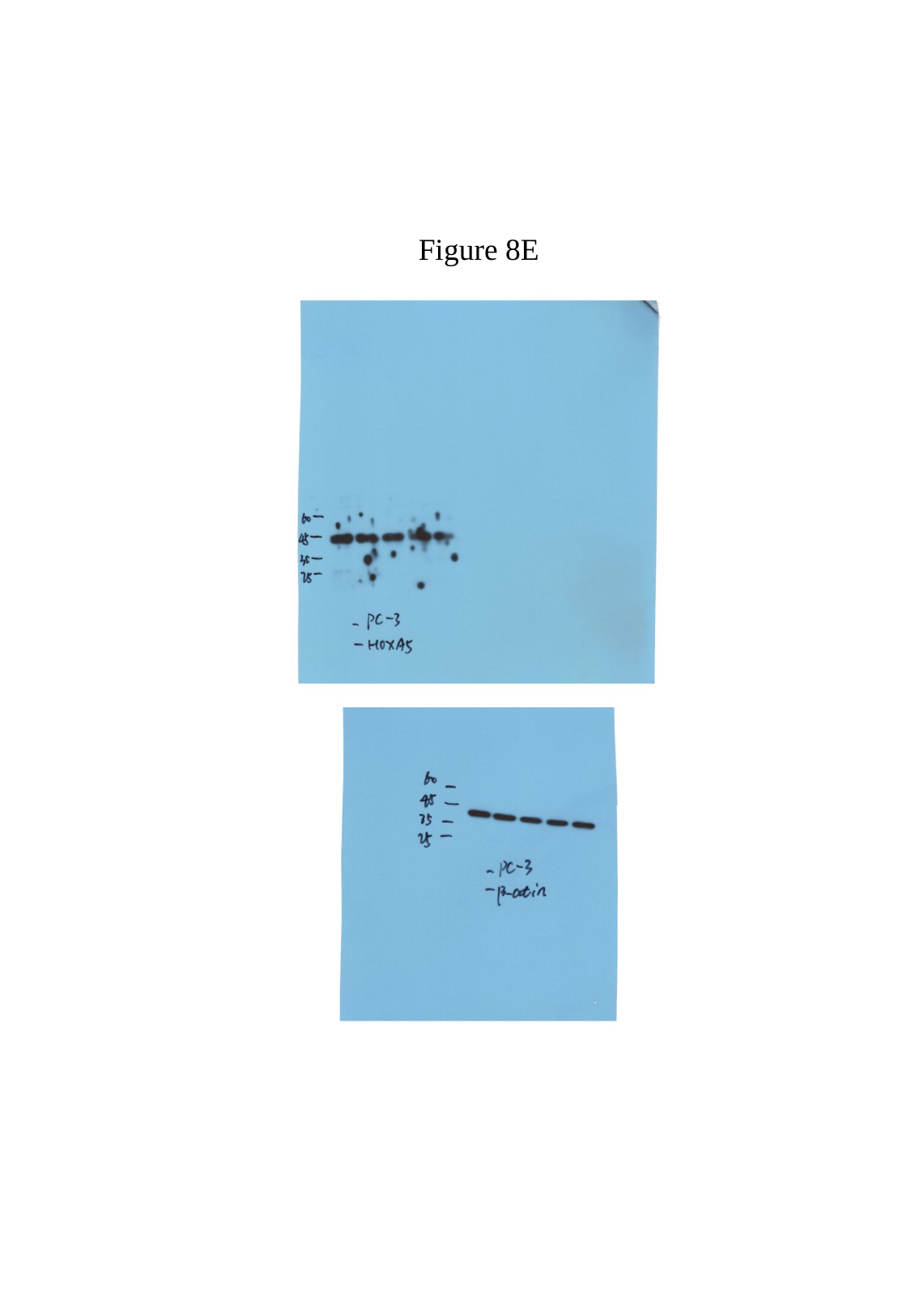

Figure 8E

## Slide 51
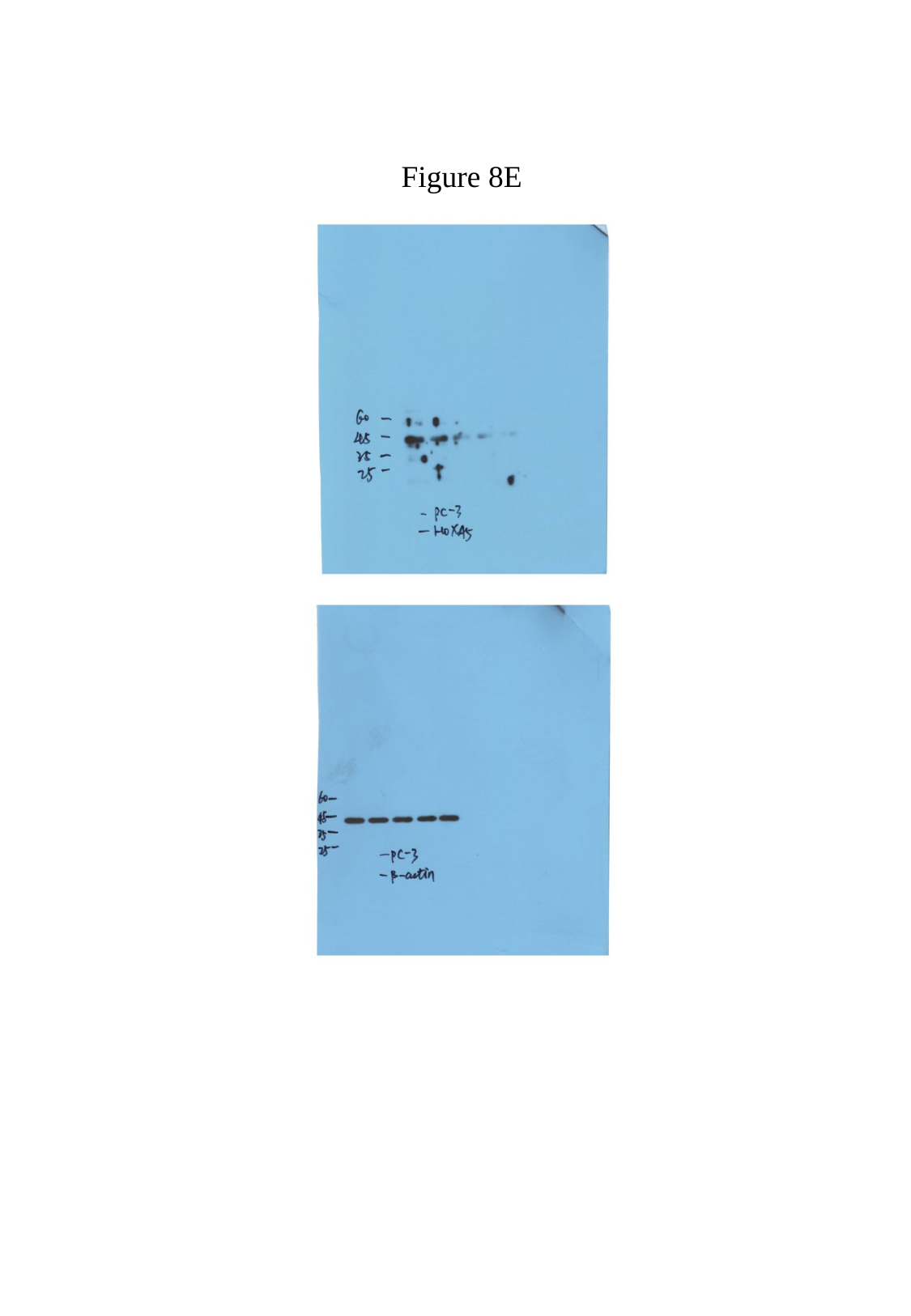

Figure 8E

## Slide 52
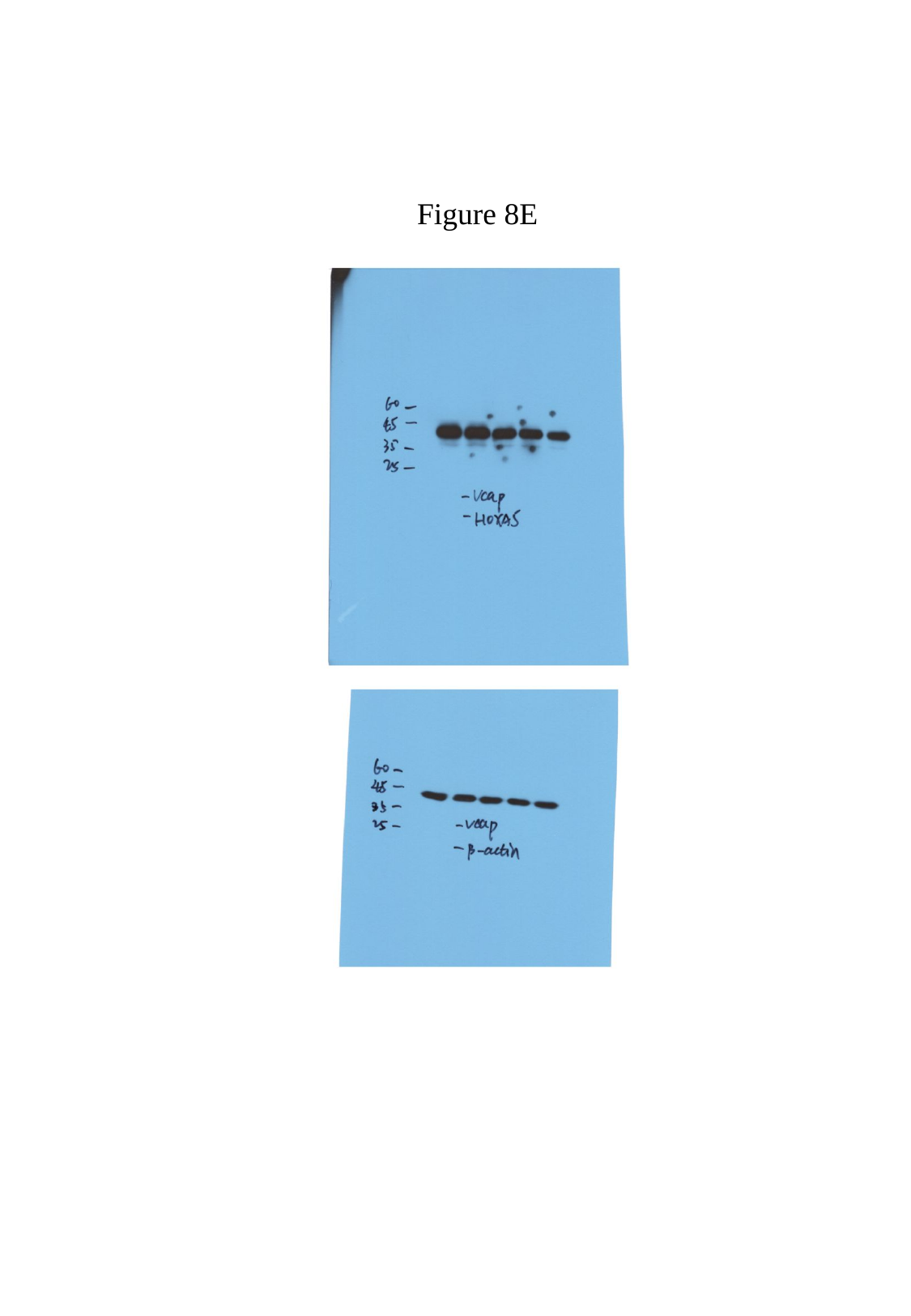

Figure 8E

## Slide 53
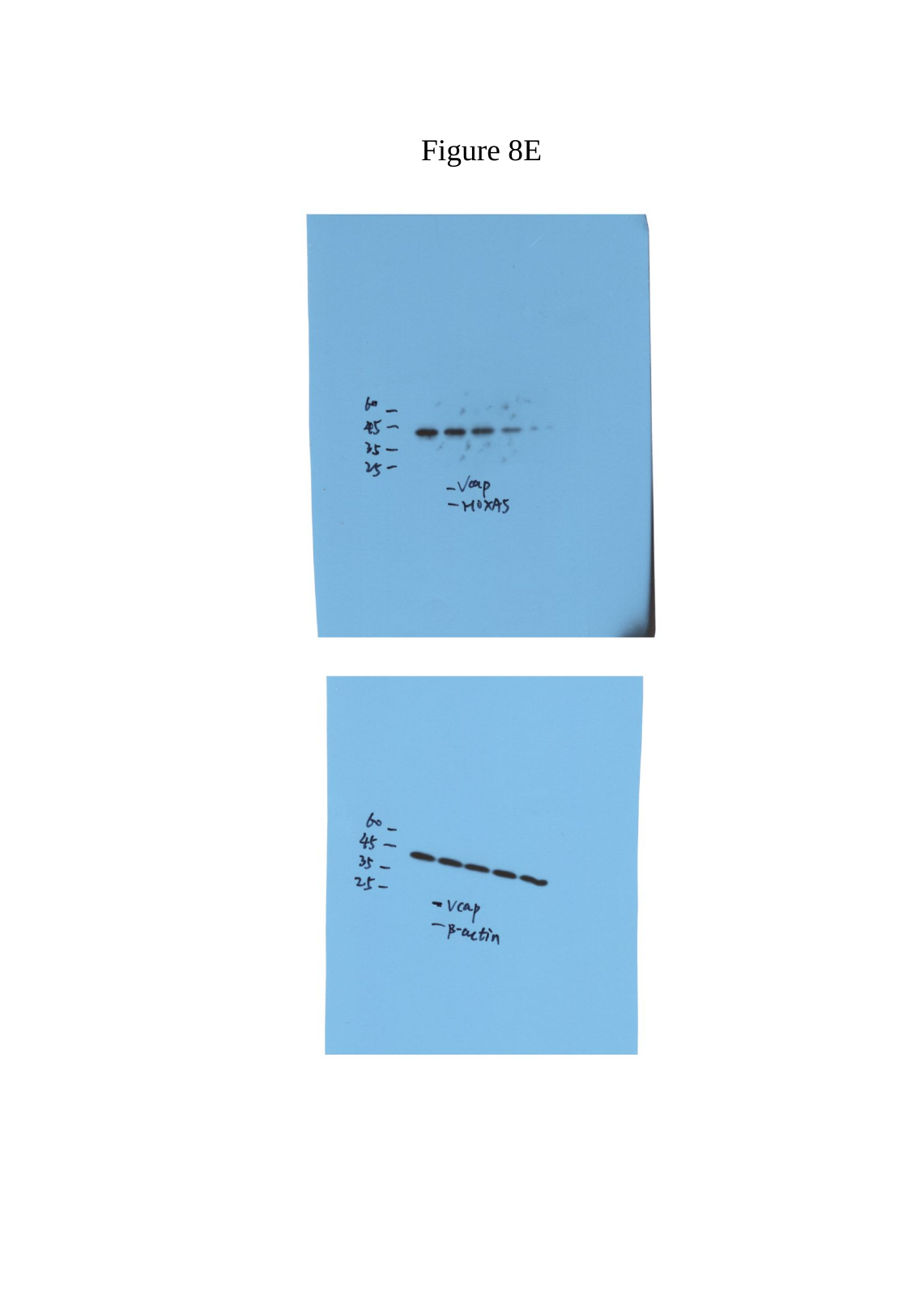

Figure 8E

## Slide 54
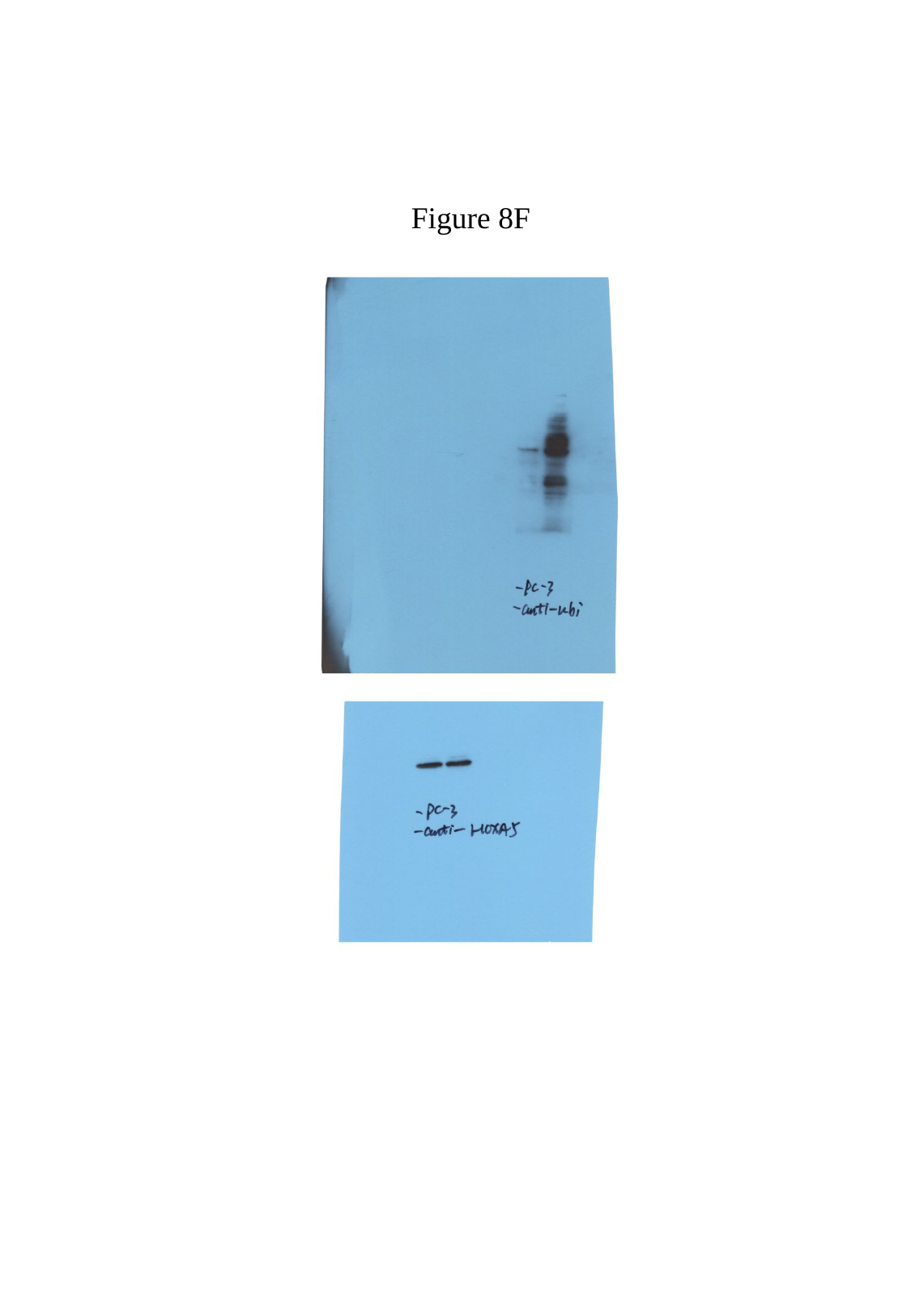

Figure 8F

## Slide 55
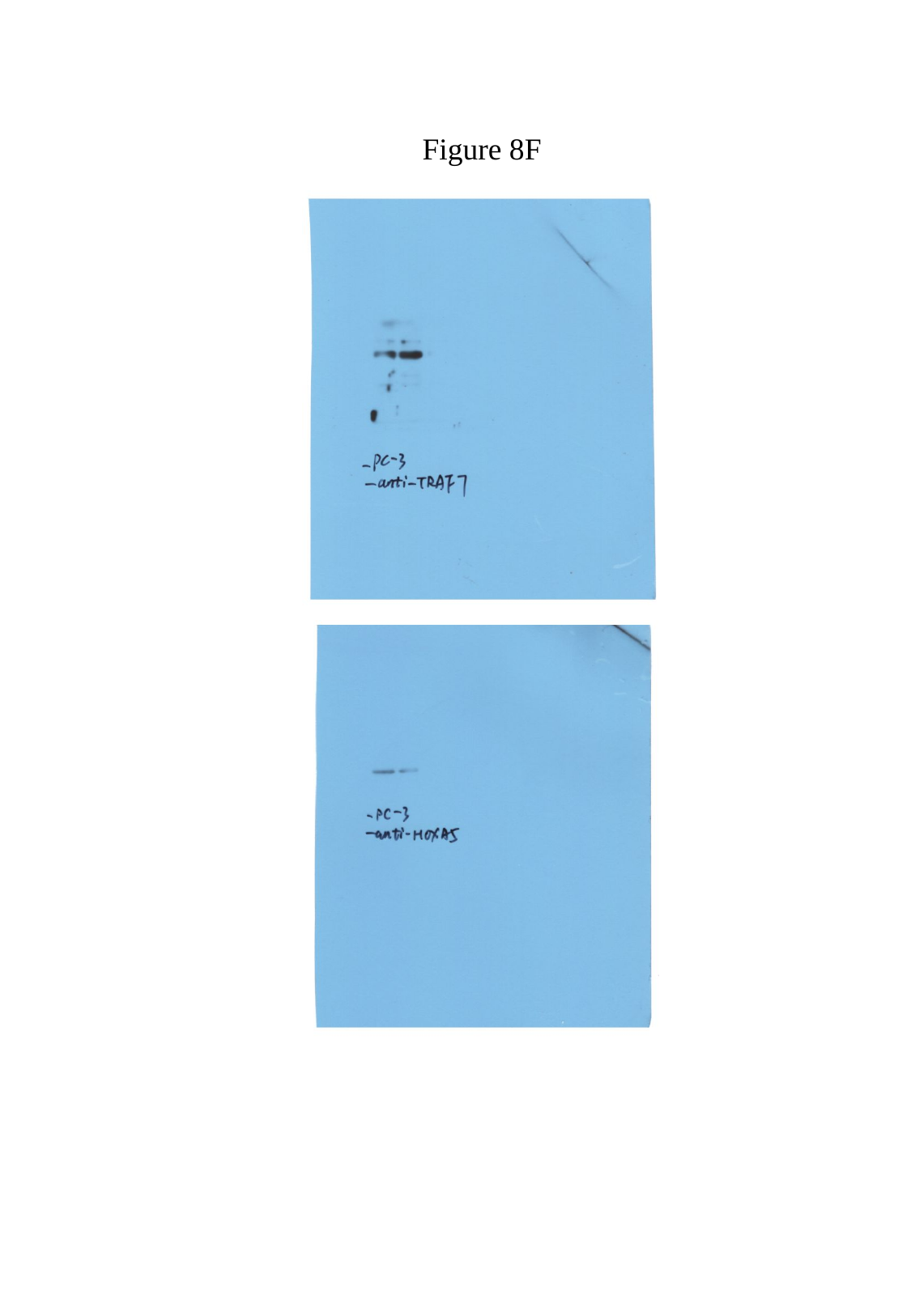

Figure 8F

## Slide 56
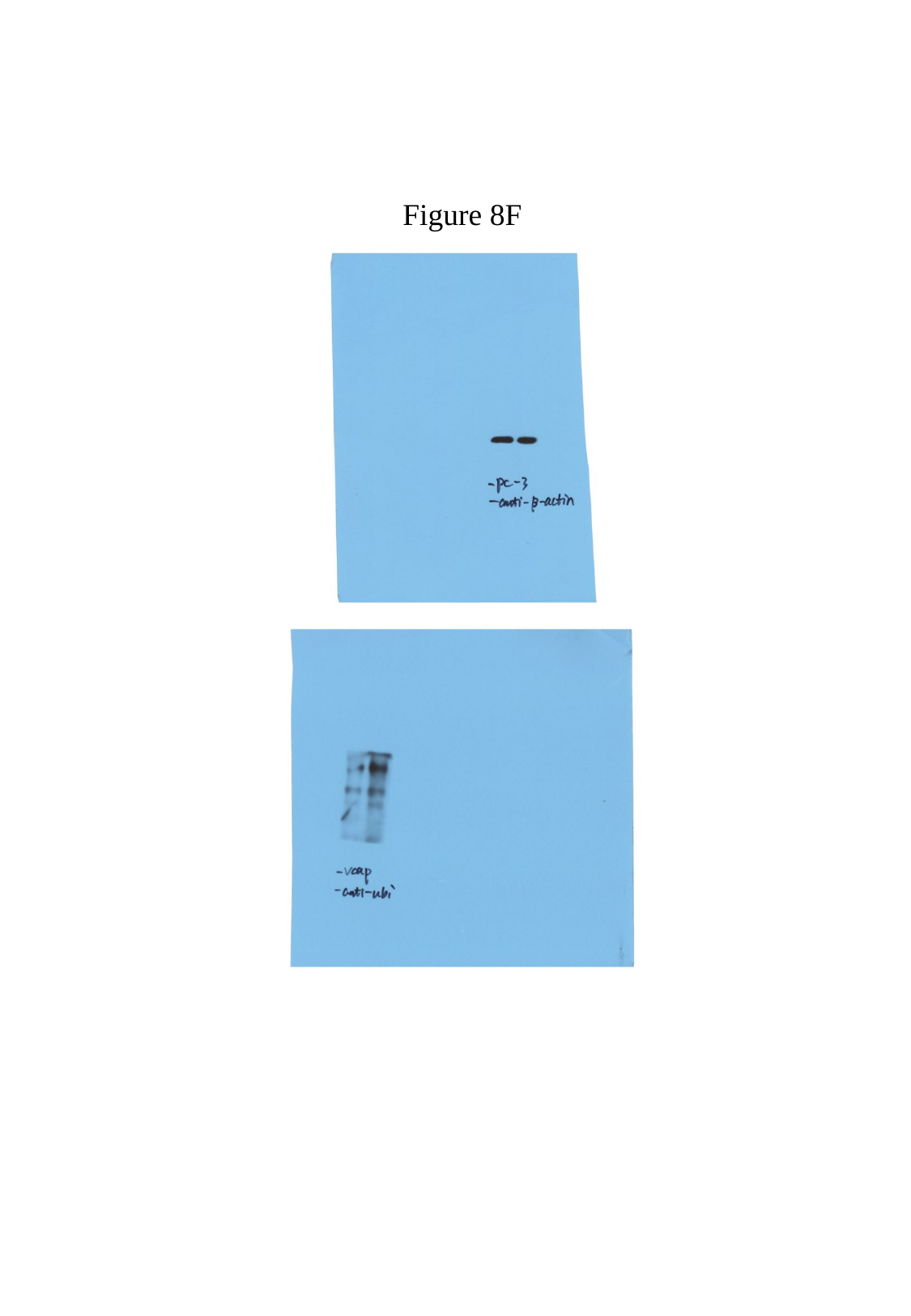

Figure 8F

## Slide 57
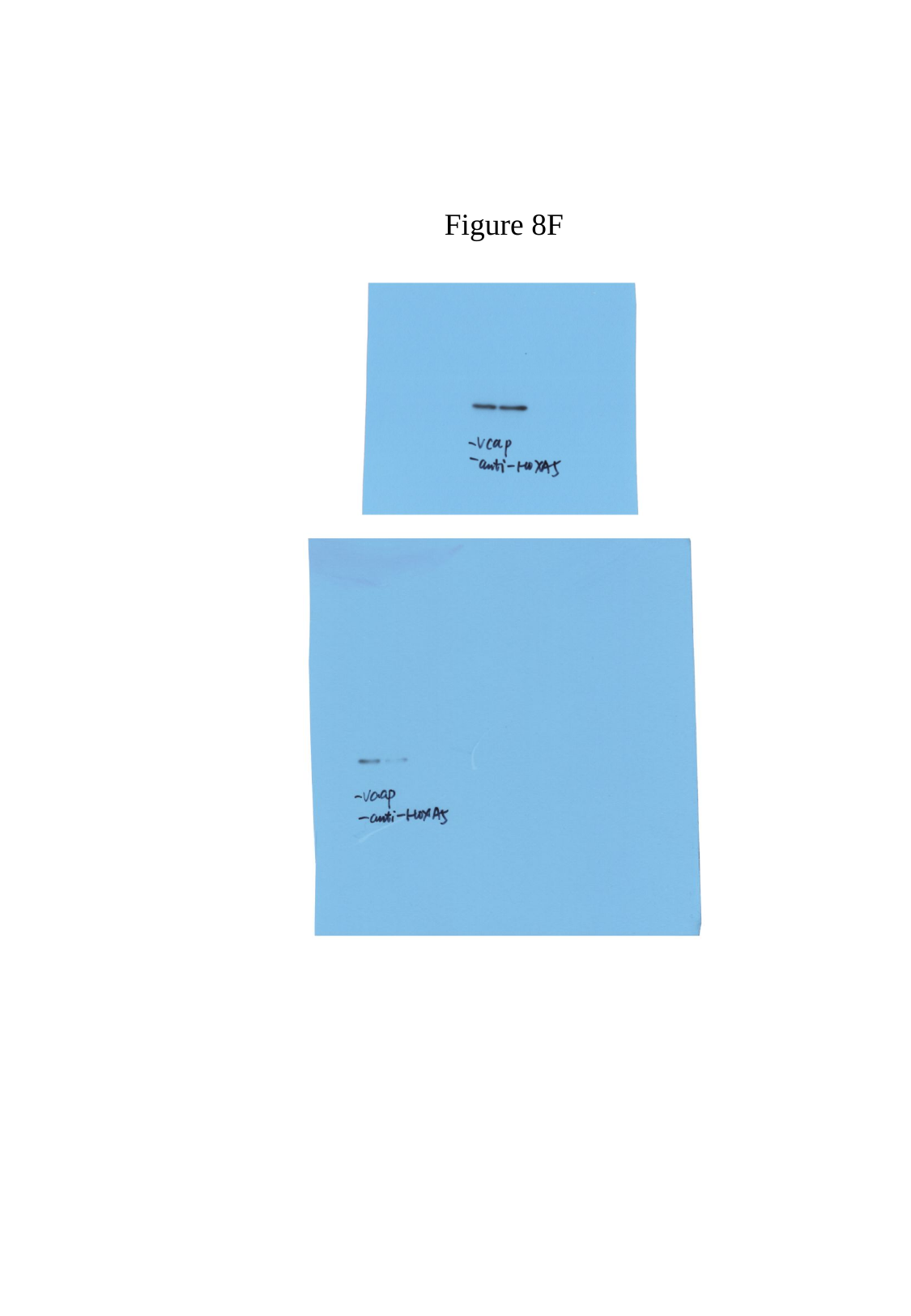

Figure 8F

## Slide 58
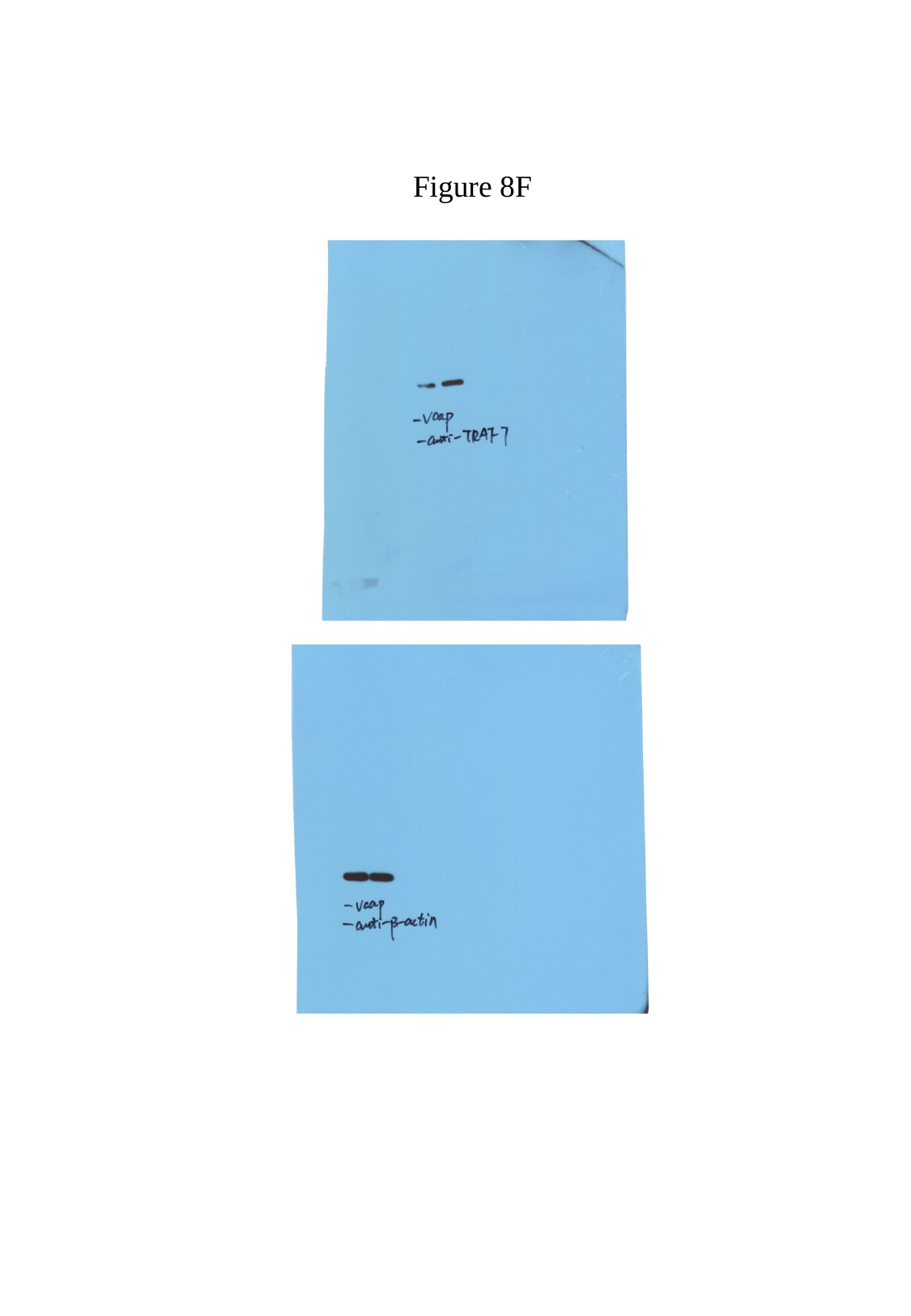

Figure 8F

## Slide 59
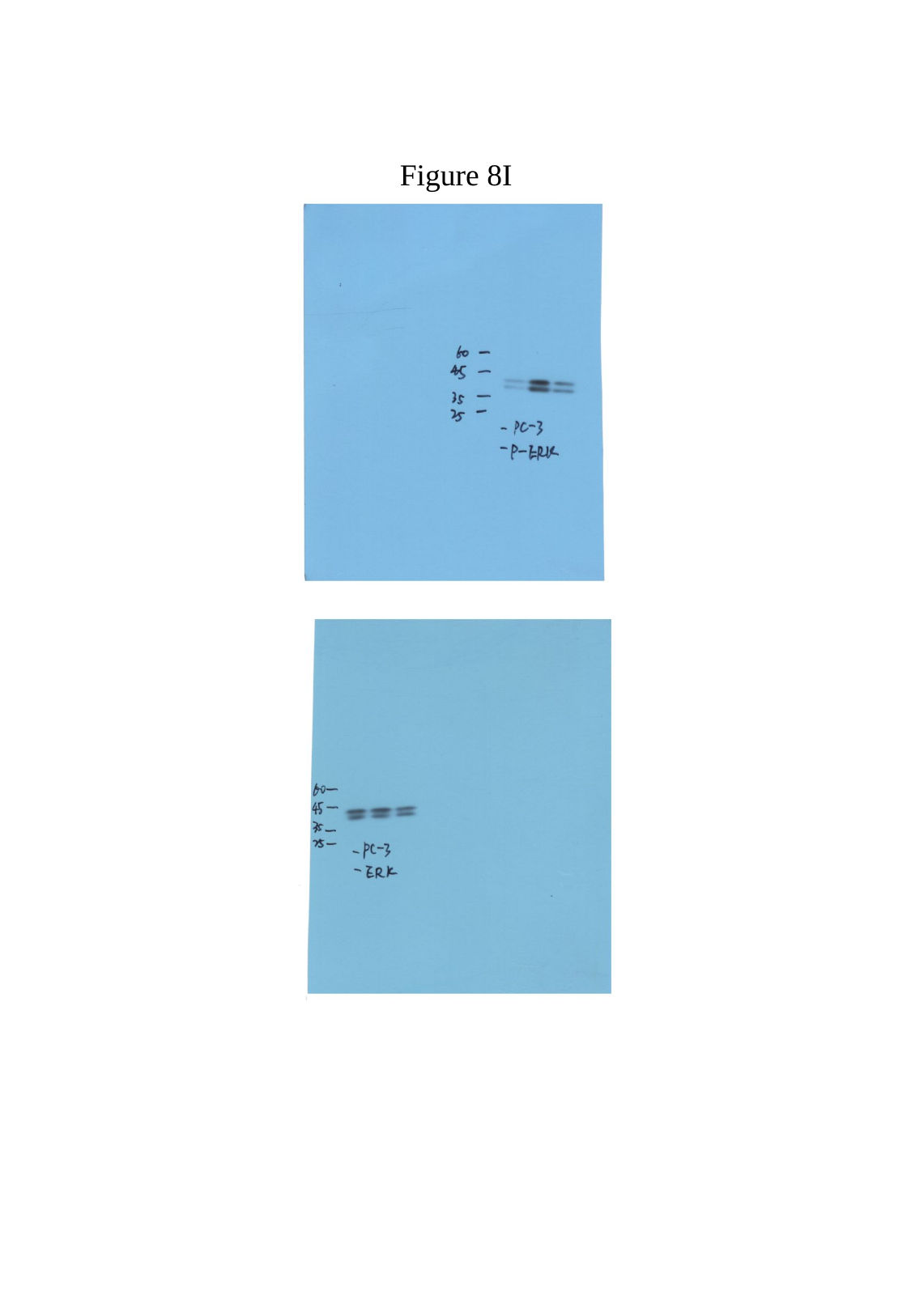

Figure 8I

## Slide 60
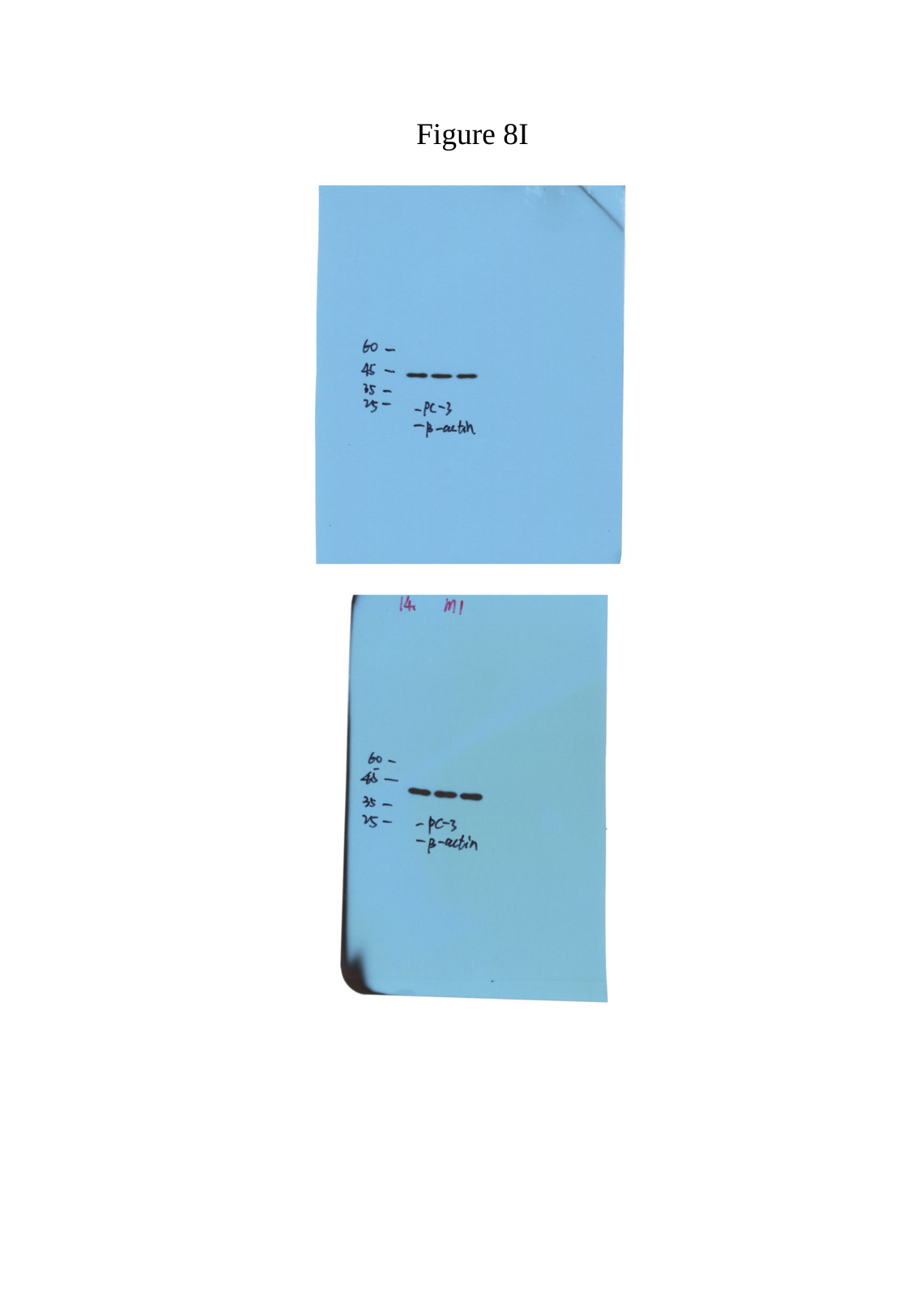

Figure 8I

## Slide 61
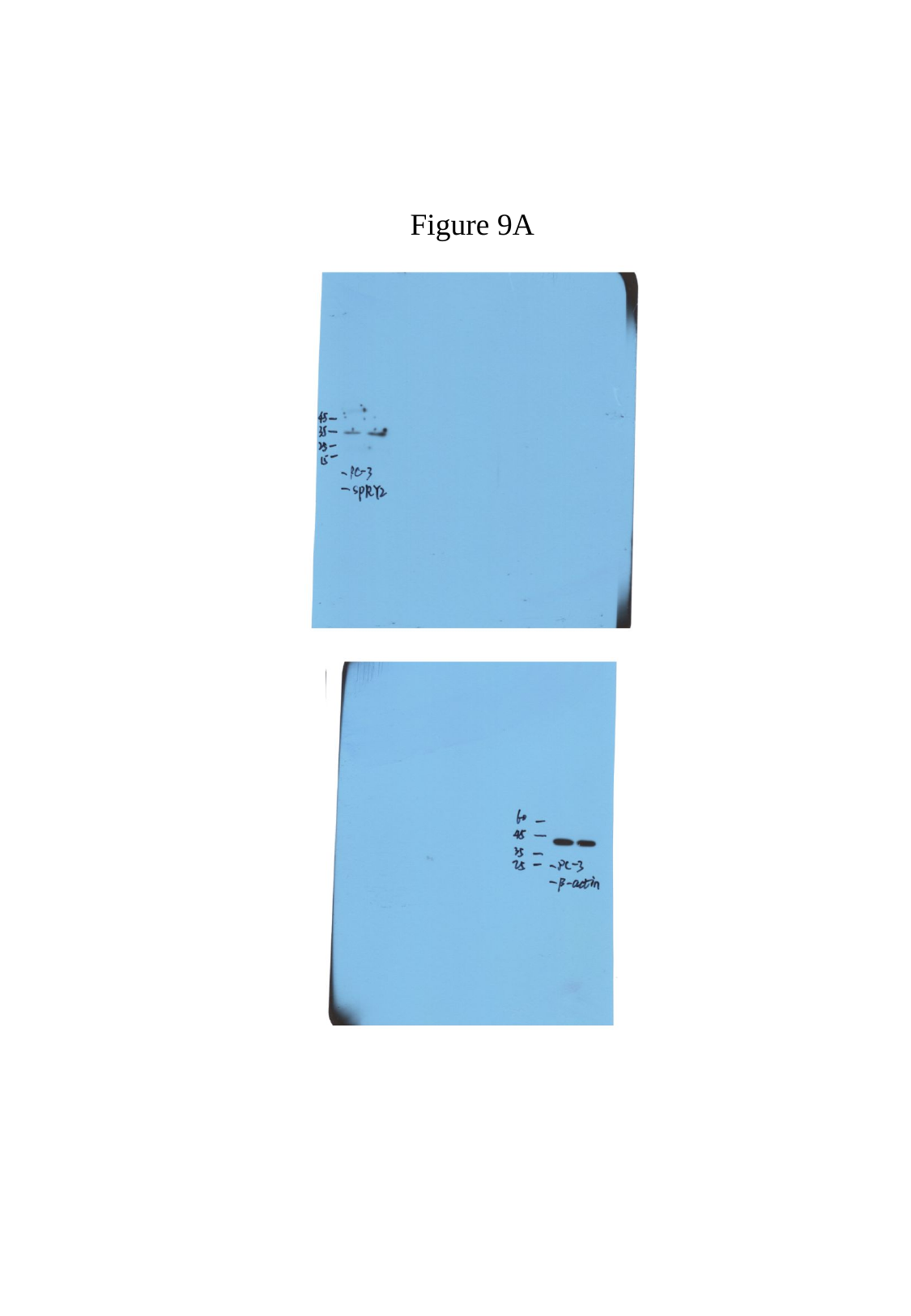

Figure 9A

## Slide 62
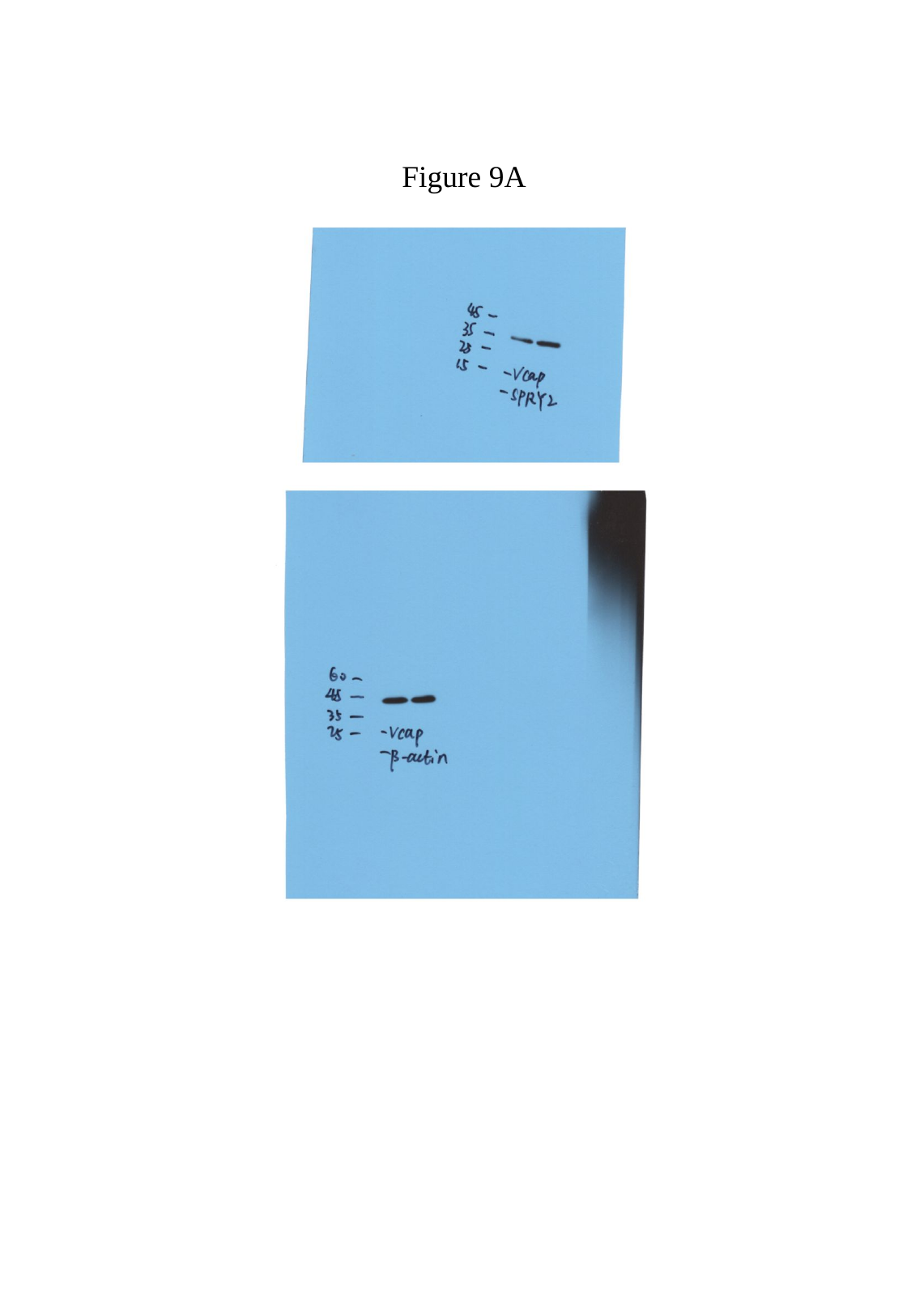

Figure 9A

## Slide 63
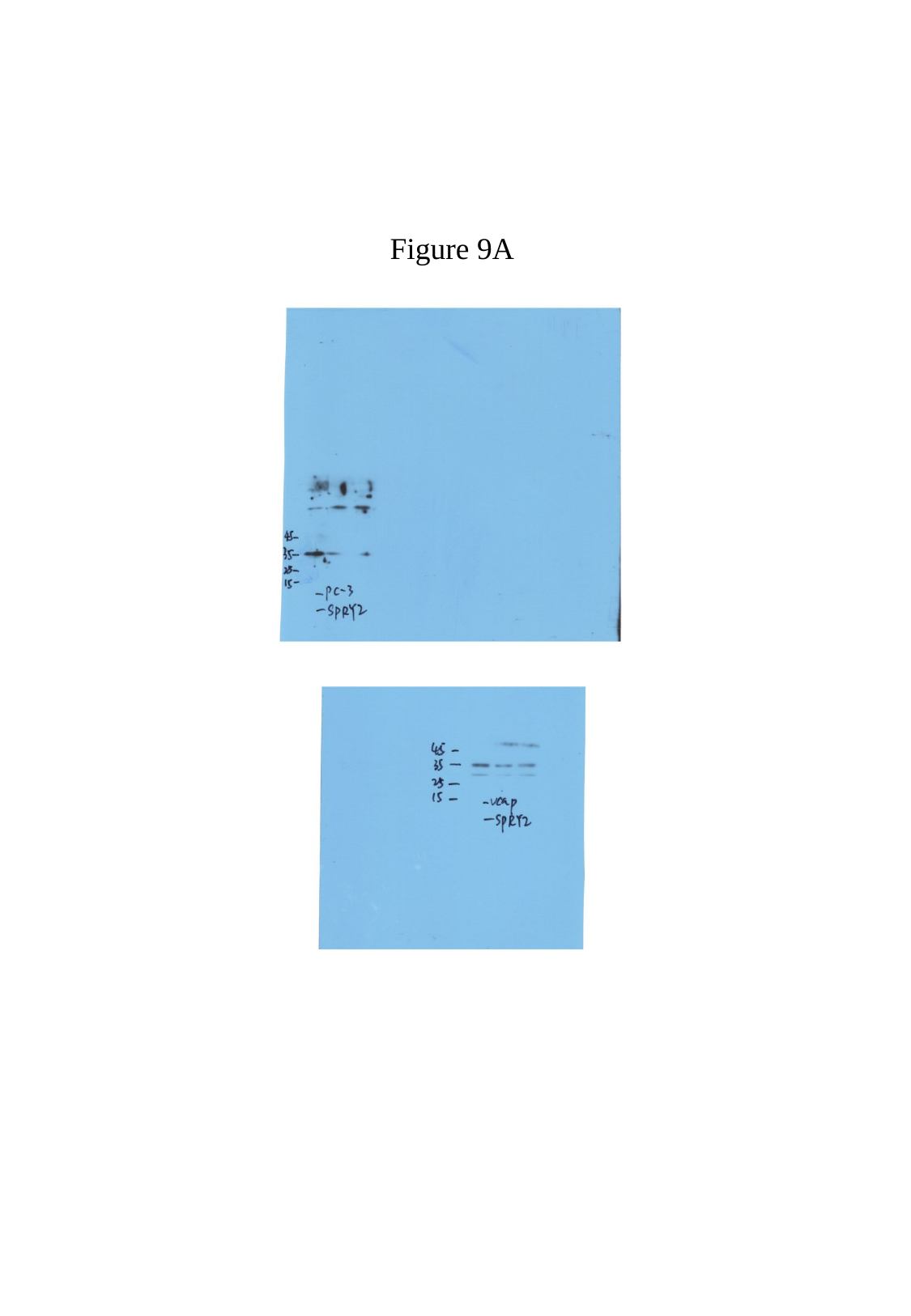

Figure 9A

## Slide 64
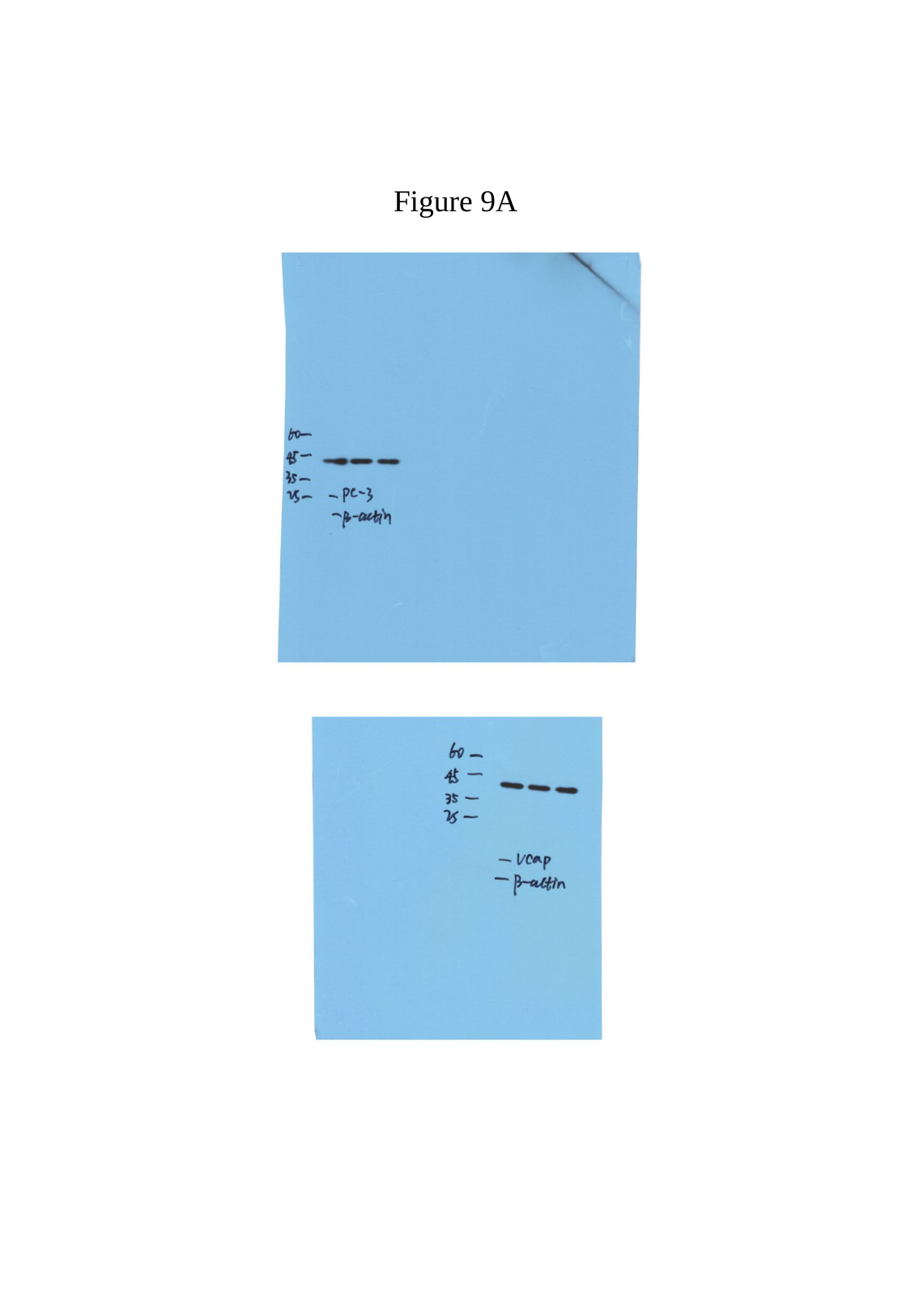

Figure 9A

## Slide 65
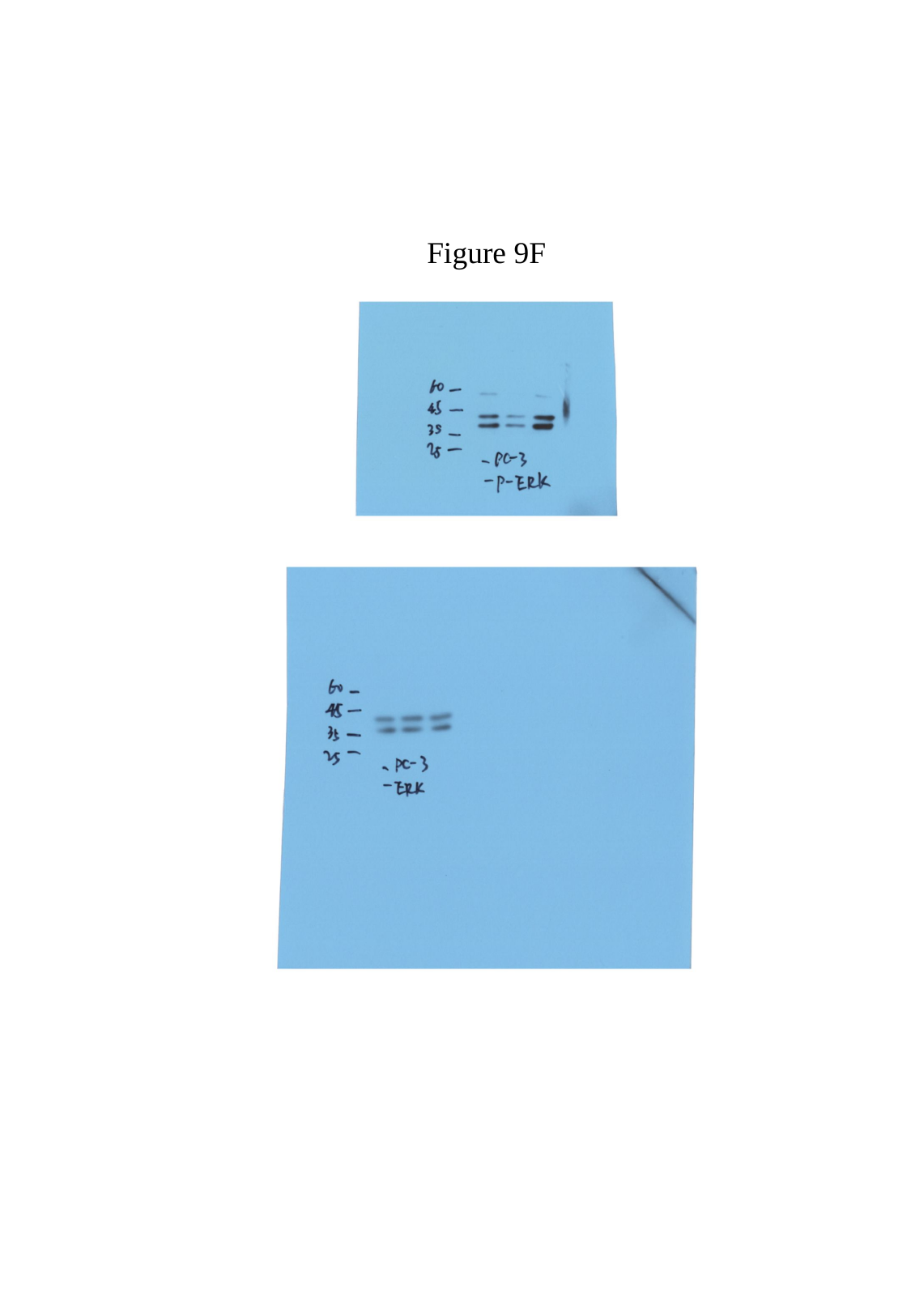

Figure 9F

## Slide 66
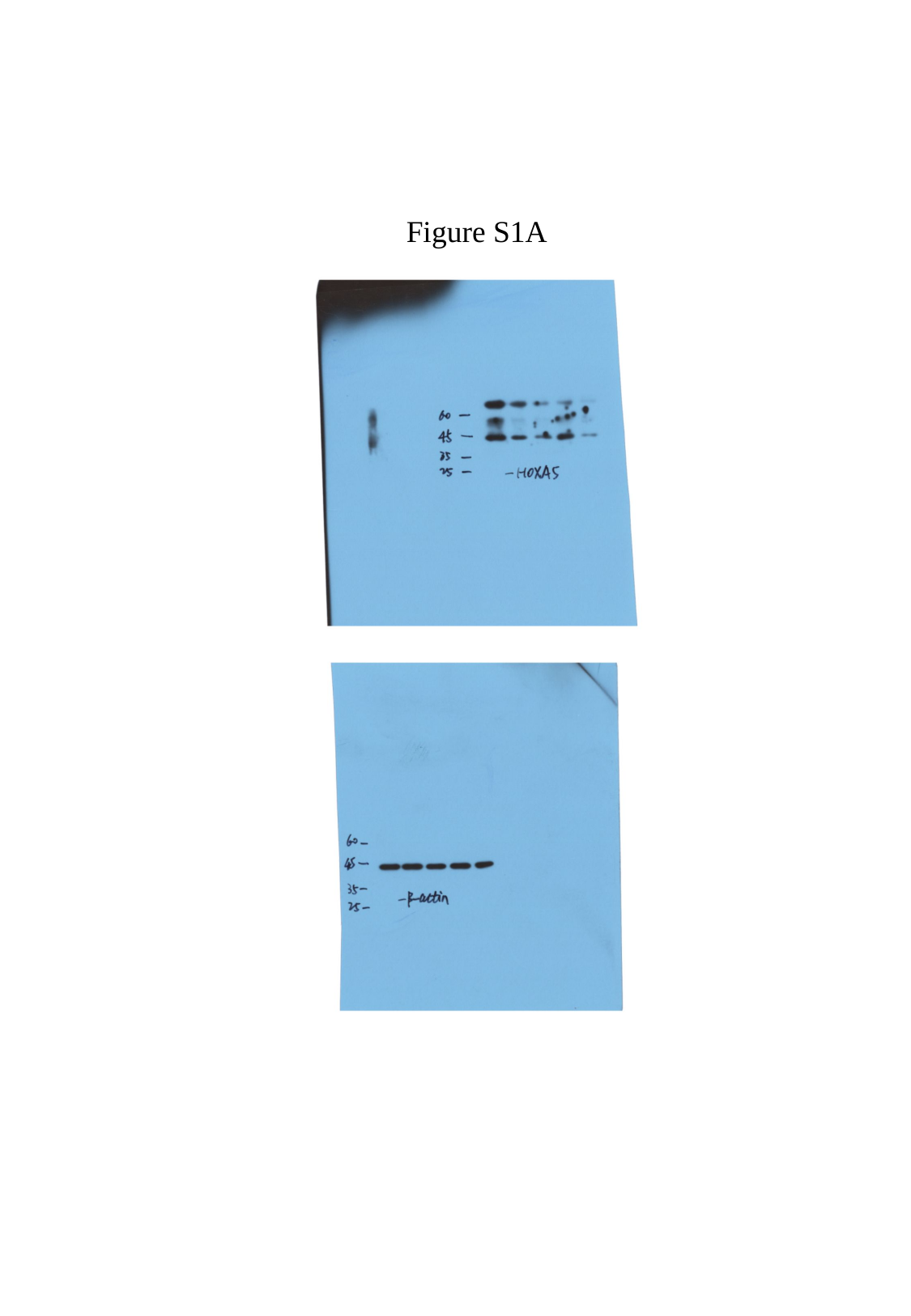

Figure S1A

## Slide 67
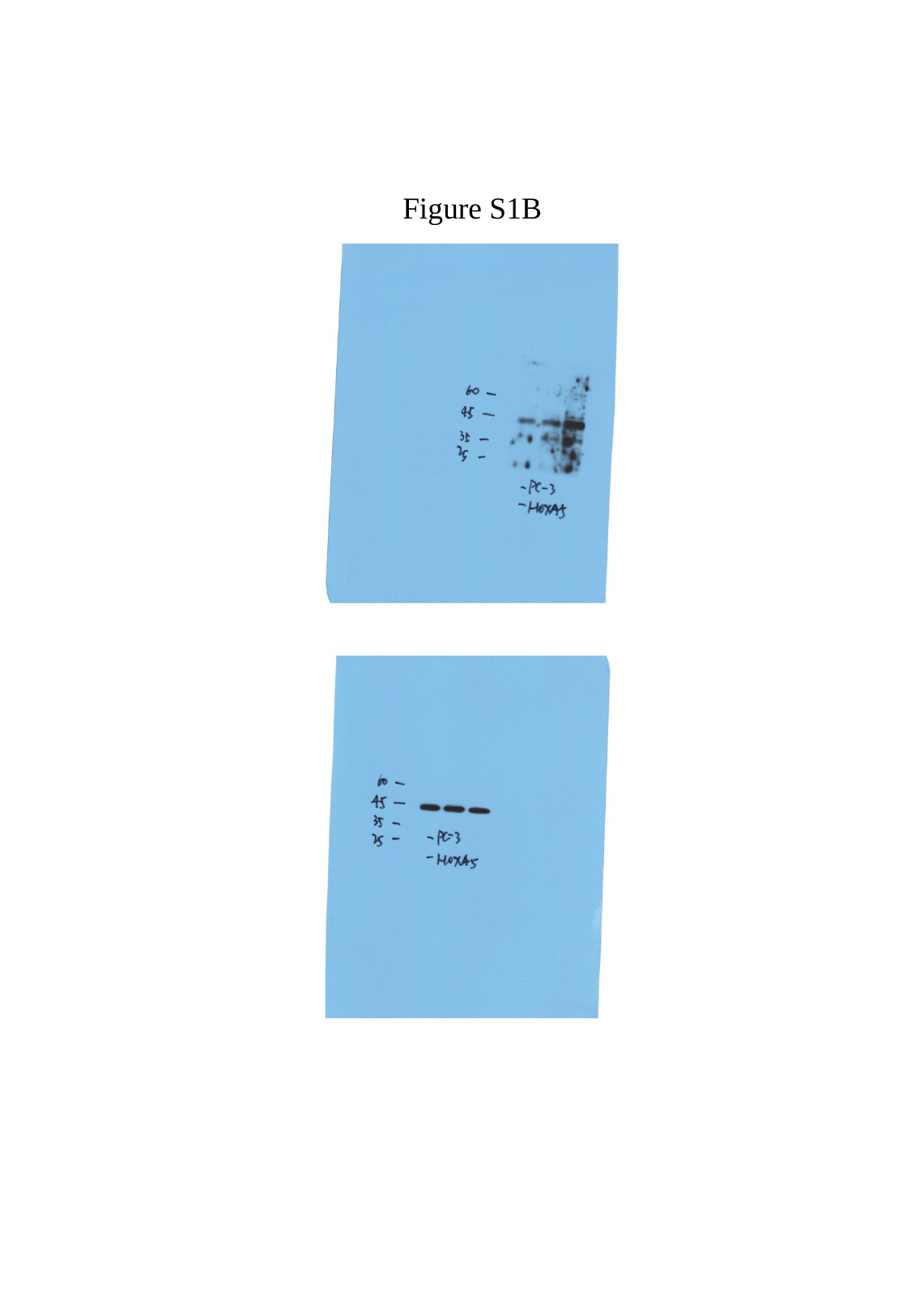

Figure S1B

## Slide 68
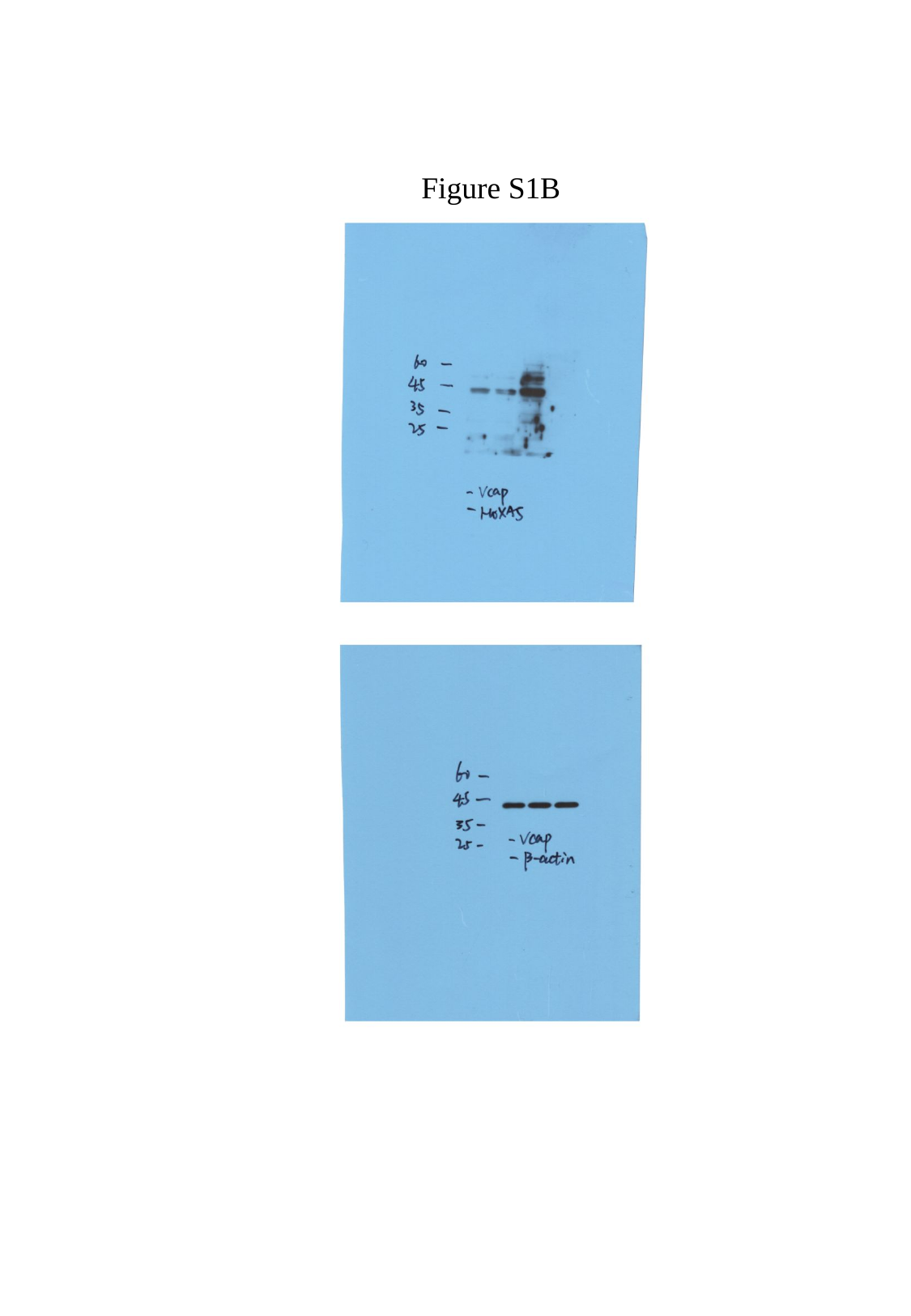

Figure S1B

## Slide 69
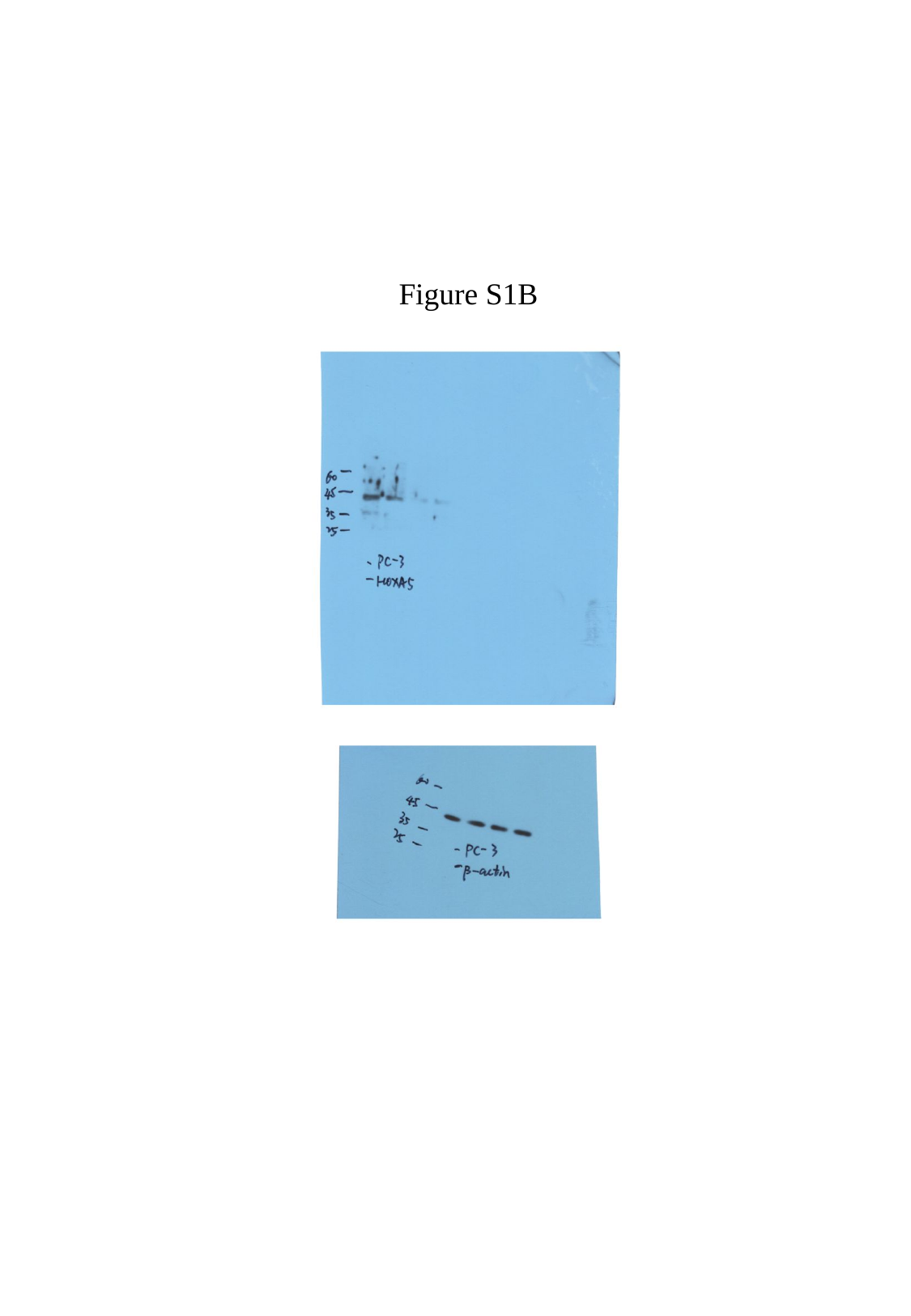

Figure S1B

## Slide 70
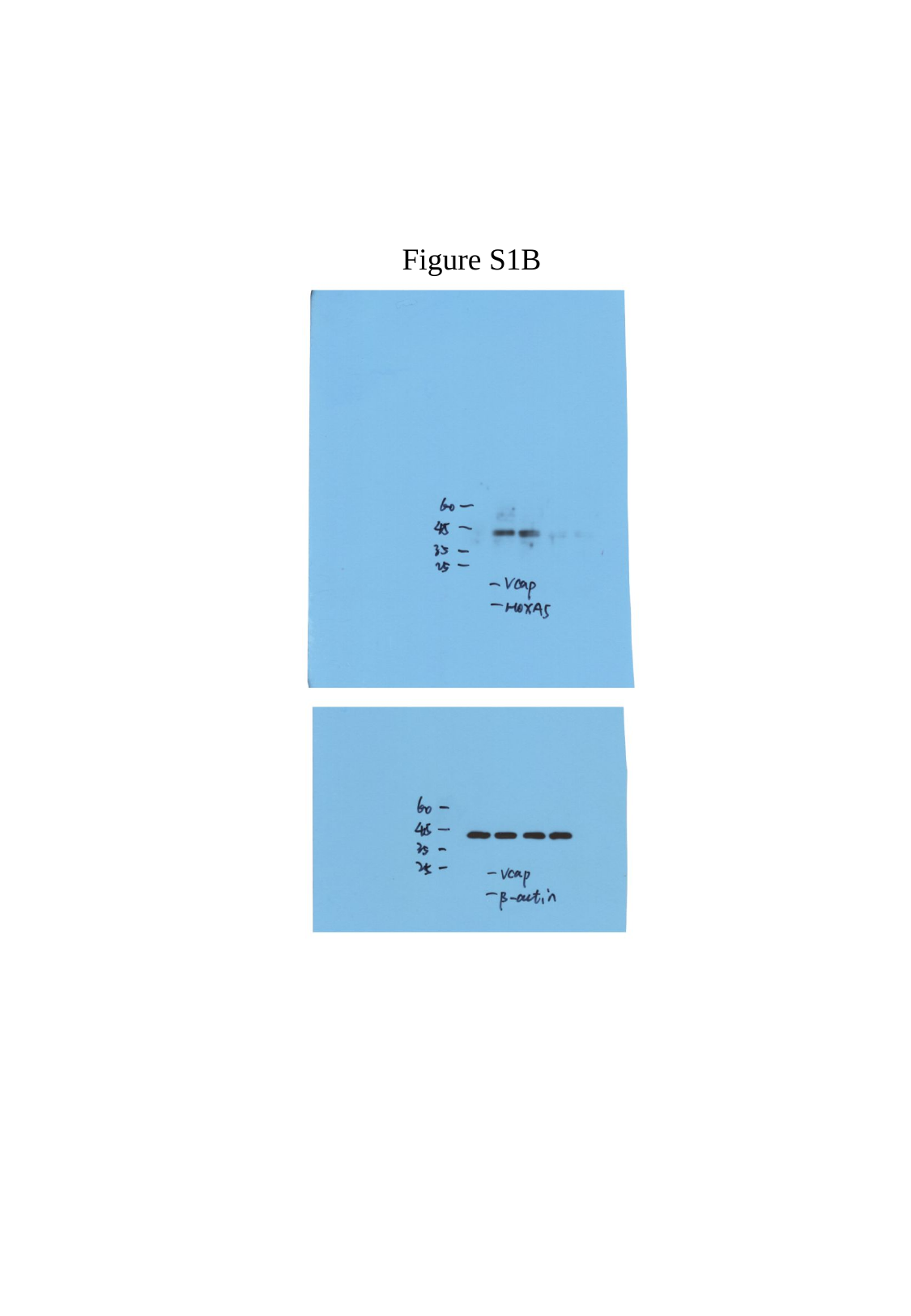

Figure S1B

## Slide 71
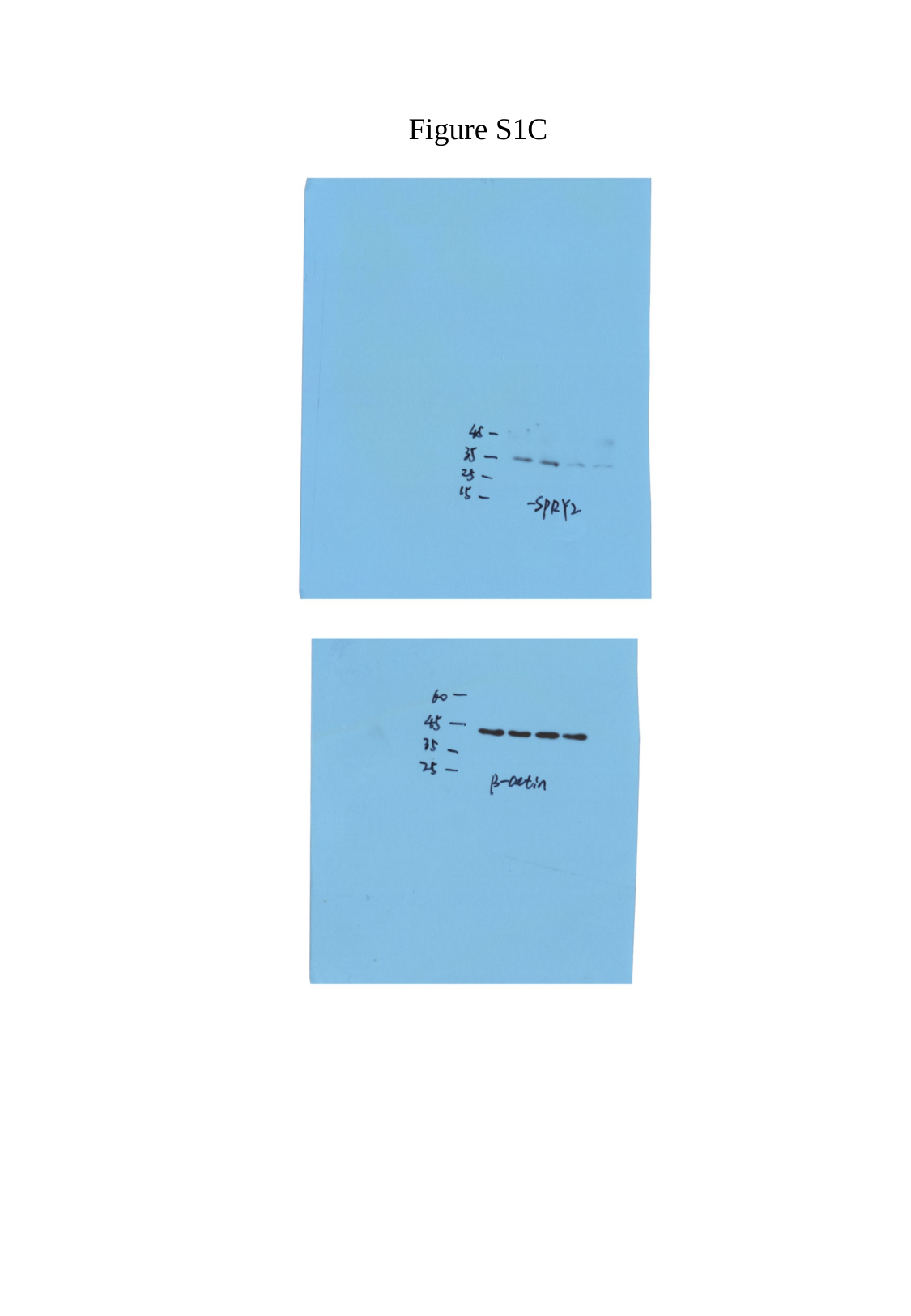

Figure S1C
